# A Drug Safety Briefing (II) in Transplantation from Real-World Individual Pharmacotherapy Management to Prevent Patient and Graft from Polypharmacy Risks at the Very Earliest Stage

**DOI:** 10.3390/ph17030294

**Published:** 2024-02-25

**Authors:** Ursula Wolf

**Affiliations:** Pharmacotherapy Management, University Hospital Halle (Saale), Martin Luther University Halle-Wittenberg, 06120 Halle (Saale), Germany; ursula.wolf@uk-halle.de

**Keywords:** transplantation, polypharmacy, individual pharmacotherapy management, medication review, graft injury, graft outcome, patient outcome, adverse drug reactions (ADRs), drug–drug interactions (DDIs), patient safety, drug safety

## Abstract

For early and long-term patient and graft survival, drug therapy in solid organ and hematopoietic stem cell transplantation inevitably involves polypharmacy in patients with widely varying and even abruptly changing conditions. In this second part, relevant medication briefing is provided, in addition to the scores defined in the previously published first part on the design of the Individual Pharmacotherapy Management (IPM). The focus is on the growing spectrum of contemporary polypharmacy in transplant patients, including early and long-term follow-up medications. 1. Unlike the available drug–drug interaction (DDI) tables, for the first time, this methodological all-in-one device refers to the entire risks, including contraindications, special warnings, adverse drug reactions (ADRs), and DDIs. The selection of 65 common critical drugs results from 10 years of daily IPM with real-world evidence from more than 60,800 IPM inpatient and outpatient medication analyses. It includes immunosuppressants and typical critical antimicrobials, analgesics, antihypertensives, oral anticoagulants, antiarrhythmics, antilipids, antidepressants, antipsychotics, antipropulsives, antiemetics, propulsives, proton pump inhibitors (PPIs), sedatives, antineoplastics, and protein kinase inhibitors. As a guide for the attending physician, the drug-related risks are presented in an alphabetical overview based on the Summaries of Product Characteristics (SmPCs) and the literature. 2. Further briefing refers to own proven clinical measures to manage unavoidable drug-related high-risk situations. Drug-induced injuries to the vulnerable graft and the immunosuppressed comorbid patient require such standardized, intensive IPM and the comprehensive preventive briefing toolset to optimize the outcomes in the polypharmacy setting.

## 1. Introduction

There are well-established drug–drug interaction (DDIs) risks for the calcineurin inhibitors (CNIs), cyclosporine A (CsA) and tacrolimus (TAC), and almost analogously for the mammalian target of rapamycin inhibitors (mTORIs), everolimus (EVR) and sirolimus (SIR), to be considered, as listed throughout the transplantation decades in the literature. Their adverse drug reactions (ADRs) with risks of serious infections, renal, neurologic, and further toxicities and malignancies predominantly depend on their small therapeutic window and, therefore, inevitably require therapeutic drug monitoring (TDM). The risk of ADRs becomes even enhanced with a broad spectrum of concomitant drugs and transient or persistent intestinal or hepatic dysfunction with impaired P-gp-efflux, CYP34A metabolism, and drug elimination capacity [1,2,3,4,5,6,7,8]. In the first part of this series on Individual Pharmacotherapy Management (IPM) in posttransplant polypharmacy, the design of a self-established IPM procedure in conjunction with the TDM of the immunosuppressants has been published [9]. The necessity of the constant focus on the defined patient scores and medication scores as the basis of the IPM was outlined.

The relevance of the medication risk is already obvious in the pretransplant patient requiring liver transplantation for acute hepatic failure. Drug-induced hepatotoxic injuries cause 15% of cases of acute hepatic failure [10]. As from the United Network for Organ Sharing (UNOS) liver transplant database study, acute hepatic necrosis, resulting from acetaminophen (APAP) alone, or in combination with another drug, accounted for 49%; in the non-APAP group, the most frequently implicated drugs were isoniazid, propylthiouracil, phenytoin, and valproate, with 17.5 to 7.3% [10]. Severe drug-induced liver injury (DILI) is the most common identifiable cause of acute liver failure in the United States (US) [10]. According to the results of a US prospective multicenter study, the pattern in most frequent apparent causes of acute liver failure has changed, and APAP overdose and idiosyncratic drug reactions have replaced viral hepatitis [11]. Focusing on liver safety following liver transplantation, a Spanish research group emphasizes in their review the urgent need for the awareness of hepatotoxic ADRs to prevent posttransplant DILI. They highlight the need for further research and collaboration, as this topic remains under-recognized, especially in terms of associated risk factors and higher vulnerability of the transplant [12]. The regularly updated LiverTox database site provides unbiased clinical and research information on drug-induced liver injury [13] to be looked up for single agents.

A Dutch multicenter study identified 14 drug groups to be associated with a higher risk of acute kidney injury (AKI) after adjustment for confounding [14].

These are only the peaks. However, in parallel, we have to assume an extensively higher portion of patients suffering from less severe stages or non-identified drug injuries. According to a review on drug-induced nephrotoxicity, drugs cause approximately 20% of community- and hospital-acquired events of acute renal failure. In the elderly adults, the incidence of drug-induced nephrotoxicity reaches 66% [15]. This patient group may be partly comparable with similar vulnerable kidney transplant patients. One or more common pathogenic mechanisms have been observed in drug-induced nephrotoxicity, such as altered intraglomerular hemodynamics, tubular cell toxicity, acute or chronic interstitial nephritis, inflammation, crystal nephropathy, rhabdomyolysis, and thrombotic microangiopathy [15]. For preventive measures, knowledge of the drugs causative of these ADRs is essential.

The unavoidable polypharmacy post transplantation increases with the pre-existing concomitant comorbidities and metabolic disorders of the individual patient, the latter being even further iatrogenically promoted by the immunosuppressive regimens themselves, as we already addressed in the 1990s [16,17,18,19,20]. In 1996, we referred to the dilemma of the iatrogenic medication risks as “chronic allograft destruction versus chronic allograft rejection” [21]. This has been confirmed during the years hereafter in several studies, including a review by Halloran [22]. As polypharmacy affects all types of transplantation in both, the acute and the long-term posttransplantation periods, the precise individual patient condition must always be contextualized with the medication scores to prevent harm to the graft and patient from the earliest stage possible. For this purpose, focusing on the elimination of any drug-induced iatrogenic graft and patient injury, we conceptualized an individual pharmacotherapy management (IPM), being implemented in addition to the TDM of the immunosuppressants. The IPM concept has been introduced and published ahead of this as the first part of this drug safety series [9]. On a daily basis for more than a decade of IPM experience, from over 60,800 authors’ own medication reviews in polypharmacy, a broad spectrum of aspects has arisen to be considered in order to eliminate drug-induced iatrogenic injuries to the allograft and the patient. Yet, it is almost impossible to study all the Summaries of Product Characteristics (SmPCs) up to even >100 pages for a single drug in the clinical routine. But there are no overviews, except for DDIs or toxicity risks, available for the practice of challenging polypharmacy posttransplantation. 

The aim of this second and complementary part of the series on drug safety in transplant polypharmacy is to provide instruments for the immense challenges posed by the IPM-defined medication scores. The spectrum of frequently coadministered medications in polypharmacy after transplantation is extremely broad, as the author has experienced over 10 years of IPM and 21 years of daily posttransplant TDM reviewing of immunosuppressants. Every transplant physician needs to be aware and educated not only on the DDIs, but also on the ADRs, contraindications, and warnings of concomitant medications in the polypharmacy context. There is no standard procedure to systematically review the individual risks of polypharmacy, despite the particular vulnerability of the transplant patient and graft. And there is no comprehensive resource covering the risks of the current common polypharmacy agents in total. This would mean an overdue and helpful tool for clinical and outpatient physicians is needed. Furthermore, from the author’s own experience, this is needed not only for the transplant team, but also for the various disciplines that are unfamiliar with the risks of posttransplant polypharmacy, despite treating transplant patients in the long-term follow-up for eventually upcoming surgeries and diseases. Therefore, this second paper is intended to provide additional practical devices for the awareness of these almost unmanageable challenges of defined medication scores. As an extract of the clinically relevant aspects of today’s commonly coadministered drugs, covering contraindications, ADRs, DDIs, and drug-specific warnings, it is intended to be a useful tool for obtaining initial information in daily practice, rather than an unmanageable, time-consuming study of multiple SmPC brochures of 10 to >100 pages each.

As a practical combined toolset, the latest SmPCs covering drug information from clinic and research and further literature data are extracted and tabulated alphabetically. Clinical IPM countermeasures and preventive approaches based on 10 years of IPM experience are presented to overcome unavoidable high-risk situations, such as those indicated by the antibiogram or the antimicrobial resistance chart, which requires the risky coadministration of targeted antimicrobials without effective alternatives. 

## 2. Methods

### 2.1. IPM Concept and Implementation of a Digital Interdisciplinary Networking Strategy [9]

**Figure 1:**
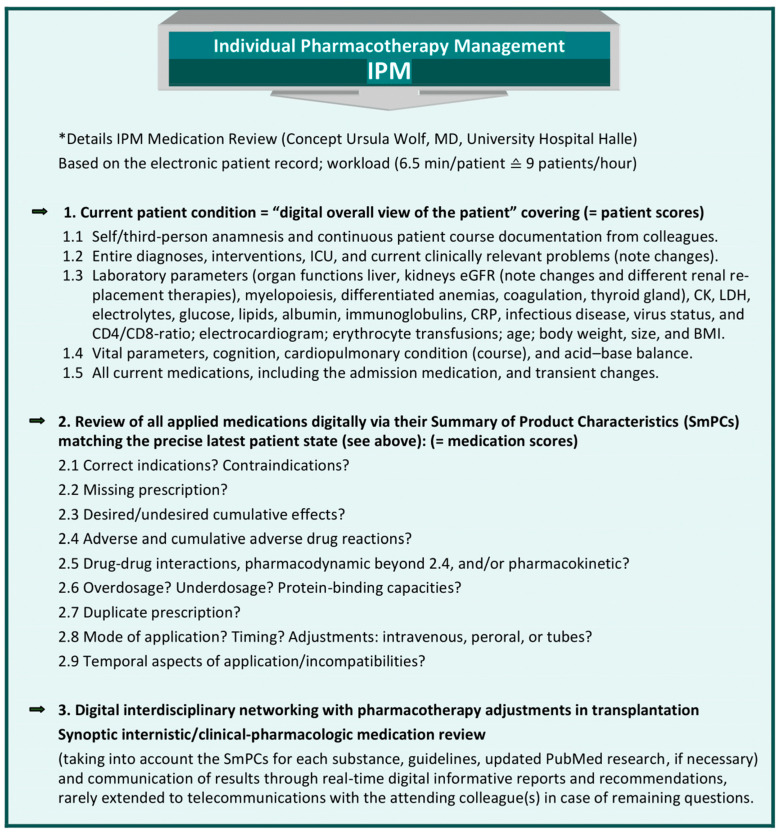
Comprehensive, reproducible IPM, based on the electronic patient record (adapted from [9], Pharmaceutics, 2023). * IPM (applied patient and medication scores), based on the electronic hospital patient record at Halle University Hospital, conceptualized, implemented, and practiced by Wolf, MD, Head of Pharmacotherapy Management Department, Specialist in Internal Medicine, with expertise in Clinical Pharmacology and Transplantation, performed > 60,800 individual medication reviews.

At the University Hospital Halle (UKH), the IPM procedure has been designed and implemented for 10 years [Figure 1]. The IPM scores were determined by the author with disciplinary education and 12 years of experience in internal medicine, plus 6 years in clinical pharmacology and 6 years in transplantation. She designed and has administered the IPM, with up to >60,800 of her own IPM reviews. The scores are primarily based on her daily experience with multimorbid patients in polypharmacy. Their disease condition and laboratory parameters, with the individual impact on drug and patient safety, led to the patient scores. In addition to DDIs, the medication information required for an adequate and comprehensive individual medication analysis must also comprise ADRs, contraindications, dosing, and drug-specific warnings. These are reflected by the medication scores defined for the IPM. The IPM is conducted as a synopsis of internal medicine and clinical pharmacology. To ensure reproducibility for other healthcare professionals, the systematic standardized format is precisely outlined. The IPM is combined with the individual trough-level TDM of immunosuppressants in patients undergoing solid organ or allogenic hematopoietic stem cell transplantation (HSCT) [9]. It provides continuous interdisciplinary networking, based on each patient’s electronic medical record from the start of transplantation. The reproducible IPM protocol refers to the most accurate current clinical condition of the patient with respect to his organ functions and vital parameters. In order to match the prescribed medications to the patient’s degradation and elimination capacities, taking into account the real-time concurrently manifest pharmacokinetic DDIs, the entire medication list is analyzed based on the SmPC for ADRs, including pharmacodynamic DDIs, contraindications, warnings, and dosages. Additional tools are used in complicated situations, such as continuous renal replacement therapy (CRRT) [23,24,25], and for further interaction checks in the case of open questions [26,27] and PubMed research.

### 2.2. Briefing Toolset on Relevant IPM Medication Scores and Preventive Countermeasures to Avoid Iatrogenic Patients and Graft Injuries

First, a research on overviews of medication-related injuries in transplants and patients in PubMed was performed. There have been constantly ongoing case reports and lists of potentially nephrotoxic or hepatotoxic agents and of DDIs, especially with a focus on the CNIs and mTORIs throughout the decades of transplantation. However, from the review, there are no comprehensive methodological approaches to standardize a drug safety procedure in transplantation to eliminate the severe risks, which result from the individual polypharmacy setting. 

1. To provide devices for the designed and approved IPM procedure with reference to the defined IPM medication scores for clinical and ambulatory practices, the contemporary drug combinations in early and long-term posttransplant polypharmacy are addressed. The focus is on the most common critical coadministered drugs experienced by the 10 years of IPM and scoping reviewed via their latest SmPCs and PubMed research for each active ingredient. A tabulized overview is to provide a broader spectrum of drug-related risks, unlike the available DDI lists. In this briefing, ADR plus DDI plus contraindication plus drug-specific warnings are included to enable the IPM performance according to the defined medication scores, and to adapt them to the patient scores. The author’s own real-world insights from daily IPM in organ transplantation and HSCT with more than 60,800 self-conducted IPMs force to address a wide spectrum of critical medication entities to preserve grafts and patients from iatrogenic drug-induced injury. In consequence, the tabulized relevant medications comprise the four classes of immunosuppressive maintenance drugs: CNIs TAC and CsA, antiproliferative agents mycophenolate mofetil, mycophenolate sodium, mTOR inhibitors SIR and EVR, and steroid prednisolone, typical critical antimicrobials, analgesics and antipyretics, antihypertensives, ß-blockers, antiarrhythmics, oral anticoagulants, antilipids, antidepressants, antipsychotics, antipropulsives, antiemetics, propulsives, proton pump inhibitors (PPIs), sedatives, antineoplastics, and protein kinase inhibitors. Their tabulized extracted information is intended to provide the essence for the daily routine from each drug’s SmPC brochure, further DDI checks, and PubMed research data. The table format with the alphabetical listing of drugs and their classification into DDIs, ADRs, contraindications, and drug-specific warnings is to provide an easy and most practical way to find them in everyday clinical practice.

It is important to be aware that these aspects do not reflect the entire drug information, nor do they include specific issues, such as hypersensitivity, children, pregnancy, lactation, congenital metabolic malfunctions and intolerances, genetic variants [28,29,30], dysphagia [31], and specified dosing regimens, the latter requiring modification according to each patient’s individual, eventally abruptly changing, degrading/eliminating capacities and comedication. There is no further grading of each referenced risk since the individual patient’s condition and the different medication lists themselves always have a modulating impact.

2. For further briefing from the decade of IPM evidence, the practiced tailored countermeasures are presented. They are to provide preventive tools for frequent risk situations, such as drug-induced renal injury, QTc prolongation, rhabdomyolysis, hemorrhages, wound-healing problems, pain management, and others to avoid harm to patient and graft. 

## 3. Results

### 3.1. IPM in Practice

IPM based on the defined patient and medication scores proved to be the most effective, with a 100% relative reduction in the prevention of, for example, nephrotoxicity and renal impairment as analogously applied in elderly patients [32]. A constant awareness of the real-time interference of drug degradation capacities and patient status, both of which are affected by multiple, often abruptly changing patient and multimedication conditions, is the sine qua non in polypharmacy.

**Figure 2:**
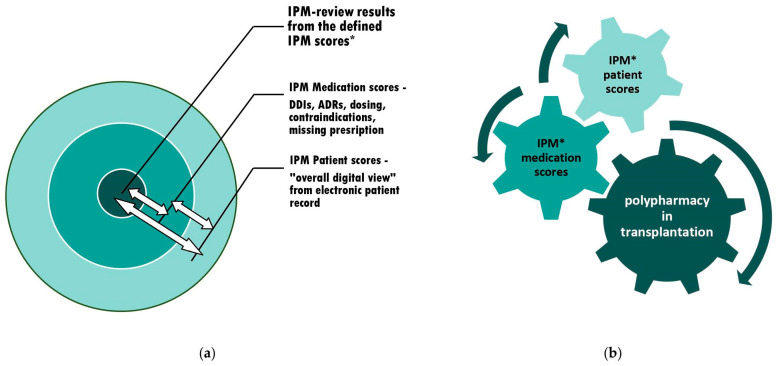
The context of appropriate polypharmacy assessments and subsequent adjustments: (expanded version of [9], Pharmaceutics, 2023) (**a**) The individual circuits affect each other. Therefore, interference monitoring is required. (**b**) The optimization of polypharmacy by continuous synoptic contribution and the adaptation to confounding risks from varying current patient condition and altering comedications through simultaneous IPM in real-time. An additional IPM toolset with tabulated medication scores for 65 commonly coadministered medications and a practice-oriented briefing on counteracting risk situations is provided. * IPM scores see Figure 1.

The transplant patient is even more vulnerable: the narrow therapeutic window of his/her CNI or mTORI is very susceptible to comedications that might either cumulatively increase the ADR risks of the immunosuppressant and other drugs, and/or, furthermore, often interact with the immunosuppressant’s and other drugs’ effects and ADRs in a pharmacodynamic and/or pharmacokinetic manner. Taking this into account in the individual patient for each drug remains an enormous professional challenge for the treating physician, either in the acute or long-term posttransplant setting [Figure 2]. 

With a daily routine and a comprehensive electronic patient record the IPM takes an average of only 6.5 min. It enables seamless, digital, real-time interdisciplinary networking, and is also applicable in addition to any TDM-based drug dosing [9]. The resulting recommendations for the attending colleagues are communicated digitally in real time, and in rare cases also telemedically for remaining open questions. The interprofessional applicability and reproducibility of the IPM procedure by instructed clinical pharmacists has been tested positive. There is no need for on-site expertise or capacity, as the digital basis enables real-time IPM via the electronic health record from any remote location.

### 3.2. Tabulated Extracts as the Relevant Medication Scores of Common Critical Coadministered Drugs in Posttransplant Polypharmacy

In particular, the IPM medication scores require the intensive and time-consuming study of SmPCs, DDI checks, dosing analyses, and possibly additional literature research. Without a daily routine that minimizes these efforts, this may be unmanageable for physicians or pharmacists who are not as familiar with each drug. For this reason, excerpts from the daily IPM drug reviews, based primarily on the updated drug SmPCs, are presented in tabular form in alphabetical order of the drug name for easy navigation [Table 1]. It aims to provide the most important clinically relevant information from the extensive SmPCs of those drugs that are frequently coadministered in the transplant patient. It provides guidance on clinically relevant ADRs, DDIs, and contraindications in a single overview. The drugs selected have been chosen to cover the most common critical prescriptions in posttransplant polypharmacy. The table specifically addresses medications commonly used in either the acute or long-term period after organ transplantation and HSCT, as seen from real-world IPM in these often high-risk settings.

As experienced and emerged from the lack of availability in repeatedly reviewed real-world IPM, the table includes the CNIs and mTORIs, MMF, prednisolone, critical antibiotics, antifungals, antivirals, analgesics, antipyretics, antihypertensives, antilipids, antidepressants, PPIs, antipsychotics, sedatives, propulsives, antipropulsives, antiemetics, antiarrhythmics, anticoagulants, antineoplastics, and protein kinase inhibitors. 

The excerpts from the updated SmPCs include ADRs and DDIs from reported placebo-controlled trials and from post-marketing experience, which are not further graded here. The essence results from the focus on graft and patient toxicity risks from ADRs, DDIs, contraindications, and additional drug-specific issues to be considered [Table 1].

### 3.3. Briefing on Awareness and Preventive Countermeasures in Unavoidable Drug-Induced Risk Situations

#### 3.3.1. Kidney Injury

To prevent renal impairment [32], IPM measures are as follows: 1. Fine-tuning the dosage of all medications, according to the current renal function and pharmacokinetic DDIs, not least with antibiotics. 2. Optimizing blood pressure. 3. The targeted treatment of bradycardia, tachycardia, and arrhythmias. 4. Preventing hypohydration and dehydration. 5. Avoiding single and cumulative nephrotoxic risks from direct drug effects, such as, for example, from NSAIDs monoadministered or even coadministered with ACE inhibitors or sartans and diuretics. 6. The avoidance of single and cumulative indirect nephrotoxic risks from ADRs, such as statins, PPIs, and pharmacodynamic DDIs. 7. The early treatment of bacterial urinary tract infections. 8. The compensation of electrolyte- and acid-base imbalances by timely targeted withdrawal of potentiating drugs and, if compatible with respiratory capacity, the use of bicarbonate. 9. The adherence to standard operating procedures for preventive measures in the administration of contrast media. 

#### 3.3.2. QTc Prolongation

Preventive countermeasures to the often unavoidable risk of QTc prolongation are maintenance of serum potassium and magnesium in the high normal range and exclusion of acidosis. Serum magnesium must be monitored during CsA and TAC because of the risk of hypomagnesemia. This can be fatal, e.g., in children, leading to seizures besides the risk of QTc prolongation and torsades des pointes with life-threatening arrhythmias.

#### 3.3.3. The Differential Diagnosis and Follow-up of Cytomegalovirus Infection (CMV)

For the earliest detection of CMV, lymphocyte subpopulation analysis for CD4/CD8 inversion can be a helpful tool, in addition to the standard viral diagnostic measures, such as PCR, etc. in the differential diagnosis of rejection and GvHD [119], and in the assessment of the therapeutic effect in the course of initiated antiviral treatment. With the additional use of this tool and the early initiation of virustatic therapy, we were able to rule out any CMV-related acute or long-term kidney transplant injuries in the pre-valganciclovir prophylaxis era as early as 1992, resulting in a consecutive two-year transplant survival rate of 100% [119,120,121]. It remains to be seen whether valganciclovir prophylaxis might be more harmful in terms of its nephrotoxic potency than its prophylactic indication. And its dose adjustment to the renal function must be considered. This must be consistently acknowledged by the treating physicians in the transplant outpatient setting as well.

#### 3.3.4. Hypogammaglobulinemia

Monitoring for hypogammaglobulinemia as a potential ADR associated with MMF [76], and unfortunately in patients already at increased risk for infectious diseases due to their immunosuppression, should be a standard measure in the transplant patient on MMF. In case of manifested hypogammaglobulinemia, particularly in the context of concomitant infections, immunoglobulin substitution is recommended, and MMF should be discontinued or, e.g., cortisone-bridged to overcome the infection.

#### 3.3.5. Risks in Analgesics and Sedatives

Note that CsA, TAC, SIR, and EVR exposure is reduced by metamizole (dipyrone), which is a CYP3A4 inducer [68]. This requires dose enhancement and TDM of these immunosuppressants, which is often neglected or unfamiliar to treating physicians. The inducing effect can last up to 5 days after the discontinuation of metamizole. 

NSAIDs must be avoided in the context of CsA and TAC for risk of severe cumulative nephrotoxic ADR.

Paracetamol (acetaminophen (APAP)), requires caution in hepatic and renal impairment, and is contraindicated in severe liver disease. It requires strict adherence to the dosage according to the prescribing information [85,86]. Avoid intake periods of more than 3 days. Intoxications with paracetamol (APAP) are the second most common cause of liver transplants worldwide [87].

For a wide range of sedatives, e.g., midazolam and lorazepam, also for antipsychotics, such as quetiapine, it is important to know that they are metabolized by CYP4A4, which is inhibited by, e.g., CsA [122], azoles, most macrolides, or amiodarone, with the risk of severe ADRs manifestations, such as myelosuppression in HSCT. The same applies to fentanyl, with the risk of fatal toxicity. Furthermore, because of the risk of severe serotonergic syndrome, it is important to know that the coadministration of SSRI/SNRIs with fentanyl or other serotonergic must always be avoided. The serotonin syndrome may include mental-status changes similar to delirium (e.g., agitation, hallucinations, coma), autonomic instability (e.g., tachycardia, labile blood pressure, hyperthermia), neuromuscular abnormalities (e.g., hyperreflexia, incoordination, rigidity), and/or gastrointestinal symptoms (e.g., nausea, vomiting, diarrhea). Serotonergic opioids are the phenylpiperidine opioids fentanyl, methadone, pethidine, and tramadol as well as morphine analogues oxycodone and codeine [56,123]. This does not apply to non-serotonergic morphine or hydromorphone, which may be used as alternatives. 

#### 3.3.6. Life-Threatening Infectious Diseases—Sepsis

The manifestation of severe infectious diseases after transplantation and in the long-term follow-up requires an adequate professional revision of the intensity of the concomitant immunosuppression. Always in consultation with the transplant center, a transient partial reduction or, e.g., bridging with increased cortisone for the temporary withdrawal of CNIs or MMF may save lives.

#### 3.3.7. Surgical Interventions—Wound Healing

In addition to the aforementioned reassessment of the intensity of concomitant immunosuppression required, surgeons in disciplines outside transplantation and unfamiliar with the ADRs of CNIs and mTORIs should be aware that SIR is typically associated with impaired surgical site wound healing. Rates of fluid collections, superficial wound infections, and incisional hernias were significantly higher in SIR patients (47%) when compared to TAC patients (8%), even after adjustment for covariates [4,124]. These risks must be contributed to in any further surgical procedures a transplant patient may undergo, and SIR pausing and bridging with other immunosuppressants needs to be reconsulted with the patient’s transplant center.

#### 3.3.8. Rhabdomyolysis—Statins

Statin drug interactions differ to CsA and TAC. CsA increases the exposure of the HMG-CoA reductase inhibitors simvastatin, atorvastatin, and lovastatin and/or their pharmacologically active metabolites via the inhibition of intestinal and hepatic CYP 3A4 and P-glycoprotein (P-gp). This is less pronounced in TAC, although there are reports of rhabdomyolysis in TAC with simvastatin/fibrate. The same has been observed with SIR. In addition, the active beta-hydroxyacid form of simvastatin, simvastatin acid, atorvastatin, and their metabolites are substrates of the organic anion-transporting polypeptide protein (OATP), which is also inhibited by CsA. This results in an increased risk of musculoskeletal toxicity and rhabdomyolysis. The concomitant use of simvastatin and CsA is considered contraindicated. Since fluvastatin and pravastatin are not extensively metabolized by CYP3A4, these are alternative agents in patients on CsA. However, it is recommended that these statins as well be started at low doses and titrated cautiously, and patients should be advised to report promptly any unexplained muscle pain, tenderness, or weakness, especially if accompanied by fever, malaise, and/or dark urine. Markedly elevated CK requires withdrawal of the statin. Preferentially, pravastatin at its lowest dose or fluvastatin should be used in any transplantation with concomitant CNIs, especially with CsA [43,59,91,96,100,101,102,122,125].

The risk is synergistically amplified by the very frequent concomitant use of calcium channel blockers, which inhibit both CNI and statin metabolism in the hierarchy described.

#### 3.3.9. Calcium Channel Blockers—DDI-Grading

CsA and TAC are associated with an increased prevalence of hypertension in kidney transplant recipients, partly through renal vasoconstriction and enhancement of tubular reabsorption. In addition to their risks for kidney transplantation, a 4-fold increased risk of death due to end-stage renal disease has been documented in non-kidney organ transplant patients associated with CsA-induced nephrotoxicity. The beneficial effect of calcium channel blockers (CCBs) is to counteract these ADRs after transplantation. A pathophysiologic animal study revealed inflammatory, oxidative, and fibrotic pathways in CsA-induced renal dysfunction, inducing proteinuria and elevations in serum creatinine and blood urea nitrogen, mesangial expansion, increases in glomerular and tubular type IV collagen expression, and increases in the glomerulosclerosis and tubulointerstitial fibrosis indices are significantly attenuated up to reversal with the coadministration of CCBs [126]. However, among the different agents available, their varying inhibitory effects on CNI metabolism should be respected. The coadministration of amlodipine with CsA may increase plasma/blood concentrations and the risk of ADRs of both drugs. In the coadministration of amlodipine with TAC, the AUC of TAC may increase by 2.3-fold. TDM of TAC and its dose reduction is important when amlodipine [127] is coadministered. Felodipine can increase the exposure of TAC. With simultaneous use, the TDM of TAC is necessary for the dose reduction of TAC. Felodipine has only a small effect on the blood levels of CsA. But CsA may significantly increase serum felodipine concentrations via the inhibition of CYP3A4 first pass metabolism [128]. Since amlodipine undergoes less first-pass metabolism, it may be considered an alternative. For Lercanidipine, the DDI risk is more pronounced, therefore lercanidipine with TAC should be avoided. The simultaneous administration of lercanidipine and CsA led to a 3-fold increase in lercanidipine plasma levels and a 21% increase in the AUC of CsA. CsA and lercanidipine must not be used together [129]. This risk is less pronounced when given CsA three hours after lercanidipine. 

#### 3.3.10. Diarrhea

With many posttransplant drugs, such as with posaconazole, letermovir, etc., the risk of diarrhea manifestation as a potential ADR must always be taken into account. This is in parallel of high relevance in the differential diagnosis and symptomatic assessment of the course and treatment effect of CMV, rejection, GvHD, *Clostridium difficile* infection, etc.

#### 3.3.11. Loperamide-Induced Cardiotoxicity

Loperamide is a drug that may be added to the transplant patient’s polypharmacy for temporary clinical occasions or on demand. Inhibitors of CYP2C8 or CYP3A4 (TAC less compared to CsA [122]) or the P-gp efflux transporter, such as CsA or TAC, often synergistically with other drugs, may increase the exposure of loperamide in plasma and the central nervous system through the increased systemic exposure of loperamide, and inhibition of P-gp in the blood–brain barrier enhances loperamide entry into the CNS. This may result in enhanced opioid and ADRs. The high plasma levels of loperamide have been associated with serious and fatal cardiac ADRs through QTc prolongation, including arrhythmias, syncope, and cardiac arrest. Concomitant azoles or macrolides or various calcium channel blockers cumulatively increase the loperamide exposure and ADRs furthermore. Patients should be clinically monitored and be instructed to be alert and to consult medical professionals regarding any symptoms of torsade de pointes, such as irregular heartbeat, shortness of breath, dizziness, lightheadedness, fainting, palpitations, or syncope. It was noted that many of the documented loperamide-induced cardiotoxicities required electrical pacing/cardioversion, because the standard antiarrhythmic therapy remained ineffective [130].

#### 3.3.12. Acute Liver Dysfunction

For all patients taking CNIs or mTORIs, as well as other drugs, such as amiodarone, azole antifungals, antivirals, DOACs, etc., liver function monitoring is recommended at individualized, regular intervals, depending on the patient’s condition. Impaired liver function and decreased CYPA4 metabolism, e.g., increase CSA, TAC exposure with subsequent increased nephrotoxicity and the enhanced risk of infections, including CMV. The earliest dose reduction of CNIs and mTORIs and close TDM is essential.

#### 3.3.13. Exchanging CSA or TAC within Different Formulations

Any exchange in CNIs and mTORIs, either in the mode of application or the trading mark, requires close TDM for adequate dose adaption.

#### 3.3.14. Attention Letermovir Metabolism; Letermovir with Voriconazole

Unlike TAC, CsA coadministration with letermovir increases the plasma concentrations of letermovir through the CsA inhibition of the organic anion transporting polypeptide 1B (OATP1B). The oral dose is therefore 240 mg of letermovir once daily with CsA, and 480 mg once daily with TAC [65].

Be aware that the common coadministration with letermovir may decrease the plasma concentrations of drugs that are metabolized by CYP2C9 and/or CYP2C19, e.g., as investigated with voriconazole. Voriconazole peak plasma concentration (Cmax), systemic exposure (AUC), and the concentration at 12 h post-dose (C12hr) decreased by an average of 39%, 44%, and 51%, respectively, when voriconazole 200 mg orally twice daily was coadministered with letermovir 480 mg orally once a day. Dose adjustment may be appropriate with monitoring [65,131].

#### 3.3.15. Monitoring Differentiated ADRs of Immunosuppressants and Integrating Continuous Patient Education

Apart from surgical, patient and graft conditions, the outcome of transplantation depends mainly on the efficacy and optimized dosing of immunosuppressants, which requires strict TDM, but simultaneously on their ADRs. Over the past decades, the various immunosuppressive agents with different combination regimens have been ongoingly investigated to optimize immunosuppression and minimize ADRs. 

Unfortunately, especially in renal transplantation, pre-existing metabolic disorders often require polypharmacy before transplantation already. Posttransplant immunosuppressants enhance this problem, e.g., hypertension, new-onset diabetes after transplantation (NODAT) with cortisone and CNIs, more pronounced with TAC compared to CsA, and hyperlipidemia, more prominent with CsA than with TAC, or hyperlipidemia with mTORIs [132,133]. 

MMF is inert in this context, but given the overall immunosuppressive load, the risk of infection and hypogammaglobulinemia is increased here. And manifest CMV disease itself, as well as HHV6 or BK virus, severely affect graft function. Consecutive antiviral therapy with its own nephrotoxic potential completes this vicious circle. Intravenous immunoglobulin has been reported as a preventive strategy against BK virus viremia and BKV-associated nephropathy [134].

Therefore, the pre- and posttransplant status of each patient, along with its individual risk factors and transplant course, should be clarified as precisely as possible to tailor the immunosuppressive regimen. According to the ADRs of the posttransplant immunosuppressants, the main cause of death after organ transplantation is a cardiovascular event such as stroke or myocardial infarction with a functioning graft, also frequently in HSCT [135]. The inevitable polypharmacy to treat the broad spectrum of cardiovascular risk factors makes IPM all the more necessary, since deprescribing is not possible in this context. 

These ADRs and others that impact long-term survival require additional strategic follow-up, including ongoing patient education. As learned from the acute and long-term care, most transplant patients are eager and grateful to be informed about the risks and how to maintain and preserve the graft with their own abilities and engagement. For active patient involvement in the posttransplant period, they need to be educated on remarkable and vulnerable aspects: 1. The optimization of blood pressure and the regular follow-up of blood glucose levels. 2. The risk of increased drug nephrotoxicity as from CNIs with hypohydration, respecting the individual condition and fluid balance. 3. The adherence to consistent immunosuppressive TDM. 4. The communication of any additional medications and supplements prior to their use. 5. The avoidance of any NSAIDs with CNIs. 6. Observations for urinary tract infections or even the initial signs of developing Fournier’s gangrene, as with SGLT2 inhibitors, such as dapagliflozin and empagliflozin. 7. Liver injury caused by unregulated alcohol, food, or dietary supplements, and herbal products, e.g., turmeric-associated liver damage (active ingredient curcumin) as a growing problem [136], or by drugs like paracetamol (acetaminophen). In addition to patient information, it remains essential that clinicians consistently ask for supplements as part of medication reconciliation. The patient always must avoid grapefruit juice in terms of nephrotoxicity via CNI-elevation and St. John’s wort with rejection risk via CNI-decrease. The severe increase in TAC exposure observed with cannabidiol [118] via CYP3A4 inhibition may be indicative of a similar risk for CsA, SIR, EVR and many other drugs, e.g. most statins, calcium channel blockers, sedatives, even some opioids, resulting in serious risk situations. Further research needs to be done here. 8. Regular blood controls to exclude hypomagnesemia caused by CNIs, in addition to hypokalemia, particularly in the setting of prolonged QTc after transplantation, as it is highly prevalent, e.g., in renal transplant patients receiving various classes of immunosuppressive drugs [137], or even cumulative long QTc risks requiring intermittent ECG monitoring for this risk marker of serious arrhythmias and sudden death. 9. To report resorption concerns such as by vomiting or diarrhea from the very beginning. 10. All patients, especially the HSCT patients, must avoid additional risks of myelotoxic ADRs, paricularly in the early phase, and should be monitored and advised to maintain regular, but not overdosed, folic acid and vitamin B12 blood levels, which may be reduced by drugs such as phenytoin, methotrexate, cotrimoxazole, with different interference of folic and folinic acid on the antimicrobial effects to be considered, or vitamin B12 deficiency through proton pump inhibitors, and metformin, respectively. 11. Severe night sweats may be an early indicator of CMV disease and need to be reported immediately [120,121]. Patient empowerment through appropriate information can be a valuable contribution to risk prevention.

In terms of further minimizing cardiovascular risks and events after transplantation, the patient should ideally be instructed and guided to maintain or regain a normal BMI through a healthy lifestyle and individualized training.

## 4. Discussion

This briefing provides an overdue all-in-one drug safety tool to securely navigate through the individual high-risk context of posttransplant medication. The systematic, methodical intervention to prevent iatrogenic drug-induced injury covers ADRs, DDIs, and contraindications with consistent reference to immunosuppressants. The tabular extracts of the clinically relevant aspects of posttransplant polypharmacy will be a welcome tool for accurate and appropriate medication review according to the successfully implemented IPM. It saves a lot of time in daily routine as it is a breakdown of thousands of SmPC pages. The focus on contraindications, ADRs, and drug-specific warnings, in addition to the usual DDIs, expands the scope of the commonly available DDI tables. Consideration of each of these medication scores in the context of the patient’s condition has already been shown to be impressively associated with successful prevention of the risks of polypharmacy, resulting in highly significant prevention of renal impairment (100%), delirium (decrease 92%; 10-fold reduction), and falls (83%), as well as further improvements after IPM implementation in multimorbid patients within other polypharmacy settings [32,138,139]. The briefing provided for colleagues inside and outside transplantation, who regularly or occasionally care for transplant patients, includes both the extracts of the predominant risks of concerning drugs in posttransplant coadministration and the own implemented preventive countermeasures for critical patient conditions successfully applied within 10 years of IPM and 21 years of TDM in immunosuppressants posttransplant. Since no such comprehensive information is available in the literature, these two items are intended to be a helpful resource toolset in the setting of the complex posttransplant polypharmacy.

Despite decades of continuous progress in organ and stem cell transplantation, the improvement of graft and patient outcomes does not seem to have been further optimized in recent years, e.g., in kidney transplantation [140]. Although the immunosuppressive regimen has been continuously improved according to expertise and research, the problem of polypharmacy, which is constantly increasing, has been largely neglected, with the exception of DDIs for immunosuppressants. Focusing on graft and patient risk through more accurate and comprehensive medication scores could further improve transplant outcomes and quality of life. Polypharmacy has been shown to be associated with poorer quality of life in patients after successful kidney transplantation, and multivariate regression analysis showed that the number of medications independently affected physical function, pain, and social function subscales [141]. It is already known from the pretransplant focus that CKD stage G4/G5 patients and patients on renal replacement therapy have a medication burden far beyond that of the general population, also due to a high burden of comorbidities besides CKD. The authors indicate that a critical approach to medication prescribing could be a first step towards more appropriate medication use [142]. What really happens to ensure this? The issue should be of analogously high importance after a successful transplantation. Analysis of the impact of polypharmacy prior to allogeneic HSCT in older adults demonstrates the relevance of pre-HSCT polypharmacy, potentially inappropriate medication and DDIs as important prognostic factors for inferior post-HSCT outcomes or increased hospital length of stay, respectively. These results should support routine pre-HSCT medication review by physicians and pharmacists with the additional goal of appropriate deprescribing [143]. However, the mandatory treatment of the manifest metabolic ADRs of immunosuppressive drugs must be maintained on an optimized level all times.

The provided systematic IPM method adapted to transplantation for the prevention of iatrogenic drug-induced injury has been evaluated with very successful results in other critical polypharmacy settings and has been tested with positive results for its applicability by clinical and community pharmacists. The toolset with the overview and briefing on drug safety/drug therapy safety in transplantation based on real-world IPM enables proactive mitigation of polypharmacy hazards for both the patient and the graft. As a result from daily insights the selection of drugs intentionally addresses frequent patient’s chronic comorbidities, acute illnesses and probably complications in the transplantation course. All of these even abruptly changing intraindividual clinical situations result in varying drug elimination capacities, which are further affected by the additional drugs and their own add-on DDIs and ADRs. The constant focus on drug-induced risks in transplantation and patients on polypharmacy with IPM over 10 years results in a broad real-world expertise of >60,800 medication analyses. To sensitize the transplant team and all other attending physicians on the long-term base for the critical spectrum of the identified predominant risks from DDIs, ADRs, overdosage and contraindications as depicted primarily from the drugs’ SmPCs, interaction and dosing checks, and literature research (medication scores) in context with the very individual patient condition (patient scores) and the therapeutic drug monitoring of immunosuppressants is the aim of this briefing. For best practice in transplantation to improve outcomes, iatrogenic drug-induced injury has to be prevented from the very earliest stage, avoiding any harm to the susceptible transplant organ and the vulnerable, highly immunosuppressed patient with his already preexisting risks and comorbidities. 

This is the first briefing overview to consider frequently coadministered polypharmacy in early and long-term transplantation phases. Since the application of the reproducible IPM method only relies on a digital patient record, it does not necessarily require on-site capacities or competencies but can also be performed by instructed physicians or pharmacists from outside. The briefing on the real-world concomitant polypharmacy risks in hospitalized and outpatients experienced in transplantation IPM over 10 years at the University Hospital Halle is to ensure drug therapy safety for further amelioration of acute and long-term graft and patient outcomes with the respect to each very individual patient condition. 

The major challenges affecting graft and patient outcomes in solid organ and HSCT immunosuppression are to reduce toxicity while maintaining efficacy and preventing any iatrogenic drug injury. This requires that the risks of over- and underdosing for each individual patient and transplant condition be eliminated as early as possible during the acute and, in solid organ transplantation, the long-term posttransplant phases. However, these risks are not only due to the immunosuppressive regimen, but are also strongly influenced by concomitant polypharmacy. As shown in the table, each drug must be evaluated for its own toxicity in relation to the transplant and the individual patient’s condition, always in the context of the entire medication list. The tabulated risks from ADRs, DDIs, contraindications and additional drug-specific aspects reflect these broad aspects to be considered in clinical practice. To date, there is no comparable adequate overview of concomitantly administered medications within the polypharmacy of transplant patients. Increased morbidity and unexpected outcomes are consequences of DDIs in allogenic HSCT [144]. Mainly lists of DDIs are available, and from the worst end of drug-related injuries up to organ failures, graft loss and patient death, there are numerous case reports, extended by reviews and supplemented lists of drugs, e.g., associated with failure of liver transplantation [12,145,146,147] and furthermore in the outpatient setting DDIs in kidney transplantation [148]. Since the transplant patient often suffers from multimorbidity with unavoidable polypharmacy being already on pre-existing chronic kidney disease (CKD), as it may also be the result in a renal transplant, a rigorous focus on the exclusion of any iatrogenic, additional drug-induced decline in renal function is essential in the context of the CNI’s own nephrotoxicity and the vulnerability of the kidney transplant. CsA and TAC make it all the more important to focus on optimizing this multimedication process, particularly for enhanced risks of ADRs and DDIs with the pre-impaired drug degrading or eliminating organs [149] and further afflicted vulnerable transplant patients. 

It is of great concern that there remain transplant patients who are coadministered NSAIDs with all their own associated ADRs and pharmacodynamically increased cumulative risk of nephrotoxicity from DDI with CNIs [150,151,152]. We even find patients on triple whammy, the concomitant use of diuretics and angiotensin-converting enzyme (ACE) inhibitors or angiotensin receptor blockers (sartans) with NSAIDs, which per se is associated with high risk of acute renal failure [153], independent of the type of organ transplant or HSCT and further enhanced by CsA or TAC. Diuretics-associated kidney injury leading to AKI has been documented with pathological details showing vacuolar degeneration of tubular epithelial cells as a common lesion induced mainly by loop diuretics, with age being a predictive factor for incomplete recovery and all-cause mortality, and changes being more severe at high doses [154]. The coadministration of loop diuretics and HCT, preferably xipamide, as IPM-targeted in individual cases to benefit from transient sequential nephron blockade, is also known to further improve diastolic function in patients with resistant hypertension [155,156]. It allows the dose of the single diuretic to be reduced but should be time-limited and requires parallel serum electrolyte monitoring.

Study results remain controversial regarding the effects of allopurinol on CKD. Hyperuricemia, linked with inflammation but also with progression of renal and cardiovascular disease, has been effectively treated with allopurinol, with a beneficial effect on all these conditions [157]. A recently published large RCT in the United Kingdom in patients aged ≥60 years showed no difference in the primary outcome of nonfatal myocardial infarction, nonfatal stroke, or cardiovascular death between participants randomized to allopurinol therapy and usual care [158]. However, treated patients had relatively low mean serum uric acid concentrations at baseline, and the association between allopurinol-induced changes in serum uric acid concentrations and outcomes was not confirmed [159]. From own IPM findings in the serum uric acid-lowering drugs, when indicated in patients with symptomatic hyperuricemia, allopurinol often failed to be dose adjusted in advanced CKD. Similarly for febuxostat, its exposure is known to be increased 2–4-fold in severe renal disease with eGFR < 30 mL/min, requiring withdrawal due to insufficient safety yet, and it is not recommended in organ transplant patients or in patients with ischemic heart disease or decompensated heart failure [160], aspects that are also largely ignored according to our IPM analyses. Despite broad experimental and epidemiologic evidence for hyperuricemia as a risk factor for CKD, the overall evidence for the therapeutic effect of allopurinol is insufficient [161]. The KDIGO 2012 Clinical Practice Guideline for the Evaluation and Management of Chronic Kidney Disease states: “Hyperuricemia 3.1.20: There is insufficient evidence to support or refute the use of agents to lower serum uric acid concentrations in people with CKD and either symptomatic or asymptomatic hyperuricemia to delay progression of CKD. (Not graded)” [162] (p. 10).

In addition, there are often serious acute risks from overdosing, especially with antibiotics [32], which are further increased without adequate parallel hydration. Also statins in high doses or by DDI-enhanced exposure, which could affect the kidney graft function in the acute and long-term survey [163,164,165,166,167], are most often observed not to be dose adjusted. Respecting the DDI with TAC and CsA, pravastatin should be preferred, although dose adjustments are required. The same is true for proton pump inhibitors (PPIs), which are mostly overdosed; especially a prophylactic dose of pantoprazole should not exceed 20 mg per day. However, the results on the renal risks of PPIs are controversial and have been abandoned, for example, in renal transplant patients [81]. Although there is evidence that PPI use is associated with an increased risk of CKD in terms of incident CKD, progression of CKD and renal failure [82], we often find non-indicated long-term prescriptions. A graded increase in risk with higher doses and longer duration of PPI therapy has been described [82]. Associations between PPI use and the risk of acute interstitial nephritis and acute kidney injury (AKI) have been reported, particularly among hospitalized patients. A direct pathway to indolent chronic kidney injury has been suggested [82]. The demonstrated temporal association between exposure to PPIs and the occurrence of AIN may strengthen a causal relationship [83]. As PPI use has been associated with an increased risk of chronic kidney injury even in the absence of AKI, relying on AKI as a precautionary sign may not be sufficient to reduce the risk of CKD in PPI users [84].

A widely cited review of ADRs from 25 years ago recognized this common clinical problem and stated that clinician vigilance in detecting, diagnosing, and reporting ADRs is important for ongoing drug safety monitoring [168]. So do we really consistently monitor for the elimination of serious ADRs? In the posttransplant polypharmacy setting, specific pharmacovigilance for each drug in this context presents an even more complex challenge, as the problem may be compounded by increased drug exposure due to pharmacokinetic DDIs or enhanced effects related to cumulative pharmacodynamic drug effects and ADRs. Without knowledge of the major ADRs and DDIs of each drug, the problem becomes almost insurmountable. Another intensive review of the factors influencing the development of ADRs revealed that various risk factors such as age, renal and hepatic function, drug dose and frequency play a key role [169]. The additional susceptibility of the immunosuppressed and often multimorbid transplant patient may increase the potential for influence.

For the care of transplant patients, there are lists of DDIs, some of which have been taken from books, such as those on DDIs in the drug therapy of heart transplant recipients [170,171]. The table presented here prefers the drug-specific SmPC in its most recent and more complete version, especially since it also includes the ADRs and contraindications of the drugs. This is of analogous additional importance. The pioneering teams of Calne in kidney transplantation [172] and Starzl in early liver transplantation were already struggling with the dose-dependent severe nephrotoxicity of CsA, and Starzl advocated some dose reduction to overcome this effect without liver transplant rejection [173]. Despite decades later with the availability of other immunosuppressants, these CNI risks of nephrotoxicity and neurotoxicity remain unchanged and often go unrecognized in patients without adequate prevention and close monitoring. In the long term, this increases chronic graft failure and may be partly responsible for the long-term outcome of renal allografts worldwide. In this context, the coadministration of nephrotoxic agents that should be avoided, such as NSAIDs, remains a concern for the treating physician. Patients need to be educated about these risks in the outpatient setting. Chronic CsA nephrotoxicity is the second most important diagnosis responsible for late graft failure. Clinicopathological CSA-associated arteriolopathy (CAA) is a well-known lesion of chronic CSA nephrotoxicity [174]. It was the Mihatsch group, among others, that consistently focused on the pathological findings over decades. In addition, focal segmental glomerulosclerosis (FGS) lesions accompanying CAA have been considered as CSA-associated glomerulopathy. Importantly, intraindividually variable CsA predose was evaluated as an independent predictor of chronic rejection and graft loss. Thus, the authors emphasize the need for therapeutic drug monitoring, ideally 2 h (C2) CsA measurements, but also for patient’s adherence instructions and for controlling consistent CsA delivery using new generic formulations [175]. Intraindividual variability becomes apparent, particularly abruptly from the onset of pharmacokinetic DDIs with changing concomitant medications requiring earliest adjustment and close TDM.

All these data from different perspectives, such as the timing and frequency of TDM, the knowledge of drug-associated pathological injuries, the variants in different generic drug formulations, the informed patient, and, of major importance, the broad spectrum of ADRs, DDIs, and contraindications underline that the more the treating physician is sensitized, the greater the benefit for the graft and the patient’s outcome. 

## 5. Strengths and Weaknesses

IPM requires a holistic clinical view of the patient’s entire medication regimen. The educational background of the designing and performing IPM internist with advanced education in clinical pharmacology also covers a broad spectrum of expertise and successful engagement in improving outcomes in clinical transplantation [119,120,121,176,177,178,179,180,181,182,183,184,185] and the qualification as Distinguished Educator of the Transplantation Society Academy, thus ensuring experience in a broad professional transplantation context. In addition to 21 years of daily experience in TDM of immunosuppressants in transplantation, this allows a highly patient-individualized focus for a comprehensive evaluation. The greatest achievement of her former kidney transplant team at the University of Bonn was a two-year consecutive survival rate of 100% for kidney transplants already in the 1992 [121]. The tabulated risks are mainly extracted from updated SmPCs, and therefore depend on the author’s own experience in terms of what is covered and what is excluded. It is important to outline that the task of reducing SmPCs from 10 up to even more than 100 pages for one drug means that, for each drug, there is a deliberate exclusion of other relevant aspects, such as pregnancy, lactation, pediatric aspects, hypersensitivity, intolerance related to congenital disorders, among others. Therefore, the table is not a complete replacement for the SmPCs. The list of drugs is not exhaustive with respect to contemporaneously coadministered drugs in transplantation. In particular, the DDIs referenced in the SmPCs always remain incomplete because they are never extensively studied in vitro and only rudimentarily, if at all, in vivo. And most strikingly, there are even approved drugs that still lack degradation information, the indispensable prerequisite for the analysis of pharmacokinetic DDIs. In very rare and adequately justified off-label cases to overcome life-threatening conditions of patients, individual drug indications or contraindications from the SmPCs are intentionally not adhered to. Also rarely, the spectrum of use and dosing within the limits of impaired renal function may change in the course of post-marketing experience with a drug. These upcoming cases and highly individualized dosing cannot be covered in this overview. With the exception of grapefruit juice, St. John’s wort, and cannabis, the tabularized medicinal items presented do not cover foods and substances related to complementary and alternative medicine (CAM), such as dietary supplements, herbs, and other manufactured ingredients, although they have been shown to have a further relevant impact, especially in outpatients, as in cancer therapy, with a likelihood of interactions as high as 37% in the case of CAM supplements, and 29% of all patients for foods [186,187]. There is no reference to CYP3A4, CYP3A5, or MDR-1 genotype metabolism for additional pharmacokinetic genotype-based individualized dosing aspects of, e.g., CSA or TAC in genetic polymorphism that also affects outcomes [188].

## 6. Conclusions and Outlook

To improve drug safety and patient and transplant outcomes in the context of polypharmacy requires the implementation of standardized methods that refer to all relevant risks, resulting from a single drug administration as well as from its coadministration within a broad spectrum of further medications and the individual patient condition itself. Since the range of ADRs and DDIs is immense and the study of large numbers of SmPC pages for each drug is almost unmanageable in daily routine, the provided overview could be a helpful tool in practice, other than the simultaneously available computerized DDI checks and DDI tables, which do not address specifically ADRs and individual patient-condition-related contraindications or individual requirements in multimorbidity and polypharmacy. In addition to improving long-term graft survival, the presented briefing also addresses the recently documented decline in 5-year kidney transplant outcomes, indicating a remaining unmet demand for innovation in the early transplant phase as well [140].

The outlook is to integrate the comprehensive IPM findings, such as the provided overview, into a forthcoming digital IPM support system that is standardized and compatible with the use of the transplant patient’s electronic health record and aims to be accessible and understandable for all healthcare professionals, the patients themselves, and their families. The entirely computerized identification of polypharmacy risks through the defined and implemented medication scores to the individual patient and his/her most currently updated health status, organ functions, and comedications based on the electronic health record, as performed by the IPM, will be of great benefit not only to the transplant field, as for serious risks observed in the contemporary real-world polypharmacy, the highly preventive effect of IPM has been documented by the first published IPM results [32,138,139]. There is no database integrated with an electronic health record system to perform this IPM in a holistic, elaborate manner, including the defined encompassing patient and medication scores. The far-reaching concerning iatrogenic medication risks arising from today’s increasing polypharmacy require earliest elimination. 

The intentionally comprehensive IPM concept for medication safety meets the demands of the global challenges continually being addressed by the WHO: “A comprehensive medication review is a multidisciplinary activity whereby the risks and benefits of each medicine are considered … It optimizes the use of medicines for each individual patient…Polypharmacy can put the patient at risk of adverse drug events and drug interactions when not used appropriately” [189] (p. 7).

This second paper, following the first IPM/TDM design paper, is to provide the additional corresponding practical toolset to cover the enormous challenges of the defined medication scores that require awareness and briefing. It is intended as an informative all-in-one resource for transplantologists and subsequent attending physicians, who often come from different disciplines and are unfamiliar with the risks of polypharmacy.

Our human and professional ethics, the donor, the transplant, and the recipient, as well as the shortage of organs, obligate us to continue to promote optimal patient and transplant care. 

## Figures and Tables

**Table 1:** IPM medication scores extracts from the SmPCs of 65 applied drugs (alphabetical order) in transplantation with selected risks (not graded) to be recognized from ADRs (from reported placebo-controlled studies and from post-marketing experience), pharmacokinetic and pharmacodynamic DDIs, contraindications (CI), and dosing aspects in contemporary polypharmacy in the context of solid organ transplantation and HSCT, with reference to CNIs and mTORIs, cortisone, and MMF.

Medication Risks to Be Recognized in Solid Organ and Hematopoietic Stem Cell Transplantation				
Drug	ADR *	DDI	CI **	Other Aspects
**Aciclovir **[33]	headache, dizziness; nausea, vomiting, diarrhea, abdominal pains; fever, fatigue; symptoms of overdose include agitation, coma, seizures, lethargy, and precipitation in renal tubules, more common in patients given high doses without monitoring of fluid and electrolyte balancing or reduced kidney function	eliminated primarily unchanged via active renal tubular secretion, any concurrent drug competing with this mechanism may increase aciclovir plasma concentrations, probenecid and cimetidine increase the AUC of aciclovir by this mechanism, and reduce aciclovir renal clearance; similarly **increases in the plasma AUCs of aciclovir, mycophenolate mofetil, and mycophenolate acid when coadministered**; the **risk of renal impairment** is increased by the concomitant use of other nephrotoxic drugs; may increase serum theophylline levels; **risk or severity of nephrotoxicity can be increased with CsA, TAC;** excretion of aciclovir can be decreased when combined with methotrexate		with caution in patients with underlying neurological abnormalities, severe hepatic or electrolyte abnormalities or significant hypoxia; maintain adequate hydration; **dosage reduction may be required in elderly; renal dose adjustment**
**Allopurinol **[34]	should be withdrawn immediately if a skin rash or other evidence of sensitivity occurs, as this could result in more serious hypersensitivity reactions (chronic renal impairment and concomitant diuretic use, in particular thiazides, may increase the risk of hypersensitivity reactions, including SJS/TEN, HLA-B*5801 allele has been shown to be associated with the risk of developing allopurinol-related hypersensitivity syndrome and SJS/TEN, up to 20% prevalence in the Han Chinese population); xanthine deposition in the urinary track, minimized by adequate hydration; increased TSH values; very rare reports have been received of thrombocytopenia, agranulocytosis, and aplastic anemia, particularly in individuals with impaired renal or hepatic function	**CsA level may be increased;** 6-mercaptopurine or azathioprine at only one-quarter of the usual dose because inhibition of xanthine oxidase will prolong their activity; vidarabine is increased, enhancing toxic effects; oxipurinol, the major metabolite of allopurinol and itself therapeutically active, is excreted by the kidney in a similar way to urate, hence, drugs with uricosuric activity, such as probenecid or large doses of salicylate, may accelerate the excretion of oxipurinol, which may decrease the therapeutic activity of allopurinol; with chlorpropamide in poor renal function, the increased risk of prolonged hypoglycemic activity because allopurinol and chlorpropamide may compete for excretion in the renal tubule; increased effect of warfarin and other coumarin anticoagulants; hepatic oxidation of phenytoin inhibited; metabolism of theophylline inhibited; increase in the frequency of skin rashes among patients receiving ampicillin or amoxicillin concurrently, it is recommended that in patients receiving allopurinol, an alternative to ampicillin or amoxicillin is used where available; with cyclophosphamide, doxorubicin, bleomycin, procarbazine, mechlorethamine, enhanced bone marrow suppression is likely; didanosine level elevated, combination not recommended; with furosemide, increased serum urate and plasma oxypurinol concentrations; increased risk of hypersensitivity with diuretics, in particular thiazides, and ACE inhibitors, especially in renal impairments; with cytostatics (e.g., cyclophosphamide, doxorubicin, bleomycin, procarbazine, alkyl halogenides), blood dyscrasias more frequently; with aluminum hydroxide concomitantly, allopurinol may have an attenuated effect, there should be an interval of at least 3 h between taking both medicines	**asymptomatic hyperuricemia per se is generally not considered an indication for the use of allopurinol**; should not be started until an acute attack of gout has completely subsided, as further attacks may be precipitated	**reduced doses in patients with renal or hepatic impairment**
**Amiodarone** [35]	bradycardia, conduction disturbances; the pharmacological action of amiodarone induces ECG changes: QTc prolongation (related to prolonged repolarization) with the possible development of U-waves and deformed T-waves; these changes do not reflect toxicity; in the elderly, heart rate may decrease markedly; treatment should be discontinued in case of onset of second or third degree AV block, sino-atrial block, or fascicular block; amiodarone has a low pro-arrhythmic effect, despite QTc interval prolongation, amiodarone exhibits a low torsadogenic activity; hypothyroidism, hyperthyroidism; eye disorders, corneal microdeposits; benign gastrointestinal disorders (nausea, vomiting, dysgeusia), usually occurring with loading dosage and resolving with dose reduction; constipation; hepatobiliary disorders with isolated increases in serum transaminases, acute liver disorders with high serum transaminases and/or jaundice, including hepatic failure, which are sometimes fatal; peripheral sensorimotor neuropathy and/or myopathy, may be severe; extrapyramidal tremor, nightmares, sleep disorders; pulmonary toxicity (sometimes fatal); photosensitivity	**increase in CsA, TAC, SIR, EVR,** simvastatin, atorvastatin, lovastatin, amlodipine, lercanidipine, felodipine, fentanyl, buprenorphine, midazolam, quetiapine, mirtazapine, aprepitant, lidocaine, DOAC; pharmacodynamic DDIs with drugs inducing torsade de pointes or prolonging QTc intervals incl. erythromycin i.v., co-trimoxazole, haloperidol, doxepin, amitriptyline, moxifloxacin; potentially severe complications have been reported in patients undergoing general anesthesia: bradycardia unresponsive to atropine, hypotension, disturbances of conduction, decreased cardiac output; a few cases of adult respiratory distress syndrome, sometimes fatal, most often in the period immediately after surgery, have been observed, a possible interaction with a high oxygen concentration may be implicated; with hepatitis C medicines with or without other medicines that lower the heart rate risk of bradycardia and heart block; concomitant use not recommended with beta-blockers, heart rate lowering calcium channel inhibitors (verapamil, diltiazem), stimulant laxative agents which may cause hypokalemia; increased plasma levels of flecainide, should be reduced accordingly and the patient closely monitored; not to be taken alongside grapefruit juice consumption	thyroid dysfunction; before surgery, the anesthetist should be informed that the patient is taking amiodarone; sinus bradycardia and sino-atrial heart block, in patients with severe conduction disturbances (high grade AV block, bifascicular or trifascicular block) or sinus node disease be used only in conjunction with a pacemaker; before starting, it is recommended to perform an ECG and serum potassium and magnesium measurements; monitoring of ECG is recommended during treatment; may increase the defibrillation threshold and/or pacing threshold in patients with an implantable cardioverter defibrillator or a pacemaker, which may adversely affect the efficacy of the device, regular tests are recommended to ensure the proper function of the device; combination with drugs which may induce torsades de pointes, such as moxifloxacin (cave fluoroquinolones), contraindicated	treatment should be limited; half-life of 50 days (20–100); monitor thyroid function: **Primary Graft Dysfunction (PGD) post-cardiac transplant**: in retrospective studies, amiodarone use in the transplant recipient prior to heart transplant has been associated with an increased risk of PGD (left, right, or biventricular dysfunction occurring within the first 24 h of transplant surgery, for which there is no identifiable secondary cause, may be irreversible), for **patients who are on the heart transplant waiting list**, consideration should be given to **use an alternative antiarrhythmic drug as early as possible before transplantation**
**Amlodipine** [36] DDIs present class effect, also with other dihydropyridine calcium antagonists	oedema; fatigue, asthenia; somnolence, dizziness, headache (especially at the beginning of the treatment); visual disturbance (including diplopia); palpitations; flushing; dyspnea; abdominal pain, nausea, dyspepsia, altered bowel habits (including diarrhea and constipation), ankle swelling, muscle cramp	with strong or moderate CYP3A4 inhibitors (protease inhibitors, azole antifungals, macrolides like erythromycin or clarithromycin, verapamil or diltiazem) may give rise to a significant increase in amlodipine exposure; decreased amlodipine exposure with strong CYP3A4 inducers (e.g., rifampicin, St. John’s wort); **increased blood levels of CsA, TAC, SIR, EVR**; increased risk of angioedema with mTORIs; limit the dose of simvastatin in patients on amlodipine to 20 mg daily	not in severe hypotension, shock, including cardiogenic shock, obstruction of the outflow-tract of the left ventricle (e.g., high grade aortic stenosis), hemodynamically unstable heart failure after acute myocardial infarction	with hepatic impairment, dose selection should be cautious and should start at the lower end of the dosing range; in the elderly, any increase of the dosage should take place with care
**Amphotericin B, AmBisome Liposomal** [37]	renal toxicity, hypokalemia hypomagnesemia, hypocalcemia notably higher with high dose, regular evaluation of electrolytes, renal, hepatic, and hematopoietic function, appropriate potassium supplementation; high portion of sucrose caution in diabetes mellitus; hyperglycemia; headache; tachycardia; hypotension, vasodilatation, flushing; dyspnea; nausea, vomiting, diarrhea, abdominal pain; abnormal liver function tests, hyperbilirubinemia, increased alkaline phosphatase; renal toxicity, increased creatinine, increased blood urea; rigors, pyrexia; chest pain	with other nephrotoxic agents (for example **CsA, TAC**, aminoglycosides, polymyxins, and pentamidine) may enhance the potential for drug-induced renal toxicity; corticosteroids, ACTH, and diuretics (loop and thiazide) may potentiate hypokalemia; antineoplastic agents may enhance the potential for renal toxicity, bronchospasm, and hypotension, and should be given concomitantly with caution	acute pulmonary toxicity given amphotericin B (such as sodium deoxycholate complexes) during or shortly after leukocyte transfusions, recommended that these infusions are separated by as long a period as possible, and pulmonary function should be monitored	if clinically significant reduction in renal function, then dose reduction or treatment interruption; no benefit from the use of flucytosine with AmBisome has been observed, whilst synergy between amphotericin and flucytosine reported, concurrent use may increase the toxicity of flucytosine by possibly increasing its cellular uptake and/or impairing its renal excretion; false elevations of serum phosphate in PHOSm assays
**Apixaban** [38] As an example for DOACs	hemorrhage, eye hemorrhage contusion, epistaxis, hematoma; nausea; gastrointestinal hemorrhage; mouth hemorrhage; rectal hemorrhage, gingival bleeding, hematuria, anemia; YGT, ALT increased; abnormal vaginal hemorrhage, urogenital hemorrhage; contusion	closer monitoring of **increased anticoagulation effects of apixaban whenever a CYP450 3A4 or P-gp inhibitor is added, incl. CsA, TAC;** caution increased bleeding risk with SSRIs/SNRIs, NSAIDs, ASA, and/or P2Y12 inhibitors; following surgery, other platelet aggregation inhibitors are not recommended concomitantly with apixaban; active substances which are not considered strong inhibitors of both CYP3A4 and P-gp, e.g., amiodarone, clarithromycin, diltiazem, fluconazole, naproxen, quinidine, verapamil, are expected to increase the apixaban plasma concentration to a lesser extent	not recommended in concomitant systemic treatment with strong inhibitors of both CYP3A4 and P-gp, such as azole-antimycotics, e.g., ketoconazole, itraconazole, voriconazole, posaconazole, and HIV protease inhibitors, e.g., ritonavir; strong CYP3A4 and P-gp inducers, e.g., rifampicin, phenytoin, carbamazepine, phenobarbital, or St. John’s Wort may lead to a ~50% reduction in apixaban exposure, thus not to be used for the treatment of DVT and treatment of PE; not in active clinically significant bleeding, lesions, or conditions if considered a significant risk factor for major bleeding; not in hepatic diseases associated with coagulopathy and clinically relevant bleeding risk; not in severe hepatic impairment, coagulative disease; caution in elevated ALT/AST > 2 × ULN or total bilirubin ≥ 1.5 × ULN; no concomitant treatment with any other anticoagulant agent; DOACs are not recommended for patients diagnosed with antiphospholipid syndrome; GPIIb/IIIa receptor antagonists, dipyridamole, dextran, sulfinpyrazone, or thrombolytic agents not recommended	caution in conditions with increased risks of hemorrhage, e.g., thrombocytopenia; for the prevention of stroke and systemic embolism in patients with NVAF and serum creatinine ≥ 1.5 mg/dL (133 micromole/L) associated with age ≥ 80 years or body weight ≤ 60 kg, a dose reduction is necessary; in patients with severe renal impairment (creatinine clearance 15–29 mL/min), dose reduction is required (compared to, e.g., rivaroxaban, which already requires dose reduction at creatinine clearance < 50 mL/min); creatinine clearance < 15 mL/min, or in patients undergoing dialysis not recommended; calibrated quantitative anti-Factor Xa assays in acute bleeding risks; antidot adexanet alfa available
**Aprepitant** [39,40]	decreased appetite, headache, hiccups, constipation, dyspepsia, fatigue; increased ALT	several-fold increase in plasma concentrations of aprepitant with active substances that inhibit CYP3A4 activity (e.g., ketoconazole, itraconazole, voriconazole, posaconazole, clarithromycin, telithromycin, nefazodone, and protease inhibitors); strong CYP3A4 inducers (e.g., rifampicin, phenytoin, carbamazepine, phenobarbital) reduce aprepitant efficacy; **transient moderate increase, followed by a mild decrease in the exposure of CSA, TAC, SIR, EVR**; increased exposure of simvastatin, atorvastatin, lovastatin, amlodipine, lercanidipine, felodipine, fentanyl, uprenorphine, midazolam, quetiapine, mirtazapine, ergot alkaloid derivatives, quinidine, and irinotecan; reduced efficacy of hormonal contraceptives during and for 28 days after administration, alternative non-hormonal back-up methods of contraception should be used during treatment with aprepitant and for 2 months following the last dose; oral dexamethasone and methylprednisolone dose should be reduced by approximately 50% when coadministered; during continuous treatment with methylprednisolone, the AUC of methylprednisolone may decrease at later time points within 2 weeks following the initiation of the aprepitant dose, due to the additional inducing effect of aprepitant on CYP3A4; caution potential increase of orally administered chemotherapeutic medicinal products metabolized primarily or partly by CYP3A4 (e.g., etoposide, vinorelbine, ifosfamide); decrease in warfarin, acenocoumarol, tolbutamide, and phenytoin via CYP2C9/CYP3A4 induction, apparent only after the end of a 3-day treatment with aprepitant, the induction is transient with a maximum effect reached 3–5 days after the end of the aprepitant 3-day treatment, the effect is maintained for a few days	no coadministration with pimozide, terfenadine, astemizole, or cisapride, not with St. John’s wort	caution in patients with moderate to severe hepatic impairment
**ASA** [41,42] acetyl salicylic acid	bleeding and hemorrhagic tendency (epistaxis, bleeding gums, purpura, etc.), bleeding risk may persist for 4 to 8 days after discontinuation, increased risk of hemorrhage in the event of surgery, intracranial and gastrointestinal hemorrhage; anaphylactic reactions, asthma, angioedema; rhinitis, dyspnea, bronchospasm; headache, dizziness, sensation of hearing loss, tinnitus, which are usually indicative of an overdose; abdominal pain, dyspepsia, nausea, vomiting; occult or patent gastrointestinal hemorrhage (hematemesis, melaena, etc.), resulting in iron-deficiency anemia, the bleeding risk is dose-dependent, gastric ulcers and perforations; elevation of hepatic enzymes, hepatocellular liver injury; impaired renal function; Reye’s syndrome; reduces the excretion of uric acid, risks of gout attacks in predisposed patients	cardioprotective value of LD-ASA can be compromised in patients who take NSAIDs concomitantly, because some **NSAIDs** competitively bind to critical amino-acid residues on cyclooxygenase (COX) enzymes and **interfere with the mechanism of the antiplatelet activity of LD-ASA, take ASA hrs in advance; not recommended with:** oral anticoagulants or other NSAIDs or heparins or clopidogrel (beyond the approved indications for this combination in patients with acute coronary syndrome) or ticlopidine or glucocorticoids (except hydrocortisone replacement therapy) for anti-inflammatory doses or anagrelide increased bleeding risk; reduction in the uricosuric effects of benzbromarone, probenecid; increased risk of pemetrexed toxicity in mild-to-moderate renal impairment (creatinine clearance between 45 mL/min and 80 mL/min); precaution in combinations with diuretics, ACE inhibitors, sartans (acute renal failure may occur in dehydrated patients due to decreased GFR secondary to decreased synthesis of renal prostaglandin); methotrexate at doses ≤15 mg/week, requires regular blood counts, clopidogrel; **gastrointestinal topicals, antacids, and charcoal at least 2 h apart; with** deferasirox, thrombolytics, SSRIs (citalopram, escitalopram, fluoxetine, fluvoxamine, paroxetine, sertraline), an increased risk of gastrointestinal ulcers and hemorrhage	not with a history of gastrointestinal bleeding or perforation, related to previous NSAIDs therapy, not with an active or a history of recurrent peptic ulcers/hemorrhages (two or more distinct episodes of proven ulceration or bleeding); not with severe heart failure; not be given to children and adolescents with signs of viral infection aged under 16 years for risk of fatal **Reye’s syndrome,** affecting the brain (vomiting, disturbances of consciousness or abnormal behavior) and liver; not with a history of asthma induced by NSAIDs; esp. 500 mg doses, not in severe renal or hepatic insufficiencies; severe uncontrolled cardiac insufficiency, coadministration with methotrexate > 15 mg/week; coadministration of oral anticoagulants with a history of gastroduodenal ulcers	on long-term (>3 months) with administration every two days or more frequently, medication-overuse headache (MOH) should be suspected, headache induced by overuse of analgesics (MOH); risk of persistent renal lesions and renal insufficiency; in severe G6PD deficiency, ASA may induce hemolysis; monitoring in metrorrhagia or menorrhagia; with alcohol increased bleeding risk
**Atorvastatin** [43]	myalgia, arthralgia; caution in patients with pre-disposing factors for rhabdomyolysis, e.g., renal impairment, hypothyroidism; liver function test abnormal, blood creatine kinase increased; **in elderly (age > 70 years), creatine phosphokinase (CPK) measurements should be considered**, **according to the presence of other predisposing factors for rhabdomyolysis**; patients must be asked to promptly report muscle pain, cramps, or weakness; constipation, flatulence, dyspepsia, nausea, diarrhea; insomnia, headache, dizziness, paresthesia, hypoesthesia; asthenia, chest pain, back pain, peripheral oedema; elevated serum transaminases; hyperglycemia; nasopharyngitis, pharyngolaryngeal pain	**risk of rhabdomyolysis is increased with CsA, TAC, SIR, EVR, clarithromycin, itraconazole, ketoconazole, posaconazole, voriconazole, nefazodone, niacin, gemfibrozil, other fibric acid derivates, or HIV-protease inhibitors, grapefruit juice**, **amiodarone**; oral contraceptives with an increase in plasma concentrations of norethindrone and ethinyl estradiol; also moderate CYP3A4 inhibitors (e.g., erythromycin, diltiazem, verapamil and fluconazole) increase plasma concentrations of atorvastatin; **in general, lovastatin and simvastatin should preferably be avoided in patients treated with CsA, TAC, and SIR, due to the potential rhabdomyolysis; atorvastatin may be used with caution, although the dosage should start low; pravastatin and fluvastatin are the safest alternatives, since they are not metabolized by CYP450 3A4;** all patients treated with HMG-CoA reductase inhibitors should be advised to promptly report any unexplained muscle pain, tenderness, or weakness, particularly if accompanied by malaise or fever; therapy should be discontinued if creatine kinase is markedly elevated in the absence of strenuous exercise or if myopathy is otherwise suspected or diagnosed	with active liver disease or unexplained persistent elevations of serum transaminases exceeding three times the upper limit of normal; in women of childbearing potential not using appropriate contraceptive measures; not with the hepatitis C antivirals gearlever/pibrentasvir; coadministration of potent CYP3A4 inhibitors (e.g., **CsA,** telithromycin, clarithromycin, delavirdine, stiripentol, ketoconazole, voriconazole, itraconazole, posaconazole, and HIV protease inhibitors including ritonavir, lopinavir, atazanavir, indinavir, darunavir, etc.) **should be avoided**	**in primary hypercholesterolemia and combined hyperlipidemia, the majority of patients are controlled with atorvastatin 10 mg/d;** all patients treated with HMG-CoA reductase inhibitors should be advised to promptly report any unexplained muscle pain, tenderness, or weakness, particularly if accompanied by malaise or fever, and checked for CK increase
**Azithromycin** [44]	QTc prolongation (keep serum-Mg^++^ and –K^+^ high normal); diarrhea; vomiting, abdominal pain, nausea; hepatotoxicity (cases of fulminant hepatitis); headache, anorexia, dizziness, paresthesia, dysgeusia; visual impairment; arthralgia; fatigue; deafness; decreased lymphocyte and eosinophil count; decreased blood bicarbonate	**increased exposure of CsA (TAC)**; increased exposure of P-gp substrates, such as digoxin, colchicines; use antacids with an interval of 2 h; rises in theophylline, astemizole, alfentanil levels not investigated, presumed; post-marketing rhabdomyolysis cases with statins; with cisapride or hydroxychloroquine risks of QTc prolongation	not in severe liver dysfunction; not with ergot derivates; not with QTc prolonging drugs	caution in neurologic or psychiatric disorders; in severe renal impairment (GFR < 10 mL/min) 33% increased systemic exposure
**Buprenorphine** [45]	CNS depression, drowsiness; dependence, discontinue gradually, delayed withdrawal syndrome; respiratory depression and death; liver function tests at regular intervals, transient asymptomatic elevations in hepatic transaminases, case reports of cytolytic hepatitis, hepatic failure, hepatic necrosis, hepatorenal syndrome, hepatic encephalopathy, and death; in many cases, the presence of pre-existing liver enzyme abnormalities, infection with hepatitis B, or hepatitis C virus, concomitant use of other potentially hepatotoxic drugs and ongoing injecting drug use may have a causative or contributory role; orthostatic hypotension; caution in elderly or debilitated patients; caution in patients with head injury, increased intracranial pressure, hypotension, prostatic hypertrophy, or urethral stenosis; sleep-related breathing disorders, including central sleep apnea (CSA) and sleep-related hypoxemia; bronchitis, infection, influenza, pharyngitis, rhinitis; lymphadenopathy; insomnia; agitation, anxiety, depression, hostility, nervousness, paranoia, abnormal thinking, headache, dizziness, hypertonia, migraine, paranesthesia, somnolence, syncope, tremor; mydriasis; palpitations; vasodilatation; cough, dyspnea, yawning; nausea; constipation, abdominal pain, diarrhea, dry mouth, dyspepsia, gastrointestinal disorder, flatulence, tooth disorder, vomiting; hyperhidrosis; arthralgia, back pain, bone pain, muscle spasms, myalgia; dysmenorrhea; drug withdrawal syndrome, pain, asthenia, chest pain, chills, malaise, oedema peripheral, pyrexia	reduce dose with CYP3A4 inhibitors (e.g., protease inhibitors ritonavir, nelfinavir or indinavir, azole antifungals, such as ketoconazole and itraconazole, macrolide antibiotics, amiodarone); CYP3A4 inducers decrease buprenorphine plasma concentrations (e.g., phenobarbital, carbamazepine, phenytoin or rifampicin); CNS depression, drowsiness, sedation, respiratory depression, coma, and death, which may be exacerbated by other centrally acting agents, such as alcohol, tranquilizers, sedatives, hypnotics; buprenorphine and other serotonergic agents, such as MAO inhibitors, SSRIs, SNRIs, or tricyclic antidepressants, may result in serotonin syndrome (mental-status changes, autonomic instability, neuromuscular abnormalities, and/or gastrointestinal symptoms); naltrexone is an opioid antagonist that can block the pharmacological effects of buprenorphine	severe respiratory insufficiency; severe hepatic insufficiency; acute alcoholism or delirium tremens; use with care in chronic obstructive pulmonary disease, asthma, cor pulmonale, decreased respiratory reserve, hypoxia, hypercapnia, pre-existing respiratory depression, or kyphoscoliosis; not in severe hepatic impairment	higher plasma levels in patients with moderate and severe hepatic impairment; caution with dosing in patients with severe renal impairment (creatinine clearance < 30 mL/min); some risks of misuse and abuse include overdose, spread of blood borne viral or localized infections, respiratory depression, and hepatic injury; protect children and non-dependent persons against exposure
**Carbamazepine** [46]	leucopenia, thrombocytopenia, eosinophilia; agranulocytosis and aplastic anemia; oedema, fluid retention, weight increase, hyponatremia and blood osmolarity decreased due to an antidiuretic hormone (ADH)-like effect, leading in rare cases to water intoxication, accompanied by lethargy, vomiting, headache, confusional state, neurological disorders; suicidal ideation and behavior; sometimes fatal cutaneous reactions (higher risk in Asians); hypothyroidism; anticholinergic effects; activation of a latent psychosis and, in elderly patients, of confusion or agitation; ataxia, dizziness, somnolence, hypotension, confusional state, sedation, which may lead to falls, fractures, or other injuries; headache, diplopia; accommodation disorders (e.g., blurred vision), lenticular opacities; vomiting, nausea; dry mouth; urticaria, which may be severe dermatitis allergic; fatigue; YGT increased (due to hepatic enzyme induction), usually not clinically relevant; increased AP	**decreased exposure of CSA, TAC, SIR, EVR**; increased exposure and ADRs with inhibitors of CYP3A4 or inhibitors of epoxide hydrolase: dextropropoxyphene, danazol, macrolide antibiotics (e.g., erythromycin, clarithromycin), ciprofloxacine; fluoxetine, fluvoxamine, paroxetine, trazodone, vigabatrin; azoles, e.g., itraconazole, ketoconazole, fluconazole, voriconazole, loratadine, olanzapine, isoniazid, protease inhibitors (e.g., ritonavir), acetazolamide, diltiazem, verapamil, amiodarone, cimetidine, omeprazole, grapefruit juice, nicotinamide; dose adjustment and plasma level monitoring with quetiapine, progabide, valproic acid, valnoctamide, valpromide, primidone, brivaracetam; decreased reliability of hormonal contraceptives may be adversely affected and decreased; potent inducer of CYP3A4 and other phase I and phase II enzyme systems, leads to ineffectiveness of hormonal contraceptives; decreased carbamazepine exposure with CYP3A4 inducers oxcarbazepine, phenobarbital, phenytoin, fosphenytoin, primidone, and possibly clonazepam, cisplatin, or doxorubicin, rifampicin, theophylline, aminophylline, isotretinoin, St. John’s wort; carbamazepine may lower the plasma level, diminish, or even abolish, the activity of certain drugs, the dosage of the following drugs may have to be adjusted for clinical requirements of buprenorphine, methadone, paracetamol tramadol, doxycycline, rifabutin, oral anticoagulants (e.g., warfarin, acenocoumarol, rivaroxaban, dabigatran, apixaban and edoxaban), bupropion, citalopram, mianserin, sertraline, trazodone, tricyclic antidepressants (e.g., imipramine, amitriptyline, nortriptyline, clomipramine), aprepitant, clobazam, clonazepam, ethosuximide, lamotrigine, eslicarbazepine, oxcarbazepine, primidone, tiagabine, topiramate, valproic acid, zonisamide, itraconazole, voriconazole, albendazole, imatinib, cyclophosphamide, lapatinib, temsirolimus, clozapine, haloperidol, bromperidol, olanzapine, quetiapine, risperidone, aripiprazole, paliperidone, indinavir, ritonavir, saquinavir, alprazolam, theophylline, calcium channel blockers (dihydropyridine group), e.g., felodipine, digoxin, simvastatin, atorvastatin, lovastatin, cerivastatin, ivabradine, corticosteroids (e.g., prednisolone, dexamethasone), tadalafil, levothyroxine, estrogens and/or progesterones; levetiracetam may increase carbamazepine-induced toxicity; may increase isoniazid-induced hepatotoxicity; lithium may enhance neurotoxicity; metoclopramide or major tranquilizers, e.g., haloperidol, thioridazine, may increase neurological ADRs; with some diuretics (hydrochlorothiazide, furosemide), a risk of symptomatic hyponatremia; may antagonize the effects of non-depolarizing muscle relaxants (e.g., pancuronium); abstain from alcohol for reduced tolerance; reduced plasma concentrations of DOACs, with risks of thrombosis (rivaroxaban, dabigatran, apixaban, and edoxaban), to avoid phenytoin intoxication and subtherapeutic concentrations of carbamazepine, it is recommended to adjust the plasma concentration of phenytoin to 13 micrograms/mL before adding carbamazepine to the treatment	not in patients with atrioventricular block, a history of bone marrow depression, or a history of hepatic porphyrias (e.g., acute intermittent porphyria, variegate porphyria, porphyria cutanea tarda); not with MAOIs, should be discontinued for a minimum of 2 weeks before starting carbamazepine	only after a critical benefit-risk appraisal and under close monitoring in patients with a history of cardiac, hepatic, or renal damage, adverse hematological reactions to other drugs, or interrupted courses of therapy with carbamazepine; withdraw immediately in cases of aggravated liver dysfunction or acute liver disease; carbamazepine withdrawal should be gradual
**Cidofovir** [47]	dose-dependent nephrotoxicity is the major dose-limiting toxicity; neutropenia; potentially carcinogen; increased risk of developing ocular hypotony with diabetes mellitus; embryotoxic; proteinuria, fever, death (AIDS complications), infection, dyspnea, pneumonia, asthenia, and nausea with vomiting; alopecia	interactions of cidofovir, probenecid, and anti-HIV drugs, including anti-HIV protease inhibitors, have not been investigated in clinical trials, may reduce renal clearance	considered **contraindicated with CsA, TAC, SIR, EVR, and other nephrotoxic drugs,** because of severely enhanced nephrotoxicity; not in patients with renal impairment [serum creatinine > 133 μmol/L (>1.5 mg/dL) or creatinine clearance ≤ 0.92 mL/s (≤55 mL/min) or proteinuria ≥ 100 mg/dL (≥2+ proteinuria)]; not with aminoglycosides, amphotericin B, foscarnet, intravenous pentamidine and vancomycin; direct intraocular injection is contraindicated; safety and efficacy have not been established in patients with hepatic disease	renal function (serum creatinine and urine protein) must be monitored prior to each dose of cidofovir; hydration to minimize the potential for nephrotoxicity; women of childbearing potential should be advised to use effective contraception during and after treatment; men should be advised to practice barrier contraceptive methods during and for 3 months after treatment with cidofovir
**Ciprofloxacin** [48]	triggers **seizures** up to status epilepticus; vision disorders; depression or psychosis can progress to suicide; nausea, diarrhea, vomiting, transient increase in transaminases, rash; potentially irreversible sensory or sensorimotor polyneuropathy, resulting in paranesthesia, hypoesthesia, dysesthesia, or weakness; **QTc prolongation** cave Class IA and III anti-arrhythmics, tricyclic antidepressants, macrolides, antipsychotics, hypokalemia, hypomagnesemia, elderly, cardiac disease; hypoglycemia in diabetic patients; crystalluria; very rare cases of prolonged (continuing months or years), disabling, and potentially irreversible serious adverse drug reactions, affecting different body systems (musculoskeletal, nervous, psychiatric, and sensory); **tendinitis and tendon rupture** (especially but not limited to Achilles tendon), sometimes bilateral, may occur as early as within 48 h of starting treatment with quinolones and fluoroquinolones, and have been reported to occur even up to several months after discontinuation of treatment; the risk of tendinitis and tendon rupture is increased in older patients, patients with renal impairment, patients with solid organ transplants, and those treated concurrently with corticosteroids, concomitant use of corticosteroids should be avoided; photosensitivity reactions, avoid direct exposure to either extensive sunlight or UV irradiation during treatment; **increased risk of aortic aneurysm and dissection**	transient rise in serum creatinine with CsA; ciprofloxacin inhibits CYP1A2 substrates, e.g., theophylline (determination of serum concentration) caffeine or pentoxifylline (oxpentifylline), clozapine, olanzapine, ropinirole, tizanidine, duloxetine, sildenafil, monitor closely for signs of overdose; probenecid reduces renal ciprofloxacin secretion; reduced or increased phenytoin levels; augments vitamin K antagonist effect; increased risk of nephrotoxicity and neurotoxicity with CsA and TAC; **peroral hours apart from Ca^++^, Mg^++^, Fe^++^, Zn^++^, Se^++^**	contraindicated with tizanidine; not with agomelatine, zolpidem; discontinue in symptoms of hepatic diseases; inhibits the renal tubular transport of methotrexate with increased risks of methotrexate toxicity; careful risk-benefit assessment in positive family history of aneurysm disease, or in patients diagnosed with pre-existing aortic aneurysm and/or aortic dissection, or in the presence of other risk factors or conditions predisposing for aortic aneurysm and dissection (e.g., Marfan syndrome, vascular Ehlers-Danlos syndrome, Takayasu arteritis, giant cell arteritis, Behcet’s disease, hypertension, known atherosclerosis)	dose adjustment with impaired renal function; monotherapy not suited for the treatment of severe infections and infections that might be due to Gram-positive or anaerobic pathogens; local prevalence of resistance in *Escherichia coli* to fluoroquinolones; caution in patients with myasthenia gravis
**Citalopram** [49]	paradoxical anxiety; risk of suicide; sleep disorders; agitation, anxiety, nervousness, confusional state, abnormal dreams, anorexia, apathy, somnolence, insomnia, headache, Tremor, paranesthesia, dizziness, disturbance in attention, migraine, amnesia, syncope; asthenia; abnormal accommodation; tinnitus; **hemorrhage**, e.g., SSRIs/SNRIs may increase the risk of postpartum hemorrhage, caution is advised in patients taking SSRIs, particularly in concomitant use with drugs known to affect platelet function, e.g., atypical antipsychotics and phenothiazines, most tricyclic antidepressants, ASA and NSAIDs, or other active substances that can increase the risk of hemorrhage, as well as in patients with a history of bleeding disorders; **hyponatremia** (SIADH); akathisia, restlessness (increasing dose may be detrimental); mania; seizures; alters glycemic control in diabetes mellitus; mydriasis, glaucoma; serotonin syndrome; sexual dysfunction with SSRIs and SNRIs; increase of psychotic symptoms; dose-dependent QTc prolongation (predominantly in female gender, with hypokalemia, or with pre-existing QTc prolongation, or other cardiac diseases), caution advised in bradycardia or in patients with recent acute myocardial infarction or uncompensated heart failure, electrolyte disturbances, such as hypokalemia and hypomagnesemia, increase the risk of malignant arrhythmias and should be corrected before treatment with citalopram is started; yawning, rhinitis; myalgia, arthralgia; urinary retention	**hemorrhage with anticoagulants, ASA, NDAIDs, DOACs, clopidogrel, prasugrel, ticagrelor, dipyridamole, ticlopidine, atypical antipsychotics, phenothiazines, and tricyclic depressants**; not advisable with alcohol; caution with drugs that cause hypokalemia/hypomagnesemia or any other QTc interval prolonging with increased risks of malignant arrhythmias; SSRIs can lower the seizure threshold cumulative with, e.g., antidepressants (tricyclics, SSRIs, SNRIs), neuroleptics (phenothiazines, thioxanthenes, and butyrophenones), mefloquine, bupropion, and tramadol; with CYP2C19 inhibitors omeprazole, esomeprazole, fluconazole, fluvoxamine, lansoprazole, ticlopidine, or cimetidine, moderate increases are likely; **escitalopram (the active enantiomer of citalopram) is an inhibitor of the enzyme CYP2D6, caution when citalopram is coadministered with medicinal products that are mainly metabolized by CYP2D6, and that have a narrow therapeutic index, e.g., flecainide, propafenone, and metoprolol (when used in cardiac failure) or some CNS-acting medicinal products that are mainly metabolized by CYP2D6, e.g., antidepressants such as desipramine, clomipramine, and nortriptyline, or antipsychotics like risperidone, thioridazine, and haloperidol,** dosage adjustment may be warranted; dose reduction may be needed in cases of increased desipramine	not with DOACs; not with and not within 2 weeks of monoamine oxidase inhibitors (MAOIs, selegeline, linezolid, moclobemide), lithium, **risk of serotonin syndrome**; not in patients with known QTc intervals or with medicinal products that are known to prolong the QTc interval; not with linezolid; not with pimozide; should be avoided in patients with unstable epilepsy, patients with controlled epilepsy should be carefully monitored; not with medicinal products with serotonergic effects, such as triptans (including sumatriptan and oxitriptan), opioids (including tramadol and buprenorphine) and tryptophan, due to **risk of serotonin syndrome;** not with St. John’s wort; citalopram with medicinal products that prolong the QTc interval, such as Class IA and III antiarrhythmics, antipsychotics (e.g., phenothiazine derivatives, pimozide, haloperidol), tricyclic antidepressants, certain antimicrobial agents (e.g., sparfloxacin, moxifloxacin, erythromycin IV, pentamidine, anti-malarial treatment particularly halofantrine), and certain antihistamines (astemizole, mizolastine), is contraindicated	risk of suicide may increase in the early stages of recovery; other psychiatric conditions for which citalopram is prescribed can also be associated with an increased risk of suicide-related events; increased risk of suicidal behavior in patients less than 25 years old; caution in the treatment of elderly patients; caution with reduced kidney and liver function; discontinue gradually; ECG monitoring may be advisable in case of overdose or conditions of altered metabolism with increased peak levels, e.g., liver impairment; if signs of cardiac arrhythmia occur during treatment with citalopram, the treatment should be withdrawn, and ECG control is needed
**Clarithromycin** [50]	QTc prolongation; risk of adverse cardiac outcomes; hepatic dysfunction (discontinue in severe course); rhabdomyolysis with statins; statin that is not dependent on CYP3A metabolism (e.g., fluvastatin, pravastatin) can be considered; abdominal pain, diarrhea, nausea, vomiting, and taste perversion; vasodilation; insomnia; dysgeusia, headache	**dose reduction required** for carbamazepine, alprazolam, cilostazol, **CsA, TAC, SIR**, **EVR** (EVR should not be combined) disopyramide, ibrutinib, methadone, methylprednisolone, midazolam, omeprazole, oral anticoagulants, atypical antipsychotics (e.g., quetiapine), quinidine, rifabutin, sildenafil, triazolam and vinblastine, phenytoin, theophylline, and valproate; risk of serious hemorrhage with DOACs and warfarin; caution drugs with QTc prolongation other than the contraindicated, e.g., torsades de pointes with increased quinidine and disopyramide; hypoglycemia with disopyramide; hydroxychloroquine or chloroquine can increase the risk of fatal cardiovascular events; oral hypoglycemics such as nateglinide, repaglinide, and/or insulin can result in significant hypoglycemia; rhabdomyolysis with statins that are not dependent on CYP3A metabolism (e.g., fluvastatin, pravastatin) can be considered; CYP3A4 inducers (rifampicin, phenytoin, carbamazepine, phenobarbital, efavirenz, nevirapine, St. John’s wort) may lead to sub-therapeutic levels of clarithromycin and reduced efficacy, and vice versa, the CYP3A inducers may increase, owing to the inhibition of CYP3A by clarithromycin; exposure decreased by etravirine; only slight increase with fluconazol; ritonavir with concomitant renal impairment require clarithromycin dose reduction; increase in sildenafil, tadalafil, and vardenafil; theophylline, carbamazepine increased; tolterodine increased in poor CYP2D6 metabolizers; zidovudine to 4 h intervals between each medication; **bidirectional drug interactions** with increases in clarithromycin and calcium channel blockers, atazanavir, saquinavir, itraconazole; possible failure of oral contraceptives	not with ergotamine or dihydroergotamine, midazolam; not with astemizole, cisapride, domperidone, pimozide, terfenadine and ivabradine because QTc prolongation and cardiac arrhythmias, not be given to patients with a history of QTc prolongation; not ticagrelor, ranolazine, lomitapide, lovastatin, or simvastatin; not with colchicine; not with hypokalemia or hypomagnesemia; not with severe hepatic failure in combination with renal impairment; combination with **EVR should be avoided**	in patients with renal impairment who have creatinine clearance less than 30 mL/min, the dosage of clarithromycin should be reduced to one half of the normal recommended dose; caution with impaired hepatic function and with moderate-to-severe renal impairment; with coronary artery disease, severe cardiac insufficiency, conduction disturbances, or clinically relevant bradycardia; sensitivity testing must be performed when prescribing clarithromycin for community-acquired pneumonia
**Clopidrogel** [51]	risk of bleeding with hematoma, epistaxis, bleeding at puncture side, eye bleeding, hematuria; gastrointestinal hemorrhage; diarrhea, abdominal pain, dyspepsia, and hematological ADRs; Thrombotic Thrombocytopenic Purpura (TTP): thrombocytopenia and microangiopathic hemolytic anemia, associated with either neurological findings, renal dysfunction, or fever, acquired hemophilia; bruising	increased bleeding risk with ASA, heparin, glycoprotein IIb/IIIa inhibitors, or non-steroidal anti-inflammatory drugs (NSAIDs), including Cox-2inhibitors, selective serotonin reuptake inhibitors (SSRIs), strong CYP2C19 inducers, or other medicinal products associated with bleeding risk, such as pentoxifylline; the concomitant administration with DOACS increases the intensity of bleedings; reduced clopidogrel effect and risk of thrombosis with strong or moderate CYP2C19 inhibitors including, e.g., omeprazole, esomeprazole, fluvoxamine, fluoxetine, moclobemide, voriconazole, fluconazole, ticlopidine, carbamazepine, and efavirenz; with inducers of CYP2C19, e.g., rifampicin, potentiates the risk of bleeding; less pronounced reductions of metabolite exposure has been observed with pantoprazole or lansoprazole compared to omeprazole; significantly reduced platelet inhibition in HIV patients treated with ritonavir or cobicistat-boosted anti-retroviral therapy (similar with nirmatrelvir/ritonavir); increase in repaglinide, paclitaxel exposure; increase in rosuvastatin exposure	not with strong or moderate CYP2C19 inhibitors; not in severe hepatic impairment; not in active pathological bleeding, such as peptic ulcer or intracranial hemorrhage; not with omeprazole; 600 mg loading dose is not recommended in patients with non-ST segment elevation acute coronary syndrome and ≥75 years of age, due to increased bleeding risks in this population	in poor CYP2C19 metabolizers (tests available), clopidogrel at recommended doses forms less of the active metabolite of clopidogrel and has a smaller effect on platelet function; increased bleeding risk from trauma, surgery, or other pathological conditions, if possible, discontinue 7 days prior to surgery; caution in patients who have lesions with a propensity to bleed (particularly gastrointestinal and intraocular); there are no data to support the use of dual antiplatelet therapy in patients for whom treatment with carotid endarterectomy or intravascular thrombectomy is indicated, or in patients planned for thrombolysis or anticoagulant therapy, dual antiplatelet therapy is not recommended in these situations
**Corticosteroids Prednisolone** [52]	a wide range of psychiatric reactions, including affective disorders (such as irritable, euphoric, depressed and labile mood, and suicidal thoughts), psychotic reactions (including mania, delusions, hallucinations, and aggravation of schizophrenia), behavioural disturbances, irritability, anxiety, sleep disturbances, and cognitive dysfunction, including confusion and amnesia; precaution in hypertension, congestive heart failure, and hepatic disease; chickenpox, measles; cataract; Cushing’s disease; osteoporosis, diabetes, hypertension, hypokalemia, susceptibility to infection, and thinning of the skin; adrenocortical insufficiency; renal insufficiency; epilepsy, and/or seizure disorders; dyspepsia, nausea, peptic ulceration with perforation and hemorrhage, abdominal distension, abdominal pain, diarrhea, esophageal ulceration, acute pancreatitis; hirsutism, skin atrophy, bruising, striae, telangiectasia, acne, increased sweating, pruritus, rash, urticaria; proximal myopathy, osteoporosis, vertebral and long bone fractures, avascular osteonecrosis, tendon rupture, tendinopathies (particularly of the Achilles and patellar tendons), myalgia, growth suppression in infancy, childhood, and adolescence; caution in patients with myasthenia gravis receiving anticholinesterase therapy; increased blood coagulability, thromboembolism; scleroderma renal crisis; leukocytosis; sodium and water retention, hypokalemic alkalosis, potassium loss, negative nitrogen and calcium balance, protein catabolism; increase in both high and low density lipoprotein cholesterol, increased appetite, weight gain, obesity, hyperglycemia; congestive heart failure in susceptible patients, hypertension, increased risk of heart failure, increased risk of cardiovascular disease, including myocardial infarction, bradycardia; menstrual irregularity, amenorrhea; fatigue, malaise, impaired healing; increased intra-ocular pressure	risk of seizures in patients receiving CsA with high-dose methylprednisolone; altered blood levels of CsA, TAC with prednisolone (we find decreased trough levels); reduced effect with rifampicin; can lower plasma levels of isoniazid; response to anticoagulants may be reduced or, less often, enhanced by corticosteroids; concurrent insulin and/or oral hypoglycemic agents may require increased dosage; carbamazepine, phenobarbital, phenytoin, and primidone reduce corticosteroid effect; risk of hypokalemia may be increased with amphotericin, combination should be avoided; ketoconazole inhibits metabolism of methylprednisolone and possibly other corticosteroids; with another antimuscarinic drug risk of impairment to memory and attention in the elderly; carbimazole and thiamazole decrease the effect; increased with antiviral drugs, such as ritonavir and indinavir; CsA may increase effects; increased risk of hematological toxicity with methotrexate; CYP3A inducers, such as phenobarbital, phenytoin, rifampicin, rifabutin, carbamazepine, primidone, and aminoglutethimide may reduce the therapeutic efficacy of corticosteroids by increasing the rate of metabolism, lack of expected response may be observed and dosage of deltacortril gastro-resistant tablets may need to be increased; CYP3A4 inhibitors (e.g., ketoconazole, troleandomycin) may increase glucocorticoid effect; oral contraceptives increased prednisolone concentrations by 131%; increased exposure of contraceptives containing ethinylestradiol, mestranol, desogestrel, levonorgestrel, norgestrel, or norethisterone; increased risk of GI ulceration with concomitant ulcerogenic drugs, such as indomethacin; salicylates and corticosteroids should be used concurrently with caution, reduced effect of salicylate and increased at withdrawal; estrogens may potentiate the effects of glucocorticoid; ritonavir possibly increases plasma concentrations of prednisolone and other corticosteroids; the desired effects of hypoglycemic agents (including insulin), antihypertensives, and diuretics are antagonized by corticosteroids; and the hypokalemic effect of acetazolamide, loop diuretics, thiazide diuretics, carbenoxolone, and theophylline are enhanced; increased risk of hypokalemia, if high doses of corticosteroids are given with high doses of bambuterol, fenoterol, formoterol, ritodrine, salbutamol, salmeterol, and terbutaline	not with ocular herpes simplex, because of possible perforation; not with systemic infection, unless specific anti-infective therapy is employed; no live vaccines with high dose corticosteroids	incidence of predictable undesirable effects, including hypothalamic-pituitary adrenal suppression correlates with the relative potency of the drug, dosage, timing of administration, and the duration of treatment; enteric coated form should be used with caution in any condition characterized by diarrhea or a rapid transit time; in patients with acute and active hepatitis, protein binding of the glucocorticoids will be reduced and peak concentrations of administered glucocorticoids increased, elimination of prednisolone will also be impaired, enhanced effect of corticosteroids in patients with cirrhosis; effect of corticosteroids may be reduced for 3–4 days after mifepristone; may suppress reactions to skin tests
**Cyclosporine A (CsA)** [1]	**due to its nephrotoxic potential, the careful monitoring of renal function** is recommended; experience in the elderly is limited, more likely to develop systolic hypertension during therapy, and more likely to show serum creatinine rises ≥ 50% above the baseline after 3 to 4 months of therapy; increases the risk of developing lymphomas and other malignancies, particularly those of the skin, enhanced with treatment regimen containing multiple immunosuppressants, has to avoid excess unprotected sun exposure and should not receive concomitant ultraviolet B irradiation or PUVA photochemotherapy; predisposes patients to the development of a variety of bacterial, fungal, parasitic, and viral infections, often with opportunistic pathogens, the activation of latent polyomavirus infections which may lead to polyomavirus-associated nephropathy (PVAN), especially to BK virus nephropathy (BKVN), or to JC virus-associated progressive multifocal leukoencephalopathy (PML), to be considered in the differential diagnosis in immunosuppressed patients with deteriorating renal function or neurological symptoms, serious and/or fatal outcomes have been reported; frequent and potentially serious increases in serum creatinine and urea, these functional changes are dose-dependent and are initially reversible, usually responding to dose reduction, during long-term treatment, some patients may develop structural changes in the kidney (e.g., interstitial fibrosis), which, in renal transplant patients, must be differentiated from changes due to chronic rejection, the frequent monitoring of renal function, esp. in the elderly, is therefore required; hepatotoxicity and liver injury, including cholestasis, jaundice, hepatitis, and liver failure, necessitates dose reduction; hypertension requires antihypertensives, preferentially those without DDIs; hyperlipidemia; hyperkalemia; **serum Mg^++^ measures for risks of hypomagnesemia**; tremor, headache, **convulsions**, paresthesia; hirsutism; diarrhea, anorexia, nausea, vomiting, abdominal discomfort/pain, diarrhea, gingival hyperplasia, peptic ulcer; leucopenia; hyperlipidemia; hepatic function abnormal; hirsutism, acne, hypertrichosis; myalgia, muscle cramps; pyrexia, fatigue; hearing impairment with high doses; pain of lower extremities has also been noted as part of **calcineurin-inhibitor induced pain syndrome (CIPS)**	caution when coadministering CsA with drugs that substantially increase or decrease CsA blood concentrations, through the inhibition or induction of CYP3A4 and/or P-gp; all inducers of CYP3A4 and/or P-gp are expected to decrease CsA levels; **drugs that decrease CsA levels are metamizole, barbiturates, carbamazepine, oxcarbazepine, phenytoin; nafcillin, intravenous sulfadimidine, probucol, orlistat, St. John’s wort, ticlopidine, sulfinpyrazone, terbinafine, bosentan; rifampicin induces CsA intestinal and liver metabolism, CsA may need to be increased 3- to 5-fold during coadministration;** octreotide decreases the oral absorption of CsA and a 50% increase in the CsA dose, or a switch to intravenous administration could be necessary; **all inhibitors of CYP3A4 and/or P-gp may lead to increased levels of CsA, such as nicardipine, metoclopramide, oral contraceptives, methylprednisolone (high dose) (we have found decreased CsA through blood levels with steroids), allopurinol, cholic acid and derivatives, protease inhibitors, imatinib, colchicine, nefazodone, macrolide antibiotics, e.g., erythromycin can increase CsA exposure 4- to 7-fold, sometimes resulting in nephrotoxicity, clarithromycin has been reported to double the exposure of CsA, azitromycin increases CsA levels by around 20%, azole antimycotics, e.g., ketoconazole, fluconazole, itraconazole, and voriconazole could more than double CsA exposure, verapamil increases CsA blood concentrations two- to threefold, coadministration with telaprevir resulted in approximately 4.64-fold increase in CsA dose normalized exposure (AUC); amiodarone substantially increases the plasma CsA concentration concurrently with an increase in serum creatinine, this interaction can occur for a long time after withdrawal of amiodarone, due to its very long half-life (about 50 days)**, danazol has been reported to increase CsA blood concentrations by approximately 50%, **diltiazem (at doses of 90 mg/day) can increase CsA plasma concentrations by up to 50%,** imatinib could increase CsA exposure by around 20%, cannabidiol (P-gp inhibitor) inhibits intestinal P-gp efflux, leading to the increased bioavailability of calcineurin inhibitor, **grapefruit and grapefruit juice increase CsA**; **exhibit nephrotoxic synergy,** such as aminoglycosides (including gentamycin, tobramycin), amphotericin B, ciprofloxacin, vancomycin, trimethoprim (+sulfamethoxazole); fibric acid derivatives (e.g., bezafibrate, fenofibrate); NSAIDs (including diclofenac, naproxen, sulindac), melphalan histamine H_2_-receptor antagonists (e.g., cimetidine, ranitidine), methotrexate, close monitoring of renal function should be performed, if a significant impairment of renal function occurs, the dosage of the coadministered drug should be reduced, or alternative treatment considered; due to the risk for nephrotoxicity and pharmacokinetic interactions via CYP3A4 and/or P-gp, concomitant use of CsA and TAC should be avoided; CsA is an inhibitor of CYP3A4, the multidrug efflux transporter P-gp and organic anion transporter proteins (OATP); statins increase with risk of rhabdomyolysis; in concomitant administration of **CsA and lercanidipine, the AUC of lercanidipine was increased 3-fold** and CsA 21%, only administer lercanidipine 3 h before CsA, otherwise avoid combination; pharmacokinetics of CsA may be impacted by changes in liver function during DAA therapy, related to the clearance of HCV virus; CsA may reduce the clearance of digoxin, colchicine, statins (dose reductions), and etoposide; with nifedipine, there is an increased risk of gingival hyperplasia; significant increase in diclofenac bioavailability and risk of renal impairment; CsA significantly increases SIR and EVR exposure; significantly elevated hyperkalemia risk with potassium-sparing diuretics, ACE inhibitors, angiotensin II receptor antagonists, and potassium-containing drugs; increased repaglinide with risks of hypoglycemia; increased ambrisentan exposure; increased anthracycline antibiotics (e.g., doxorubicin, mitoxantrone, daunorubicin); **CsA with mycophenolate sodium or mycophenolate mofetil in transplant patients may decrease the mean exposure MPA by 20–50%,** when compared with other immunosuppressants, to be considered esp. in the case of the interruption or discontinuation of CsA therapy; decreases eltrombag exposure	decreased CsA with St, John’s Wort; **not with substrates for the multidrug efflux transporter P-gp or the organic anion transporter proteins (OATP), and for which elevated plasma concentrations are associated with serious and/or life-threatening events, e.g., bosentan, dabigatran etexilate, and aliskiren;** vaccination less effective, the use of live attenuated vaccines should be avoided; in non-transplant patients, the relationship between blood level and clinical effects is less well established, if drugs known to increase CsA levels are given concomitantly, the frequent assessment of renal function and the careful monitoring of CsA-ADRs may be more appropriate than blood level measurement; not with aliskiren; not recommended with dabigatran; not with bosentan, CsA exposure is decreased by 35%, and bosentan exposure increased several-fold; at increased risks of acute or chronic nephrotoxicity, cases of acute nephrotoxicity reported disorders of ion homeostasis, such as hyperkalemia, hypomagnesemia, and hyperuricemia, cases reporting chronic morphological changes included arteriolar hyalinosis, tubular atrophy, and interstitial fibrosis	TDM is an important safety measure; CsA is extensively metabolized by the liver, an **approximate 2- to 3-fold increase in CsA exposure may be observed in patients with hepatic impairment**, dose reduction may be necessary in patients with severe liver impairment; **higher doses of oral CsA or the use of CsA intravenous therapy, may be necessary in the presence of gastrointestinal disturbances, which might decrease absorption**; due to its nephrotoxic potential, the careful monitoring of renal function is recommended, adequate follow-up, including regular full physical examination, blood pressure measurements, and control of laboratory safety parameters; **transplantation patients on CsA should be managed in facilities with adequate laboratory and supportive medical resources; the physician responsible for maintenance therapy should receive complete information for the follow-up of the patient**
**Empaglifozin** [53]	ketoacidosis (nausea, vomiting, anorexia, abdominal pain, excessive thirst, difficulty breathing, confusion, unusual fatigue, or sleepiness); risk for volume depletion by osmotic diureses with a decrease in blood pressure, therefore caution in patients with known cardiovascular disease, patients on antihypertensive therapy with a history of hypotension, or patients aged 75 years and older, in case of conditions that **may lead to fluid loss** (e.g., gastrointestinal illness), with coadministered drugs, leading to **volume depletion** (e.g., diuretics, ACE inhibitors); thirst; **complicated urinary tract infections,** including pyelonephritis and urosepsis; vaginal moniliasis, vulvovaginitis, balanitis, and **other genital infection**; necrotizing fasciitis of the perineum, **(Fournier’s gangrene)**, advise patients for early symptoms, **awareness that either urogenital infection or perineal abscess may precede necrotizing fasciitis**; increase in cases of lower limb amputation (primarily of the toe); hepatic injury; positive glucose test in urine according to the drug’s mechanism of action; hypoglycemia when used with sulphonylurea or insulin; constipation; increased serum lipids	adds to the diuretic effect of thiazide and loop diuretics, and may increase the risk of dehydration and hypotension; insulin and insulin secretagogues, such as sulphonylureas, may increase the risk of hypoglycemia, therefore, a lower dose of insulin or an insulin secretagogue may be required; co-treatment with known UTG inducers (e.g., by rifampicin or phenytoin) not recommended, due to a potential risk of decreased efficacy; may increase renal lithium excretion	should not be used in patients with type 1 diabetes, increased ketoacidosis occurrence with empagliflozin 10 mg and 25 mg as an adjunct to insulin; not recommended with eGFR < 20 mL/min/1.73 m^2^ due to limited experience	monitor renal function, dose reduction with eGFR < 60 mL/min/1.73 m^2^; interrupt SGLT2 inhibitors in patients who are hospitalized for major surgical procedures or acute serious medical illnesses; higher risk of ketoacidosis with beta-cell function reserve (e.g., type 2 diabetes patients with low C-peptide or latent autoimmune diabetes in adults (LADA) or with a history of pancreatitis), with conditions that lead to restricted food intake or severe dehydration, patients for whom insulin doses are reduced, and patients with increased insulin requirements due to acute medical illness, surgery, or alcohol abuse; the glucose lowering efficacy of empagliflozin is dependent on renal function, reduced in patients with an eGFR < 45 mL/min/1.73 m^2^ and is likely absent in patients with an eGFR < 30 mL/min/1.73 m^2^
**Everolimus (EVR)** [8]	stomatitis, including mouth ulcerations and oral mucositis; hypertriglyceridemia, hypercholesterolemia, hyperglycemia; hypophosphatemia, hypokalemia; hypocalcemia; dehydration; opportunistic infections (e.g., pneumocystis jirovecii (carinii) pneumonia (PJP/PCP), thrombocytopenia, neutropenia, leukopenia, lymphopenia, anemia; non-infectious pneumonitis; localized and systemic infections, including pneumonia, other bacterial infections, invasive fungal infections, such as aspergillosis, candidiasis, or PJP/PCP, and viral infections, including the reactivation of hepatitis B virus with risk of sepsis or respiratory and hepatic failure; proteinuria, renal failure; insomnia, dysgeusia, headache; eyelid oedema; hemorrhage, hypertension, lymphoedema; pneumonitis, epistaxis, cough, dyspnea; diarrhea, nausea, vomiting, dyspepsia, dysphagia; increased AST, ALT; rash, pruritus, dry skin, skin lesions; arthralgia; menstruation irregular; fatigue, asthenia, oedema peripheral; pyrexia; decreased weight; impaired wound healing, caution in the peri-surgical period; severe radiation reactions (such as radiation esophagitis, radiation pneumonitis, and radiation skin injury), including fatal cases, during or shortly after radiation therapy, the potentiation of radiotherapy toxicity in close temporal relationship with radiation therapy and radiation recall syndrome (RRS)	large increased exposure with coadministered strong CYP3A4 and/or Pgp inhibitors and thus not recommended (ketoconazole, itraconazole, posaconazole, voriconazole, telithromycin, clarithromycin, nefazodone, ritonavir, atazanavir, saquinavir, darunavir, indinavir, nelfinavir), caution and decrease dosage with moderate CYP3A4/Pgp inhibitors (erythromycin, imatinib, verapamil, **CsA** p.o., cannabidiol, fluconazole, diltiazem, dronedarone, amiodarone, amprenavir, fosamprenavir, grapefruit juice); decreased exposure and not recommended with inducers of CYP3A4 and/or Pgp (rifampicin, carbamazepine, phenobarbital, phenytoin, dexamethasone, efavirenz, nevirapine, St. John’s Wort) should be avoided, if unavoidable, increase the everolimus dosage with monitoring, and respect the washout phase of at least 3–5 days of the inducer’s effect; prophylaxis for PJP/PCP to be considered when concomitant use of corticosteroids or other immunosuppressive agents are required; with ACE, increased risk for angioedema; caution with orally administered CYP3A4 substrates with a narrow therapeutic index (e.g., pimozide, terfenadine, astemizole, cisapride, quinidine, or ergot alkaloid derivatives), monitor for ADRs	not in severe hepatic impairment (Child-Pugh C), only recommended if the desired benefit outweighs the risk, a dose of 2.5 mg daily must not be exceeded; dose adjustment due to adverse reactions; in invasive systemic fungal infections, discontinue promptly and permanently; stop in radiation recall syndrome (RRS)	dose reduction with mild and moderate hepatic impairment; ADRs leading to the discontinuation of the medicinal product were higher in patients ≥ 65 years, most common ADRs leading to discontinuation were pneumonitis (including interstitial lung disease), stomatitis, fatigue, and dyspnea; live vaccines (intranasal influenza, measles, mumps, rubella, oral polio, BCG (Bacillus Calmette–Guérin), yellow fever, varicella, and TY21a typhoid vaccines) should be avoided
**Febuxostat** [54]	liver function abnormalities, liver function test is recommended prior to the initiation and periodically thereafter; **increased TSH values**; gout flares, diarrhea, nausea; headache, rash, oedema; **atrial fibrillation, palpitations, ECG abnormal**; renal failure	concomitant mercaptopurine or azathioprine not recommended; coadministration with rosiglitazone or other CYP2C8 substrates is not expected to require dose adjustment for those compounds; febuxostat metabolism depends on uridine glucuronosyl transferase (UGT) enzymes, medicinal products that inhibit glucuronidation, such as NSAIDs and probenecid, may increase febuxostat; potent inducers of UGT enzymes might increase metabolism and decrease the efficacy of febuxostat; febuxostat is a weak inhibitor of CYP2D6, a small increase in AUC of desipramine, a CYP2D6 substrate	**in patients with ischemic heart disease or congestive heart failure, not recommended;** not with mercaptopurine/azathioprine; **no experience in organ transplant recipients, the use in such patients is not recommended**	
**Felodipine** [55]	significant hypotension with subsequent tachycardia; peripheral oedema; headache; flush; the renal vascular resistance is decreased by felodipine; in CsA-treated renal transplant recipients, felodipine improves both the renal blood flow and the glomerular filtration rate, and may also improve early renal graft function	AUC increased sixfold when coadministered with the strong CYP3A4 inhibitor itraconazole, with erythromycin AUC increasing 2.5-fold; felodipine dose reduction required in coadministered CYP3A4 inhibitors; **CsA may significantly increase serum felodipine concentrations and vice versa via the inhibition of CYP3A4 first pass metabolism, amlodipine undergoes less first-pass metabolism, to be considered as an alternative; TAC and SIR expose increased**	not in decompensated heart failure, unstable angina pectoris, hemodynamically significant cardiac valvular obstruction, dynamic cardiac outflow obstruction	dose reduction in impaired hepatic function; strong CYP3A4 inhibitors and grapefruit juice should be avoided; plasma protein (albumin) binding is approximately 99%
**Fentanyl** [56]	life-threatening respiratory depression; potential for serious or life-threatening hypoventilation exists, even if the lowest dose is used in initiating therapy in opioid-naïve patients, especially in elderly or patients with hepatic or renal impairment; fentanyl patches may have more severe adverse effects in patients with chronic obstructive or other pulmonary disease, in such patients, opioids may decrease respiratory drive and increase airway resistance; if withdrawal symptoms are experienced, do not discontinue abruptly; tolerance, physical, and psychological dependence; repeated use of fentanyl may lead to opioid use disorder (OUD with drug seeking behavior); increased skin temperature, potential for temperature-dependent increases in fentanyl released from the system, resulting in possible overdoses and death, patients to be advised to avoid exposing the application site of fentanyl patches to direct external heat sources, such as heating pads, electric blankets, heated water beds, heat or tanning lamps, sunbathing, hot water bottles, prolonged hot baths, saunas, and hot whirlpool spa baths; bradycardia; hypotension; constipation (prophylactic laxative be considered); caution in myasthenia gravis; opioid-induced hyperalgesia (OIH); anorexia; insomnia, depression, anxiety, confusional state, hallucination; somnolence, dizziness, headache, tremor, paresthesia; vertigo, tachycardia, palpitations, bradycardia; dry mouth, nausea, vomiting, abdominal pain, hyperhidrosis, muscle spasms, urinary retention, fatigue, peripheral oedema, asthenia, malaise, feeling cool	caution with coadministered drugs that affect the serotonergic neurotransmitter systems (SSRIs, SNRIs, MAOIs), risk of potentially life-threatening **serotonin syndrome**; with benzodiazepines or other CNS depressants, including alcohol, may result in profound sedation, respiratory depression, coma, and death; **not recommended with CYP3A4 inhibitors** (**amiodarone, cimetidine, clarithromycin, diltiazem, erythromycin, fluconazole, itraconazole, ketoconazole, nefazodone, ritonavir, verapamil, and voriconazole**—this list is not exhaustive, e.g., to a lesser extend also CsA), they increase fentanyl plasma concentrations, generally, a patient should wait 2 days after stopping treatment with a CYP3A4 inhibitor before applying the first fentanyl patch; however, the duration of inhibition varies, and for some CYP3A4 inhibitors with a long elimination half-life, such as amiodarone, or for time-dependent inhibitors, such as erythromycin, idelalisib, nicardipine, and ritonavir, this period may need to be longer, a patient who is treated with fentanyl patches should wait at least 1 week after the removal of the last patch before initiating treatment with a CYP3A4 inhibitor; not with concomitant buprenorphine, nalbuphine, or pentazocine; **with CYP3A4 inducers, decrease in fentanyl plasma concentrations** and a decreased therapeutic effect (by carbamazepine, phenobarbital, phenytoin and rifampicin—list not exhaustive); effects of the inducer decline gradually and may result in increased fentanyl plasma concentrations	not with MAOIs or within 14 days of such therapy; suspected surgical abdomen; acute or perioperative pain; opioid-I patients; respiratory depression; acute or severe bronchial asthma; mechanical gastrointestinal obstruction, ileus of any type; acute alcoholism, delirium tremens, and convulsive disorders; increased cerebrospinal or intracranial pressure, and head injury; alcohol serious additive injury, potentially death; fentanyl patch should only be prescribed to patients who require continuous opioids for pain management, and who are tolerant to at least the morphine equivalent of the lowest initiating fentanyl patch dose; dose reduction in hepatic impairment because of the risk of toxicity; caution in renal impairment	in elderly, cachectic, or debilitated patients, fentanyl transdermal system may have altered pharmacokinetics due to poor fat stores, muscle wasting, or altered clearance, initiate on a low dose; risks of addiction, abuse, and misuse; only to be used in patients for whom alternative treatment options are ineffective or not tolerated (e.g., non-opioid analgesics), or would be otherwise inadequate to provide sufficient management of pain (e.g., immediate-release opioids); accidental exposure, such as in cases of hugging, sharing a bed, or moving a patient, esp. in children, can lead to serious medical consequences, including death; patients and their carers must be advised to keep fentanyl in a safe and secure place, not accessible by others; elderly patients may have reduced clearance, a prolonged half-life, and they may be more sensitive to the active substance
**Fluconazole** [57,58]	may affect liver; headache; stomach discomfort, feeling sick, vomiting; increases in blood tests of liver function; QTc prolongation, strongly inhibits the HERG channel, potentially proarrhythmic conditions, such as congenital or documented acquired QTc prolongation, cardiomyopathy, particularly when heart failure is present, sinus bradycardia, existing symptomatic arrhythmias, concomitant medication known to prolong QTc interval, hypokalemia, hypomagnesemia, and hypocalcemia should be corrected prior to the initiation of fluconazole treatment; adrenal insufficiency with chronic or long lasting fatigue, muscle weakness, loss of appetite, weight loss, abdominal pain, patients receiving long-term therapy with fluconazole and prednisone should be closely monitored for signs of adrenal insufficiency when fluconazole is withdrawn	**increase in CsA, TAC (5-fold if peroral, nephrotoxicity increased risk), SIR, EVR, needs dose reduction**; caution with loperamide and QTc prolongation; caution because of inhibited metabolizm and QTc prolongation with amidodarone; rifampicin and rifabutin reduce fluconazole bioavalability and require fluconazole dose elevation; increased abrocitinib, olaparib, ibrutinib, ivacaftor, lurasidone, methadone, naproxen, lornoxicam, meloxicam, diclofenac, phenytoin, rifabutin exposure, dose reduction required; increased alfentanil, fentanyl (can lead to respiratory depression), amitriptyline, nortriptyline, theopfylline, trimetrexate, zidovodine, dose reduction required; amphotericin B in therapy likely antagonizes systemic *Aspergillus fumigatus*; enhanced anticoagulative effects of coumarin or indandione drugs; elevated midazolam and triazolam exposures require dose reduction; risk of carbamazepin toxicity should lead to extensive monitoring; elevated exposure of nifedipine, isradipine, amlodipine, verapamil, lercanidipine, felodipine, celecoxib; with cyclophosphamide, increased bilirubin and crea tinin serum concentrations; risk of rhabdomyolysis with atorvastatin, simvastatin, fluvastatin; decreased activation of losartan; prolongs the plasma half-life of concomitantly administered sulphonyl urea (chlorpropamide, glibenclamide, glipizide, and tolbutamide) with risk of hypoglycema, voriconazole warfarin, or similar medicines, carbamazepine, phenytoin, nifedipine, isradipine, amlodipine, felodipine, and losartan, **CsA, TAC, SIR, EVR**, cyclophosphamide, vinca alkaloids (vincristine, vinblastine, or similar medicines), halofantrine, statins (atorvastatin, simvastatin, and fluvastatin), methadone, celecoxib, flurbiprofen, naproxen, ibuprofen, lornoxicam, meloxicam, diclofenac, oral contraceptives, prednisone, zidovudine, saquinavir, chlorpropamide, glibenclamide, glipizide, or tolbutamide, theophylline, vitamin A, ivacaftor, amiodarone, hydrochlorothiazide; risk of adrenal insufficiency in combination with prednisone	**caution in liver impairment; not with drugs that prolong QTc interval and are metabolized via CYP3A4, such as astemizole, terfenadine, cisapride, pimozide, quinidine, erythromycin, halofantrine**	dose adjustment to renal function; fluconazole is a moderate CYP2C9 and CYP3A4 inhibitor, and a strong inhibitor of CYP2C19
**Fluvastatin** [59]	blood creatine phosphokinase increased, blood transaminases increased; hepatic failure; increase in AST or ALT three times the upper limit of normal and persisting, therapy should be discontinued; insomnia; headache; nausea, abdominal pain, dyspepsia; pancreatitis; myalgia, muscular weakness, myopathy; sleep disturbances, insomnia, nightmares; memory loss; depression; interstitial lung disease; diabetes mellitus; interstitial lung disease; hyperglycemia; tendinopathy; very rarely immune-mediated necrotizing myopathy (IMNM); caution in patients with predisposing factors for rhabdomyolysis and its complications; creatine kinase level before starting treatment in the following situations (predisposing factors for rhabdomyolysis): renal impairment, hypothyroidism, personal or familial history of hereditary muscular disorders, previous history of muscular toxicity with a statin or fibrate, alcohol abuse, sepsis, hypotension, excessive exercise of muscles, major surgery, severe metabolic, endocrine or electrolyte disorders, elderly (age > 70 years)	increased risk of myopathy/rhabdomyolysis with bezafibrate, gemfibrozil, ciprofibrate, or niacin (nicotinic acid), immunosuppressive agents (including **CsA**), erythromycin, colchicine; for warfarin or other coumarin derivatives, recommendation to monitor prothrombin times; rifampicin reduces exposure; with glibenclamide, increases of both; with bile acid sequestrants, at least 4 h after the resin (e.g., cholestyramine) to avoid a significant interaction due to the drug binding of the resin; may be used with azole antifungal agents, except for fluconazole and voriconazole, both of which can inhibit fluvastatin metabolism via CYP450 2C9; with the potent cytochrome P450 (CYP) 3A4 inhibitors itraconazole and erythromycin, minimal effects on the bioavailability	not in patients with active liver disease, or unexplained, persistent elevations in serum transaminases; not with systemic formulations of fusidic acid or within 7 days of stopping fusidic acid (rhabdomyolysis)	as with other lipid-lowering agents, recommended liver function tests before initiation of treatment and at 12 weeks following initiation of treatment, or elevation in dose and periodically thereafter; cases of hepatic failures with some statins including fluvastatin; caution in history of liver disease and heavy alcohol ingestion; CK not to be measured following strenuous exercise
**Foscarnet** [60]	**renal, myelosuppressive, and neurotoxicity (severe seizures); rate of infusion must be no more than 1 mg/kg/min; hydration is recommended with each infusion to reduce renal toxicity; hypocalcemia, hypophosphatemia, hyperphosphatemia, hypomagnesemia, hypokalemia**; potential to chelate divalent metal ions, such as calcium and magnesium; seizures (associated with death) related to mineral and electrolyte abnormalities; QTs prolongation; saline load (caution in cardiomyopathy); perioral tingling, numbness in the extremities or paresthesia during or after infusion as possible symptoms of electrolyte abnormalities; potentially carcinogenesis, mutagenesis, impairment of ncreasty, teratogenic effect; fever; nausea; diarrhea; anemia; renal toxicity; seizures; marrow suppression; fever, fatigue, rigors, asthenia, malaise, pain, infection, sepsis, death; depression, confusion, anxiety; vision abnormalities; urine concentrations genital irritations, ulcers	decreases serum concentrations of ionized calcium, concurrent treatment with other drugs known to influence serum calcium concentrations should be used with particular caution; avoid combination with potentially nephrotoxic drugs, such as aminoglycosides, CsA, TAC, amphotericin B, aciclovir, methotrexat, and intravenous pentamidine; renal impairment and symptomatic hypocalcemia (Trousseau’s and Chvostek’ signs) with i.v. pentamidine; abnormal renal function with ritonavir, saquinavir; when diuretics are indicated, thiazides are recommended; avoid QTc prolonging drugs (e.g., quinidine, amiodarone, sotalol)—antiarrhythmic or neuroleptic agents	not with pentamidine i.v.—enhanced hypocalcemia and nephrotoxicity	initial and maintenance therapy with dose adjustment to renal function; monitor renal function every second day during induction therapy, and once weekly during maintenance therapy, adequate hydration, monitor serum calcium and magnesium
**Ganciclovir, Valganciclovir** [61,62]	neutropenia, anemia, thrombocytopenia; infections; complete blood counts and platelet counts should be monitored regularly during therapy, increased hematological monitoring may be warranted in patients with renal impairment and pediatrics; decreased appetite and weight; depression, confusion, anxiety; headache, dizziness, hypo-paresthesia, seizure, dysgeusia; visual impairment; ear pain; hypotension; cough, dyspnea; diarrhea, nausea; vomiting, dyspepsia, flatulence, constipation, dysphagia, pancreatitis; hepato-biliary disorders; dermatitis; night sweats; back pain, myalgia, arthralgia, muscle spasms; renal impairment; pyrexia; fatigue; pain, asthenia; potential teratogen and carcinogen in humans, caution should be observed in handling broken tablets; retinal detachment reported in AIDS patients treated for CMV retinitis	**with CsA and TAC enhanced risk of myelotoxicity, nephrotoxicity**, and neurotoxicity; with MMF or trimethoprim, enhanced myelosuppressive toxicity; seizures with imipenem-cilastatin; probenecid competition for renal tubular secretion enhances toxicity; increased didanosine toxicity, pancreatitis; renal impairment enhances risk of myelosuppression; **toxicity may be enhanced with other drugs known to be myelosuppressive or associated with renal impairment,** such as nucleoside (e.g., zidovudine, didanosine, stavudine) and nucleotide analogues (e.g., tenofovir, adefovir), **immunosuppressants (e.g., CsA, TAC, MMF**), antineoplastic agents (e.g., doxorubicin, vinblastine, vincristine, hydroxyurea), and anti-infective agents (trimethoprim/sulphonamides, dapsone, amphotericin B, flucytosine, pentamidine)—these drugs should only be considered for concomitant use with valganciclovir if the potential benefits outweigh the potential risks	should not be initiated if the absolute neutrophil count is less than 500 cells/μL, or the platelet count is less than 25,000/μL, or the hemoglobin level is less than 8 g/dL; antiviral drugs that share the tubular secretion pathway enhance exposure, e.g., nucleos(t)ide analog reverse transcriptase inhibitors (NRTIs) (including those used for HBV therapy), e.g., lamivudine, emtricitabine, tenofovir, adefovir, and entecavir; renal clearance inhibited, due to nephrotoxicity caused by drugs, such as cidofovir, foscarnet, NRTIs (e.g., tenofovir, adefovir)—concomitant use of valganciclovir with any of these drugs should be considered only if the potential benefits outweigh the potential risks; toxicity may be enhanced with, or is given immediately before or after, other drugs that inhibit the replication of rapidly dividing cell populations, such as in bone marrow, testes, and germinal layers of the skin, and gastrointestinal mucosa, such as dapsone, pentamidine, flucytosine, vincristine, vinblastine, Adriamycin, amphotericin B, trimethoprim/sulfa combinations, nucleoside analogues and hydroxyurea, and pegylated interferons/ribavirin (with or without boceprevir or telaprevir)	dose adjustment to renal function; not in patients on hemodialysis; safety and efficacy of valganciclovir tablets have not been studied in patients with hepatic impairment, not in elderly patients; if there is a significant deterioration of blood cell counts during therapy with valganciclovir, treatment with hematopoietic growth factors and/or dose interruption should be considered; when extending prophylaxis beyond 100 days, the possible risk of developing leucopenia and neutropenia should be taken into account
**Ibuprofen** [63]	dose-dependent reduction in prostaglandin formation and precipitate renal failure—at increased risk with impaired renal function, cardiac impairment, liver dysfunction, those taking diuretics, and the elderly; lasting severe renal impairment, increased under physical strain associated with loss of salt and dehydration; prolonged use of any type of analgesic for headaches can make them worse; medication-overuse headache (MOH); GI bleeding, ulceration or perforation; precipitate bronchospasm, urticaria, or angioedema; caution heart failure or hypertension	**with TAC, CsA increased risk of nephrotoxicity**; corticosteroids increased risk of gastrointestinal ulceration or bleeding with NSAIDs; may affect or be affected by anti-coagulants ASA, warfarin, ticlopidine, medicines that reduce high blood pressure (ACE-inhibitors, beta-blockers angiotensin-II receptor antagonists); CYP2C9 inhibitors, e.g., voriconazole and fluconazole, increase the ibuprofen exposure; quinolones increased risk of developing convulsions; **anti-platelet agents and SSRIs/SNRIs leads to an increased risk of gastrointestinal bleeding with NSAIDs**; NSAIDs may decrease the excretion of aminoglycosides; increased risk of hematological toxicity with zidovudine; may potentiate the effects of sulfonylurea medications with hypoglycemia; cardioprotective value of LD-ASA can be compromised in patients who take NSAIDs concomitantly, because some NSAIDs competitively bind to critical amino-acid residues on cyclooxygenase (COX) enzymes and interfere with the mechanism of antiplatelet activity of LD-ASA, **important to take ASA hrs in advance to NSADIs**; enhanced effects of methotrexate, lithium, probenecid, and sulphinpyrazones; increased risk of gastrointestinal ulcers with bisphosphonates; quinolone antibiotics increased risk for convulsions; zidovudine increased risk of bleeding	**not with TAC, CsA**; **not with DOACs**; previous allergic reaction such as asthma, difficulty in breathing, swelling of the face, tongue, or throat nettle rash, itchy runny nose attributed to acetylsalicylic acid (ASA) or other NSAIDs; stomach ulcer or bleeding; gastrointestinal perforation or bleeding when taking NSAIDs; suffering from cerebrovascular or other active bleeding; suffering from unclarified blood-formation disturbances; severe dehydration (caused by vomiting, diarrhea, or insufficient fluid intake); severe liver, kidney, or heart failure	minimum effective dose for the shortest period of time; the elderly are at increased risk of ADRs; combination therapy with protective agents, such as PPIs; NSAIDs may mask symptoms of infection and fever; Ginkgo biloba may potentiate the risk of bleeding with NSAIDs; monitor renal function
**Lercanidipine** [64]	headache, dizziness, peripheral oedema, tachycardia, palpitations, flushing; care in patients with sick sinus syndrome, with LV dysfunction, ischemic heart disease; overdose might be expected to cause excessive peripheral vasodilatation with marked hypotension and reflex tachycardia	potentiates the effect of vasodilating antihypertensive medicines; reduced exposure by inducers of CYP3A4, like anticonvulsants (e.g., phenytoin, carbamazepine) and rifampicin; increased exposure with CYP3A4 (e.g., ketoconazole (15-fold AUC increase), itraconazole, ritonavir, erythromycin, troleandomycin), avoid combination; caution with CYP3A4 substrates, like terfenadine, astemizole, class III antiarrhythmic medicines, such as amiodarone or quinidine; metoprolol reduced lercanidipine bioavailability by 50%, likely due to reduced hepatic blood flow caused by β-blockers; increased digoxin exposure; with simvastatin 12 h apart recommended	not in patients with left ventricular outflow tract obstruction, untreated congestive cardiac failure, unstable angina pectoris, severe renal, or hepatic impairment, within 1 month of a myocardial infarction; not with strong inhibitors of CYP3A4, **not with CsA (3-fold increase of the plasma levels of lercanidipine and a 21% increase of the CsA AUC)**, grapefruit juice; not in patients with severe hepatic impairment or with severe renal impairments (GFR < 30 mL/min)	gradual dose titration, as it may take about 2 weeks before the maximal antihypertensive effect is apparent; antihypertensive effect may be enhanced in patients with hepatic impairment, dose adjustment required
**Letermovir** [65]	nausea, diarrhea, vomiting common; increase in blood creatinine or alanine aminotransferase, aspartate aminotransferase uncommon	in CYP3A substrates, such as **alfentanil, fentanyl, midazolam, quinidine, CsA, TAC, SIR, coadministration increased exposure (close monitoring and dose adjustment**); increased OATP1B1/3 transported statins; through inducing effects, **reduced voriconazole and dabigatran exposure**; combination of CsA and letermovir may lead to more marked or additional effects on concomitant medicinal products as compared to letermovir alone; increase in amiodarone (frequent monitoring of concentrations), SIR, TAC, quinidine are expected, but not yet studied; decrease in omeprazole, pantoprazole, voriconazole, oral contraceptive steroids, warfarin, dabigatran	not with severe hepatic impairment; not with combined moderate hepatic and moderate renal impairment; subtherapeutic letermovir exposure through rifampicin, rifabutin, thioridazine, bosentan, phenytoin, carbamazepine, St. John’s wort, phenobarbital, efavirenz, etravirine, nevirapine, ritonavir, lopinavir, modafinil; in combination with CsA, concomitant use of dabigatran, atorvastatin, simvastatin, rosuvastatin, or pitavastatin is contraindicated; fluvastatin, pravastatin increased exposure; pimozide; ergot alkaloids	**CsA (potent OATP1B1/3 inhibitor), requires decreased letermovir dose of 240 mg/d;** caution if other OATP1B1/3 inhibitors are added to letermovir combined with CsA, such as gemfibrozil, erythromycin, clarithromycin, and several protease inhibitors (atazanavir, simeprevir); monitoring of CMV DNA; it is still unknown whether letermovir may reduce the exposure of piperacillin/tazobactam, amphotericine B, and micafungin; extensively bound (98.2%) to human plasma proteins
**Linezolid** [66]	myelosuppression; lactic acidosis; increased LDH, lipase, AST, ALT or alkaline phosphatase; diarrhea, nausea, vomiting; headache; peripheral and optic neuropathy; risk of serotonin syndrome	linezolid may enhance the increases in blood pressure caused by pseudoephedrine and phenylpropanolamine hydrochloride	**not with and not within two weeks of taking monoamine oxidase A or B inhibitors; not with serotonergic agents; not with tyramine rich foods**	caution in patients with severe renal insufficiency, and in patients with severe hepatic insufficiency
**Loperamide** [67]	constipation; risk for toxic megacolon; CNS depression, urinary retention, **QTc interval and QRS complex prolongation and torsades de pointes, other serious ventricular arrhythmias, cardiac arrest and syncope in association with overdose, hepatic impairment, or DDI-induced overexposure, some cases had a fatal outcome**	**increased exposure and risk of cardiotoxicity with drugs that inhibit CYP3A4** (e.g., azole antifungal agents, clarithromycin, cobicistat, conivaptan, delavirdine, erythromycin, idelalisib, nefazodone, protease inhibitors, telithromycin) and CYP2C8 (e.g., gemfibrozil, clopidogrel) with inhibitors of P-glycoprotein transport (e.g., amiodarone, **CsA**, diltiazem, dronedarone, quinidine, verapamil) and hepatic impairment	not in children < 12 yrs; not in acute severe inflammatory colitis or acute dysentery with blood in stool and fever	caution in hepatic impairment
**Metamizole (Dipyrone)** [68,69]	agranulocytosis; pancytopenia; severe skin reactions; deterioration of renal impairment, interstitial nephritis; parenteral administration is associated with a high risk of anaphylactic/anaphylactoid reactions; risk of a hypotensive reaction; hepatotoxicity	**decrease in blood levels of CsA and TAC SIR, EVR requires dose elevation** and monitoring; increased methotrexate hematotoxicity, in particular in elderly patients, combination to be avoided; **reduced efficacy of ASA on platelet aggregation, take ASA hours in advance** or avoid combination; reduction in bupropion exposure and increase of its active metabolite hydroxybupropion; upropion; reduced bioavailability of efavirenz, methadone, valproate, sertraline	previous experience with agranulocytosis; hypotension or unstable hemodynamics, impaired bone marrow function (e.g., after chemotherapy), or impaired hematopoiesis; analgesic asthma or analgesic intolerance of the urticaria/angioedema type, i.e., patients with a known occurrence of bronchospasm or other anaphylactoid reactions (e.g., urticaria, rhinitis, angioedema) after the administration of salicylates, paracetamol, or other on-narcotic analgesics, e.g., diclofenac, ibuprofen, indomethacin, naproxen; elimination rate is reduced when renal or hepatic function is impaired, multiple high doses should be avoided	**dose reduction in elderly population** (AUC increased by two- to threefold), debilitated patients, **and patients with reduced creatinine clearance**; acute overdose reactions include nausea, vomiting, abdominal pain, deterioration of renal function/acute renal failure (e.g., due to interstitial nephritis), rare central nervous system symptoms (dizziness, somnolence, coma, convulsions) and a drop in blood pressure (sometimes progressing to shock), as well as heart arrhythmia (tachycardia); excretion of a harmless metabolite (ribonic acid) may cause the red coloration of urine after very high doses
**Metformin** [70]	lactic acidosis, most often occurs at the acute worsening of renal function, cardiorespiratory illness, or sepsis, metformin accumulation with acute reduction in renal function increasing the risk of lactic acidosis; may reduce vitamin B12 serum levels, monitor for appropriate corrective treatment for vitamin B12 deficiency; taste disturbance; nausea, vomiting, diarrhea, abdominal pain, and loss of appetite, most frequently during the initiation of therapy, to prevent them, it is recommended to take metformin in 2 or 3 daily doses and to increase the doses slowly	drugs that can acutely impair renal function (such as antihypertensives, diuretics, and NSAIDs) should be initiated with caution in metformin-treated patients; other risk factors for lactic acidosis are excessive alcohol intake, hepatic insufficiency, inadequately controlled diabetes, ketosis, prolonged fasting, and any conditions associated with hypoxia, as well as concomitant use of medicinal products that may cause lactic acidosis, metformin alone does not cause hypoglycemia, but caution is advised when it is used in combination with insulin or other oral antidiabetics (e.g., sulfonylureas or meglitinides); alcohol intoxication is associated with an increased risk of lactic acidosis, particularly in cases of fasting or malnutrition, hepatic impairment; drugs that can adversely affect renal function may increase the risk of lactic acidosis, e.g., NSAIDs, including selective cyclo-oxygenase (COX) II inhibitors, ACE inhibitors, angiotensin II receptor antagonists, and diuretics, especially loop diuretics; drugs with intrinsic hyperglycemic ncreaity (e.g., glucocorticoids—systemic and local routes—and sympathomimetics) require more frequent blood glucose monitoring and metformin dose adjustment; metformin is a substrate of both transporters OCT1 and OCT2, inhibitors of OCT1 (such as verapamil) may reduce the efficacy of metformin; inducers of OCT1 (such as rifampicin) may increase the gastrointestinal absorption and efficacy of metformin; inhibitors of OCT2 (such as cimetidine, dolutegravir, ranolazine, trimethoprim, vandetanib, isavuconazole) may decrease the renal elimination of metformin and thus lead to an increase in metformin plasma concentration; inhibitors of both OCT1 and OCT2 (such as crizotinib, Olaparib) may alter the efficacy and renal elimination of metformin, caution especially in patients with renal impairment, when these drugs are coadministered with metformin, as the metformin plasma concentration may increase	must be discontinued at the time of surgery under general, spinal, or epidural anesthesia, therapy may be restarted no earlier than 48 h following surgery or resumption of oral nutrition and provided that renal function remained stable; intravascular administration of iodinated contrast agents may lead to contrast induced nephropathy, metformin accumulation, and an increased risk of lactic acidosis, metformin should be discontinued prior to or at the time of the imaging procedure, and should not restarted until at least 48 h after provided stable renal function; diabetic pre-coma; any type of acute metabolic acidosis (such as lactic acidosis, diabetic ketoacidosis); severe renal failure (GFR < 30 mL/min); acute conditions with the potential to alter renal function such as dehydration, severe infection, shock; disease which may cause tissue hypoxia (especially acute disease, or worsening of chronic disease), such as: decompensated heart failure, respiratory failure, recent myocardial infarction, shock; hepatic insufficiency; acute alcohol intoxication, alcoholism	in dehydration (severe diarrhea or vomiting, fever, or reduced fluid intake), metformin should be temporarily discontinued; with overdose lactic acidosis, the most effective method to remove lactate and metformin is hemodialysis
**Methotrexate** [71]	supervise for toxic signs and symptoms; increased risk of raised methotrexate blood levels and ARDs with, e.g., dehydration, impaired renal function, additional, or increased dose drugs, such as NSAIDs, administered concomitantly; hematopoietic suppression; deaths have been reported associated with the use of methotrexate in psoriasis; lung manifestations of RA and other connective tissue disorders; pleural effusions and ascites should be drained before methotrexate is started, can accumulate in these fluids and may be re-excreted into the circulation, prolonging the serum half-life, and resulting in unexpected toxicity; adequate hydration prior to and during treatment is required to limit the risk of renal toxicity; folate deficiency may increase methotrexate toxicity; systemic toxicity may follow intrathecal use; tumor lysis syndrome may occur in patients with rapidly growing tumors; If acute methotrexate toxicity occurs, patients may require folinic acid (to neutralize bone marrow effects); in patients with rheumatoid arthritis or psoriasis, folic acid or folinic acid supplementation may reduce toxicity, such as gastrointestinal symptoms, stomatitis, alopecia, and elevated liver enzymes, plasma methotrexate levels should be monitored in order to calculate the appropriate dose; check levels of vitamin B12 prior to initiating folic acid supplementation, particularly in adults aged over 50 years, as folic acid intake may mask a vitamin B12 deficiency; infections; encephalopathy/leukoencephalopathy; progressive multifocal leukoencephalopathy (PML); monitor hepatotoxicity; acute or chronic interstitial pneumonitis, often associated with blood eosinophilia, treatment resistant interstitial fibrosis, pulmonary alveolar hemorrhage; may cause renal damage that may lead to acute renal failure, monitor; diarrhea and ulcerative stomatitis; inactive chronic infections (e.g., herpes zoster, tuberculosis, hepatitis B or C) potential activation; development of malignant lymphomas; headache, dizziness, fatigue	**CsA may potentiate efficacy and toxicity**, there is a risk of excessive immunosuppression with risk of lymphoproliferation when the combination is used; **concomitant use of proton pump inhibitors (PPIs) and a high dose methotrexate should be avoided,** especially in patients with renal impairment; the concomitant administration of hepatotoxic or haematotoxin DMARDs (e.g., leflunomide) is not advisable, due to the possibility of fatal or severe toxic reactions; serious ADRs including deaths have been reported with the concomitant administration of methotrexate (usually in high doses) and nonsteroidal anti-inflammatory drugs (NSAIDs); in the treatment of rheumatoid arthritis, treatment with ASA and NSAIDs as well as small-dose steroids can be continued, but the possible increased risk of toxicity needs to be monitored; the steroid dose can be reduced gradually in patients who exhibit therapeutic response to methotrexate; the concomitant administration of folate antagonists, such as trimethoprim/sulfamethoxazole, has been reported to cause an acute megaloblastic pancytopenia; vitamin preparations containing folic acid (or its derivatives) may alter the response to methotrexate; salicylates and sulphonamides, whether antibacterial, hypoglycemic, or diuretic, should not be given, are extensively protein bound, and may displace, or be displaced by, other acidic drugs, the concurrent administration of agents, such as diphenylhydantoin, acidic anti-inflammatory agents, salicylates, phenylbutazone, phenytoin, barbiturates, tranquilizers, oral contraceptives, amidopyrine derivatives, p-aminobenzoic acid, thiazidediuretics, oral hypoglycaemics, doxorubicin, tetracyclines, probenecid, or sulfinpyrazone or oral hypoglycaemics will decrease the methotrexate transport function of renal tubules, thereby reducing excretion and almost certainly increasing methotrexate toxicity; increased risk of agranulocytosis with olanzapine; plasma concentrations of methotrexate increased by acitretin—also an increased risk of hepatotoxicity; azapropazone reduced the excretion of methotrexate; NSAIDs (aspirin, ibuprofen or indomethacin) increase and prolonged serum methotrexate concentrations with consequent increased gastrointestinal and hematological toxicity; sulfamethoxazole and folate antagonists, such as trimethoprim (as co-trimoxazole)—increased risk of hematological toxicity; probenecid and weak organic acids (e.g., loop diuretics: pyrazoles)—the excretion of methotrexate reduced (increased risk of toxicity)	not in significantly impaired hepatic function, severe/significantly impaired renal function (creatinine clearance less than 30 mL/min) for methotrexate doses <100 mg/m^2^, and moderate renal impairment (creatinine clearance less than 60 mL/min) for methotrexate doses >100 mg/m^2^, liver disease including fibrosis, cirrhosis, recent or active hepatitis, active infectious disease, pre-existing blood dyscrasias, such as bone marrow hypoplasia, significant anemia, leucopenia, or thrombocytopenia, alcoholism, severe acute or chronic infections, immunodeficiency syndrome, stomatitis, ulcers of the oral cavity and known active gastrointestinal ulcer disease, and concurrent vaccination with live vaccines; methotrexate tablets should not be used concomitantly with drugs with antifolate properties (e.g., co-trimoxazole); methotrexate is teratogenic and should not be given during pregnancy or to mothers who are breast-feeding; following administration to an adult, conception should be avoided by using an effective contraceptive method for at least 6 months after using methotrexate 2.5 mg tablets	the prescriber should specify the day of intake on the prescription once a week; patients should be instructed on the importance of adhering to the once-weekly intakes; full blood count (including hematocrit), hepatic and renal function tests (including urinalysis), the routine examination of lymph nodes, patients should report any unusual swelling; stop in acute infection; radiotherapy may increase the risk of soft tissue necrosis and osteonecrosis; radiation-induced dermatitis and sun burn can reappear under methotrexate therapy (recall reaction); psoriatic lesions may get worse if methotrexate is combined with ultraviolet radiation/PUVA
**Metoclopramide** [72]	somnolence; depression; extra-pyramidal disorders; tardive dyskinesia, potentially irreversible, especially in the elderly; neuroleptic malignant syndrome; severe bradycardia, cardiac arrest, and QTc prolongation	**increases CsA exposure by 22%; serotonergic drugs such as SSRIs may increase the risk of serotonin syndrome**	not in patients with epilepsy; Parkinson’s disease; gastrointestinal hemorrhage, mechanical ncreasetion, or gastro-intestinal perforation	**dose adjustment in renal impairment and in severe hepatic impairment**
**Midazolam** [73]	lower doses in elderly people due to higher levels sensitivity to effects of benzodiazepines; severe cardiorespiratory ADRs incl. respiratory depression, apnea, respiratory arrest, dyspnea, laryngospasm cardiac arrest, and bradycardia; when used as a premedication, adequate observation of the patient after administration is mandatory as interindividual sensitivity varies and symptoms of overdose may occur; high risk patients are as follows: adults over 60 years of age, chronically ill or debilitated patients, e.g., patients with chronic respiratory insufficiency, patients with chronic renal failure, impaired hepatic function or with impaired cardiac function, pediatric patients especially those with cardiovascular instability; caution with myasthenia gravis; tolerance; dependence; withdrawal symptoms; anterograde amnesia; **paradoxical reactions**; sedation, amnesia, impaired attention, and impaired muscular function may adversely affect the ability to drive or use machines; nausea, vomiting, constipation, dry mouth; fatigue, **falls and fracture**; hypotension, vasodilating effects, thrombophlebitis, thrombosis; greater likelihood of adverse drug reactions in patients with severe renal impairment and in elderly	elimination may be altered in patients receiving compounds that inhibit or induce CYP3A4 (azoles, etc.), and the dose of midazolam may need to be adjusted accordingly; the concomitant use of midazolam with alcohol or/and CNS depressants should be avoided; concomitant opioids may result in sedation, respiratory depression, coma, and death; avoid use in patients with a medical history of alcohol or drug abuse; pharmacokinetic interactions with CYP3A4 inhibitors or inducers are more pronounced for oral use when compared to IV midazolam, particularly since CYP3A4 also exists in the upper gastro-intestinal tract; the administration of high doses or long-term infusions of midazolam to patients receiving **strong CYP3A4inhibitors, e.g., during intensive care, may result in long-lasting hypnotic effects, delayed recovery and respiratory depression, thus requiring dose adjustments**; for strong inducers, a relevant induction even after short-term treatment cannot be excluded; increased with verapamil/diltiazem, erythromycin, clarithromycin, telithromycin, IV fentanyl, propofol, protease inhibitors, nefazodone, tyrosine kinase inhibitors, aprepitant; decreased drug exposure by inducers rifampicin, ticagrelor, carbamazepine, phenytoin, mitotane (strong), enzalutamide (strong), clobazam and efavirenz, vemurafenib, St. John’s Wort	not to be used for conscious sedation in patients with severe ncreastory failure or acute respiratory depression; protease inhibitors should not be coadministered with orally administered midazolam	hepatic impairment reduces the clearance of intravenous midazolam, reduce dose and monitor vital signs; after prolonged infusion in intensive care unit patients, the mean duration of the sedative effect in the renal failure population was considerably increased most likely due to accumulation of α-hydroxymidazolam glucuronide; no specific data in patients with severe renal impairment (creatinine clearance below 30 mL/min) receiving midazolam for induction of anesthesia; to be administered only by experienced physicians in a setting fully equipped for the monitoring and support of respiratory and cardiovascular function and by persons specifically trained in the recognition and management of expected adverse events, including respiratory and cardiac resuscitation; elimination delayed in patients with liver dysfunction, low cardiac output
**Mirtazapine** [74]	bone-marrow depression, usually presenting as granulocytopenia or agranulocytosis; jaundice; akathisia, psychomotor restlessness; cases of QTc prolongation, torsade de pointes, ventricular tachycardia, and sudden death have been reported during post-marketing use; hyponatremia, serotonin syndrome; the elderly are often more sensitive, especially with regard to the undesirable effects of antidepressants; increase in appetite and weight; abnormal dreams, confusion, anxiety, insomnia; somnolence, sedation, headache; orthostatic hypotension	the concomitant administration of serotonergic agents, such as MAO inhibitors, selective serotonin re-uptake inhibitors (SSRIs), serotonin norepinephrine re-uptake inhibitors (SNRIs) or tricyclic antidepressants, and buprenorphine-containing medicinal products, may result in serotonin syndrome, a potentially life-threatening condition; serotonergic active substances (L-tryptophan, triptans, tramadol, linezolid, methylene blue, SSRIs, venlafaxine, lithium, and St. John’s Wort) may lead to an incidence of serotonin associated effects; may increase the sedating properties of benzodiazepines and other sedatives (notably, most antipsychotics, antihistamine H1 antagonists, opioids); risk of QTC prolongation and/or ventricular arrhythmias (e.g., torsade de pointes) may be increased with QTc-prolonging antipsychotics and antibiotics; carbamazepine and phenytoin, CYP3A4 inducers, about a 2-fold increase in mirtazapine clearance; potent CYP3A4 inhibitors, such as HIV protease inhibitors, azole antifungals, erythromycin, cimetidine, or nefazodone, increase exposure; cimetidine increases exposure	not with or within two weeks of monoamine oxidase (MAO) inhibitors; caution in cardiac diseases, low blood pressure, diabetes mellitus; avoid alcohol; caution with buprenorphine for risk of serotonin syndrome	careful dosing as well as regular and close monitoring in hepatic and renal impairment; increased suicidal risk with antidepressants compared to placebo in patients less than 25 years old; close supervision of patients, in particular those at high risk, especially those in early treatment and those experiencing dose changes
**Moxonidine** [75]	headache, dizziness, somnolence, vertigo; dry mouth; diarrhea, nausea, vomiting, dyspepsia; asthenia, insomnia; back pain; **bradycardia**, syncope, hypotension (including orthostatic), varying degrees of **AV block**	not recommended with tricyclic antidepressants (reduce the effectiveness of centrally acting antihypertensives); can potentiate the sedative effect of tricyclic antidepressants (avoid co-prescribing), tranquilizers, alcohol, and hypnotics; may enhance the sedative effect of benzodiazepines; interaction with other drugs that are excreted through tubular excretion cannot be excluded; in combination with a β-blocker, for discontinuation, the β-blocker should be discontinued first, and then moxonidine after a few days (doses should be reduced gradually over a period of two weeks)	not in sick sinus syndrome, bradycardia (resting heart rate < 50 beats/min), AV-block 2nd and 3rd degree, and cardiac insufficiency	**caution with renal impairment, excreted primarily via the kidney, careful titration of the dose is recommended**; in 1^st^ degree AV block, special care should be exercised to avoid bradycardia, in severe coronary artery disease or unstable angina pectoris, special care should be exercised because of limited experience; the elderly may be more susceptible to the cardiovascular effects of antihypertensives, start low and increment dose with caution
**MMF Mycophenolate mofetil** [76,77,78,79]	**hypogammaglobulinemia**; risk of lymphomas and other malignancies, particularly of the skin; infections, latent **viral reactivation, progressive multifocal leukoencephalopathy (PML) associated with the JC virus, BK virus-associated nephropathy sometimes leading to renal graft loss**; leukopenia, anemia, thrombocytopenia and pancytopenia, pure red cell aplasia (PRCA); increased incidence of digestive system adverse events, including uncommon cases of gastrointestinal tract ulceration, hemorrhage, and perforation (colon, gall bladder); elderly patients > 65yrs may be at an increased risk of ADRs, such as certain infections (includeing CMV tissue invasive disease), possible gastrointestinal hemorrhages, and pulmonary oedema, compared with younger individuals; diarrhea, leucopenia, sepsis, and vomiting, and there is evidence of a higher frequency of certain types of infections, such as tuberculosis and atypical mycobacterial infection; uncommon but serious life-threatening infections such as meningitis and infectious endocarditis have been reported; bronchopulmonary infections; hypercholesterolemia; hypophosphatemia; headache; hypertension; cough, dyspnea; diarrhea (endoscopic investigation of patients with CellCept-related diarrhea have revealed isolated cases of intestinal villous atrophy), dyspepsia, nausea, vomiting; hematuria; oedema; pyrexia	**PPIs significantly reduce the overall exposure to MPA after oral administration of mycophenolate mofetil, this interaction does not exist between PPIs and enteric-coated mycophenolic acid (EC-MPA),** immunosuppressive effect may probably not be impaired in renal transplants; increase of approximately 20% in TAC AUC when multiple doses of CellCept (1.5 g twice daily) were administered to patients taking TAC; **CsA interferes with MPA enterohepatic recirculation, resulting in reduced MPA exposures by 30–50% in renal transplant patients**, similar to other patients devoid of this effect, e.g., belatacept, cholestyramine, or vice versa, as this might result in reduced MPA exposure; for drugs which interfere with MPA’s enterohepatic cycle, e.g., cholestyramine, antibiotics should be used with caution due to their potential to reduce the plasma levels and efficacy of MPA; **as MPAG plasma concentrations are increased in renal impairment, so are aciclovir concentrations, the potential exists for the MPA and aciclovir or its prodrugs, e.g., valaciclovir, to compete for tubular secretion and thus further increase the concentrations of both drugs**; with antacids, such as magnesium and aluminum hydroxides, MMF absorption decreased; antibiotics eliminating glucuronidase-produ-cing bacteria in the intestine (e.g., aminoglycoside, cephalosporin, fluoroquinolone, and penicillin classes of antibiotics) may interfere with MPAG/MPA enterohepatic recirculation, thus leading to reduced systemic MPA exposure; norfloxacin and metronidazole reduced the MPA AUC, drugs inhibiting the glucuronidation of MPA (isavuconazole) may increase MPA exposure; telmisartan enhances UGT1A9 expression and activity and decreases MPA; **increased with SIR**; as MPAG plasma and ganciclovir concentrations are increased in the presence of renal impairment, the potential exists for the two medicines to compete for tubular secretion, and thus further increases in concentrations of both medicines may occur; iron supplements should be administered at least 3 h following MPA; **rifampicin decreases MPA (CellCept) exposure by 70%**; sevelamer and other calcium-free phosphate binders should preferentially be given two hours after CellCept intake to minimize the impact on the reduced absorption of MPA; the renal clearance of MPAG occurs by renal tubular secretion, as well as glomerular filtration, the coadministration of probenecid, a known inhibitor of tubular secretion, with MMF raises the plasma AUC of MPAG threefold, thus, other medicines known to undergo renal tubular secretion may compete with MPAG, thereby raising the plasma concentrations of MPAG or the other drug undergoing tubular secretion	contraindicated in women with childbearing potential not using highly effective contraceptive methods, men should not donate semen during therapy and for 90 days following discontinuation; if neutropenia develops (absolute neutrophil count < 1.3 × 103/μL), dosing with CellCept should be interrupted; not with azathioprine; in severe chronic renal impairment (GFR < 25 mL/min/1.73m^2^), the administration of MPA doses greater than 1g twice daily should be avoided; with delayed graft function posttransplant, mean MPA AUC0-12 was comparable, but MPAG AUC0-12 was two- or threefold higher, monitor carefully; no live vaccines	patients should be switched from IV to oral CellCept as soon as they can tolerate oral medication; patients receiving MPA should be instructed to immediately report any evidence of infection, unexpected bruising, bleeding, or any other manifestation of bone marrow depression; vaccinations may be less effective, and the use of live attenuated vaccines should be avoided, influenza vaccination may be of value; the therapeutic drug monitoring of MPA may be appropriate when switching combination therapy (e.g., from CsA to TAC or vice versa) or to ensure adequate immunosuppression in patients with high immunological risk (e.g., risk of rejection, treatment with antibiotics, addition or removal of an interacting medication)
**Pantoprazole, Omeprazole other PPIs** [80,81,82,83,84]	**reduced absorption of vitamin B12** due to hypo- or achlorhydria; **increased risk of gastrointestinal infections caused by bacteria such as *Salmonella* and *Campylobacter* or *C. difficile*; hypomagnesemia with serious manifestations,** such as fatigue, tetany, delirium, convulsions, dizziness, and ventricular arrhythmia; risk of hip, wrist, and spine fracture, predominantly in the elderly; rare **cases of agranulocytosis, very rare thrombocytopenia, leukopenia, pancytopenia**; benign fundic gland polyps; headache, dizziness, sleep disorders; diarrhea, nausea, vomiting, abdominal distension, and bloating, constipation, dry mouth, abdominal pain and discomfort; increase in hepatobiliary enzymes; asthenia, fatigue, and malaise	may interfere with the absorption of other medicinal products where gastric pH is an important determinant of oral bioavailability, e.g., some azole antifungals, such as ketoconazole, itraconazole, posaconazole, and other medicines like erlotinib, HIV protease inhibitor may need to be adjusted; with warfarin or phenprocoumon increases in INR and prothrombin time; increased levels of high dose methotrexate (e.g., 300 mg) necessitate consideration of PPI withdrawal; inhibitors of CYP2C19 such as fluvoxamine could increase the systemic exposure of pantoprazole, a dose reduction may be considered for patients treated long-term with high doses of pantoprazole, or those with hepatic impairment; CYP2C19 and CYP3A4 inducers, such as rifampicin and St. John´s wort, may decrease the PPI plasma concentrations	in severe hepatic impairment max. 20 pantoprazole mg/d, in the case of a rise in liver enzymes, discontinue use; not recommended with HIV protease inhibitors for which absorption is dependent on acidic intragastric pH, such as atazanavir	should be swallowed whole 1 h before a meal; **tube feeding, PEG: administer omeprazole (pantoprazole not possible)**; in long-term treatment, especially >1 year, patients should be kept under regular surveillance; differentiate prophylactic versus therapeutic doses (e.g. pantoprazol 20 mg versus ≥40 mg/per day); deprescribing with biweekly dose halving to prevent risk of rebound; lowering the PPI dose (e.g., from twice to once daily, or halving the dose, or taking every second day) or stopping the PPI and using it on-demand are equally recommended strong options according to the algorithm by Farrell et al.
**Paracetamol, (Acetaminophen)** [85,86,87]	overdose: **liver damage is possible in adults who have taken 10 g or more of paracetamol, ingestion of 5 g or more of paracetamol may lead to liver damage if the patient has risk factors** (see below): risk factors long term treatment with carbamazepine, phenobarbital, phenytoin, primidone, rifampicin, St. John’s Wort, or other drugs that induce liver enzymes, or the regular consumption of ethanol in excess amounts, or likely to be glutathione deplete, e.g., eating disorders, cystic fibrosis, HIV infection, starvation, cachexia; blood dyscrasias, including thrombocytopenia and agranulocytosis	the anticoagulant effect of warfarin and other coumarins may be enhanced by the prolonged regular daily use of paracetamol with an increased risk of bleeding; occasional doses have no significant effect; caution should be taken when paracetamol is used concomitantly with flucloxacillin, as concurrent intake has been associated with high anion gap metabolic acidosis, especially in patients with risks factors	2 tablets (500 mg) every 4 h (German version: 6 h) as required, not more than eight tablets (4 g) in 24 h, do not take for more than 3 days without consulting; these doses should not be given more frequently than every four hours, nor should more than four doses be given in any 24 h period; symptoms of paracetamol overdosage, in the first 24 h, are pallor, nausea, vomiting, anorexia, and abdominal pain; liver damage may become apparent 12 to 48 h after ingestion; abnormalities of glucose metabolism and metabolic acidosis may occur; in severe poisoning, hepatic failure may progress to encephalopathy, disseminated intravascular coagulation, hemorrhage, hypoglycemia, cerebral oedema, gastrointestinal bleeding, and death; acute renal failure with acute tubular necrosis, strongly suggested by loin pain, hematuria, and proteinuria may develop, even in the absence of severe liver damage; cardiac arrhythmias and pancreatitis have been reported; antidot: N-acetylcysteine may be used up to 24 h after the ingestion of paracetamol; however, the maximum protective effect is obtained up to 8 h post-ingestion	**intoxications with paracetamol (APAP) are the second most common causes of liver transplants worldwide** [87]—**the maximum permitted daily dose over 3 days in >43 kg adolescents (from 12 years) and adults max 4 × 1 g; the minimal time intervals between each dose differs comparing SmPCs: 4 h for the USA version versus 6h for the German version; reduce dose and care in patients with renal or severe hepatic impairment**; the hazard of overdose is greater in those with non-cirrhotic alcoholic liver disease; do not exceed the recommended dose; do not take for more than 3 days without consulting; do not take with any other paracetmol-contai-ning products; keep out of the reach of children; caution if administered concomitantly with flucloxacillin, due to the increased risk of high anion gap metabolic acidosis (HAGMA), particularly in patients with severe renal impairment, sepsis, malnutrition, and other sources of glutathione deficiency (e.g., chronic alcoholism), as well as those using maximum daily doses of paracetamol, close monitoring, including measurement of urinary 5-oxoproline, is recommended; immediate medical advice should be sought in the event of an overdose, even if one feels well, because of the risk of delayed, serious liver damage
**Parecoxib** [88]	COX-2 inhibitors have been associated with the increased **risk of cardiovascular and thrombotic ADRs** when taken long term, caution in patients with significant risk factors for cardiovascular events (e.g., hypertension, hyperlipidemia, diabetes mellitus, smoking); aggravation of soft tissue infections; **upper gastrointestinal (GI) complications (perforations, ulcers or bleedings [PUBs]), higher risk in elderly**, or patients with a prior history of gastrointestinal disease, such as ulceration and GI bleeding, or patients using **ASA concomitantly, anticoagulants, such as warfarin/coumarin-type and DOACs, glucocorticoids, SSRIs/SNPIs, antiplatelet drugs, other NSAIDs, or patients ingesting alcohol;** severe skin reactions; fluid retention, oedema, oliguria, renal impairment; pharyngitis, alveolar osteitis; anemia postoperative; hypokalemia; agitation, insomnia; hypoesthesia, dizziness; hypertension, hypotension; respiratory insufficiency, bronchospasm; abdominal pain, vomiting, constipation, dyspepsia, flatulence; hepatitis probably; significant greater incidence of cardiovascular/thromboem-bolic events	**increased nephrotoxicity with CsA, TAC;** diminishes the antihypertensive effect of ACE inhibitors, angiotensin II antagonists, beta-blockers, and diuretics; in elderly patients, volume-depleted (including those on diuretic therapy), or with compromised renal function, the coadministration of NSAIDs, including selective COX-2 inhibitors, with ACE inhibitors or angiotensin-II antagonists, may result in the further deterioration of renal function, including possible acute renal failure; fluconazole increases the exposure and requires dose reduction; inhibits CYP2D6 and thus enhances the exposure of, e.g., flecainide, propafenone, metoprolol; inhibits CYP2C19, and thus may enhance the exposure of, e.g., phenytoin, diazepam, imipramine, omeprazole; may result in increased plasma levels of methotrexate; significant decreases in lithium serum clearance; may delay or prevent rupture of ovarian follicles, which has been associated with reversible infertility in some women	**not in severe hepatic impairment; caution needed for lowest dose and monitoring in severe renal impairment**; previous serious allergic drug reaction of any type; active peptic ulceration or GI bleeding; patients who have experienced bronchospasm, acute rhinitis, nasal polyps, angioneurotic oedema, urticaria, or other allergic-type reactions after taking ASA or NSAIDs, including COX-2 inhibitors; inflammatory bowel disease; congestive heart failure (NYHA II-IV); treatment of post-operative pain following coronary artery bypass graft (CABG) surgery; established ischemic heart disease, peripheral arterial disease, and/or cerebrovascular disease	may mask fever and other signs of inflammation; caution in patients with compromised cardiac function, preexisting oedema, or other conditions predisposing to, or worsened by, fluid retention, including those taking diuretic treatment or otherwise at risk of hypovolemia; in healthy elderly subjects, the apparent oral clearance of valdecoxib (activated parecoxib) was reduced, resulting in an approximately 40% higher plasma exposure to valdecoxib
**Posaconazole** [89,90]	hepatic toxicity—ALT, AST, bilirubin alkaline phosphatase, GGT increased; nausea, vomiting, diarrhea (may overlap/mimic symptoms of CMV, GvHD)—patients with severe diarrhea or vomiting should be monitored closely for breakthrough fungal infections; thrombotic thrombocytopenia purpura; hemolytic uremic syndrome; QTc prolongation and torsade de pointes—administer with caution to patients with pro-arrhythmic conditions, such as congenital or acquired QTc prolongation, cardiomyopathy, especially in the presence of cardiac failure, sinus bradycardia, existing symptomatic arrhythmias, concomitant use with medicinal products known to prolong the QTc interval, electrolyte disturbances, especially those involving potassium, magnesium, or calcium levels, should be monitored and corrected as necessary before and during posaconazole therapy; agranulocytosis, aplastic anemia; depression; adrenal insufficiency; convulsion; hypertension; hypokalemia; photopsia, visual brightness, visual disturbances; paresthesia, dizziness, somnolence, headache, dysgeusia; pyrexia, asthenia, fatigue	potent inhibitor of CYP3A4, coadministration with CYP3A4 substrates may result in large increases in exposure, e.g., CsA (nephrotoxicity and fatal cases of leukoencephalopathy), **dose of CsA should be reduced (e.g., to about three quarters of the current dose), TAC (dose of TAC should be reduced, e.g., to about one third of the current dose), SIR (not to combine), EVR (not recommended)**, atazanavir, simvastatin, atorvastatin, lovastatin; venetoclax; prolonged sedation and possible respiratory depression, **coadministration with any benzodiazepines metabolized by CYP3A4 (e.g., midazolam, triazolam, alprazolam)**—**avoid or reduce dose**; caution vincristine, vinblastine, and venetoclax toxicity; significantly lowered posaconazole concentrations with rifampicin, rifabutin, phenytoin, carbamazepine, phenobarbital, primidone, efavirenz, fosamprenavir—avoid combination; posaconazole is metabolized via UDP glucuronidation, and is a substrate for P-gp efflux, **inhibitors, e.g., verapamil, CsA, quinidine, clarithromycin, erythromycin increase posaconazole plasma concentrations**; increased HIV protease inhibitors; diltiazem, verapamil, nifedipine, nisoldipine, felodipine, lercanidipine increase, dose adjustment	no coadministration with ergot alkaloids with CYP3A4 substrates terfenadine, astemizole, cisapride, pimozide, halofantrine, or quinidine with increased plasma concentrations, QTc prolongation, or the risk of torsades de pointes; coadministration with the HMG-CoA reductase inhibitors simvastatin, lovastatin, and atorvastatin; coadministration during the initiation and dose-titration phase of venetoclax in chronic lymphocytic leukemia (CLL) patients; increased ergot alkaloids; **simvastatin, lovastatin, and atorvastatin must be discontinued for the risk of rhabdomyolysis**; rifabutin increased; **not with SIR for 8-fold AUC increase**	Caution: with hepatic impairment increased plasma exposure; tablets should be swallowed whole; posaconazole tablets and oral suspension are not interchangeable, plasma concentrations following the administration of posaconazole tablets are generally higher than those obtained with posaconazole oral suspension; posaconazole plasma concentrations following the administration of posaconazole tablets may increase over time in some patients; posaconazole is an inhibitor of CYP3A4
**Pravastatin** [91]	arthralgia, muscle cramps, myalgia, muscle weakness, and elevated CK levels; elevations of serum transaminases; peripheral polyneuropathy is very rare, particularly if used for long periods of time, as are paresthesia, pancreatitis, jaundice, hepatitis, fulminant hepatic necrosis, rhabdomyolysis, which can be associated with acute renal failure, secondary to myoglobinuria, myopathy, myositis, polymyositis, tendon disorders, which may induce de novo or aggravate pre-existing myasthenia gravis or ocular myasthenia; immune-mediated necrotizing myopathy; photosensitivity reaction; class effects: nightmares, memory loss, depression, exceptional cases of interstitial lung disease, especially with long term therapy, diabetes mellitus; interstitial lung disease have been reported with some statins (presenting features can include dyspnea, a non-productive cough, and a deterioration in general health (fatigue, weight loss, and fever)	**CsA leads to an approximately 4-fold increase in pravastatin systemic exposure**, in some patients, however, the increase in pravastatin exposure may be larger; the risk and severity of muscular disorders during statin therapy is increased via the coadministration of interacting medicines, such as **CsA**, clarithromycin, and other macrolides or niacin, **an increase in the incidence of myopathy has also been described in patients receiving other statins in combination with inhibitors of cytochrome P450 metabolism, this may result from pharmacokinetic interactions that have not been documented for pravastatin;** caution with colchicine (rhabdomyolysis); increased exposure of vitamin K antagonists requires the appropriate monitoring of INR is needed; increased statin exposure, cautiously with macrolide antibiotics (e.g., erythromycin, clarithromycin, roxithromycin); not metabolized to a clinically significant extent by the cytochrome P450 system, in contrast to other statins; the absence of a significant pharmacokinetic interaction with pravastatin has been specifically demonstrated for several products, particularly those that are substrates/inhibitors of CYP3A4, e.g., diltiazem, verapamil, itraconazole, ketoconazole, protease inhibitors, grapefruit juice, and CYP2C9 inhibitors (e.g., fluconazole); rifampicin may decrease the oral bioavailability of pravastatin; but also, with rifampicin, an increase in pravastatin AUC and Cmax was observed, caution if both are given at the same time, no interaction is expected if their dosing is administered at least two hours apart; increased risk of rhabdomyolysis with lenalidomide	not in active liver disease, including unexplained persistent elevations of serum transaminase elevation exceeding 3 × the upper limit of normal (ULN); as for other HMG-CoA reductase inhibitors, the combination of pravastatin with fibrates is not recommended; statins, including pravastatin, must not be coadministered with systemic formulations of fusidic acid or within 7 days of stopping fusidic acid; patients should be advised to report promptly unexplained muscle pain, tenderness, weakness, or cramps, in these cases, the CK levels should be measured	predisposing factors including advanced age (>65), uncontrolled hypothyroidism, and renal impairment may increase the risk of muscular toxicity, and can therefore justify a careful evaluation of the benefit/risk and special clinical monitoring, CK measurement is indicated before starting statin therapy in these patients; predisposing factors such as renal impairment, hypothyroidism, previous history of muscular toxicity with a statin or fibrate, personal or familial history of hereditary muscular disorders, or alcohol abuse, in these cases, CK levels should be measured prior to initiation of therapy; CK measurement should also be considered before starting treatment in persons over 70 years of age, especially in the presence of other predisposing factors in this population
**Quetiapine** [92]	weight gain; hyperlipidemia; hyperglycemia; tardive dyskinesia; and neuroleptic malignant syndrome; mean plasma clearance is reduced by 30% to 50% in the elderly; high α1 adrenergic receptor blocking activity may induce orthostatic hypotension, tachycardia, dizziness, and sometimes syncope, which may lead to falls; cardiomyopathy and myocarditis; myelosuppression; venous thromboembolism; hepatic, biliary, pancreatic risks; rhabdomyolysis; neuroleptic malignant syndrome; EPS, tardive dyskinesia; dysphagia; QTC prolongation; dry mouth; constipation; cognitive and motor impairment; rhabdomyolysis; hepatic failure	**CsA, TAC, and SIR enhance exposure (less vice versa); inhibitors of CYP3A4, e.g., ketoconazole, increase the exposure by up to 6.2-fold** (cave: azole antifungals, macrolide antibiotics, protease inhibitors, amiodarone, diltiazem, verapamil, nefazodone, and grapefruit juice); QTc prolongation with concomitant neuroleptics (some antibiotics, azoles, etc.), especially for patients with an increased risk of QTc prolongation, i.e., the elderly, patients with congenital long QTc syndrome, congestive heart failure, heart hypertrophy, hypokalemia, or hypomagnesemia; urinary retention with other anticholinergic drugs; CYP3A4 inducers, such as carbamazepine and phenytoin, reduce exposure	use for the shortest time that is clinically indicated; not indicated in elderly patients with dementia—increased risk of death	**reduce dose in mild hepatic impairment; no data on renal impairment dosing, caution**; drowsiness and sedation, tachycardia, hypotension, and anticholinergic effects if overdosed; caution in patients with known cardiovascular disease, cerebrovascular disease, or other conditions predisposing to hypotension, e.g., dehydration, hypovolemia, and treatment with antihypertensive medications
**Remdesivir** [93]	transaminase elevations; severe renal toxicity was observed (not to be used in patients with eGFR < 30 mL/min); headache, nausea, rash	risk of reduced antiviral activity when coadministered with chloroquine or hydroxychloroquine (antagonistic effect); potential of the interaction of remdesivir with inhibitors/inducers of the hydrolytic pathway (esterase) or CYP2C8, 2D6, or 3A4 has not been studied, the risk of clinically relevant interaction is unknown, strong inhibitors may result in increased remdesivir exposure; the use of strong inducers (e.g., rifampicin) may decrease plasma concentrations of remdesivir and is not recommended; may transiently increase plasma concentrations of substrates of CYP3A or OATP 1B1/1B3, no data is available, however, it can be suggested that medicinal products that are substrates of CYP3A4 or substrates of OATP 1B1/1B3 should be administered at least 2 h after remdesivir; induced CYP1A2 and potentially CYP3A in vitro, coadministration of remdesivir with CYP1A2 or CYP3A4 substrates with narrow therapeutic index may lead to the loss of their efficacy	remdesivir should not be initiated in patients with ALT ≥ five times the upper limit of normal at baseline; contains betadex sulfobutyl ether sodium, which is renally cleared and accumulates in patients with decreased renal function, which may potentially adversely affect renal function and therefore should not be used in patients with eGFR < 30 mL/min	this has not been evaluated in patients with renal impairment, patients with eGFR ≥ 30 mL/min have received remdesivir for the treatment of COVID-19 with no dose adjustment, remdesivir should not be used in patients with eGFR < 30 mL/min; pharmacokinetics of remdesivir have not been evaluated in patients with hepatic impairment, it is not known if a dosage adjustment is appropriate in patients with hepatic impairment
**Rifampicin** [94]	monitor liver function; severe, systemic hypersensitivity reactions; enhanced metabolism of endogenous substrates including adrenal hormones, thyroid hormones and vitamin D; severe skin reactions, preceded by fever, lymphadenopathy, or biological abnormalities (including eosinophilia, liver abnormalities) discoloration (yellow, orange, red, brown) of the teeth, urine, sweat, sputum, and tears; thrombocytopenia, leukopenia, disseminated intravascular coagulation, eosinophilia, agranulocytosis, hemolytic anemia, Vitamin K dependent coagulation disorders; anaphylactic reaction; adrenal insufficiency; decreased appetite; psychotic disorder; headache, dizziness; cerebral hemorrhage and fatalities have been reported when rifampicin administration has been continued or resumed after the appearance of purpura; shock, flushing, vasculitis, bleeding; dyspnea, wheezing, sputum discolored; nausea, vomiting, diarrhea, tooth discoloration (which may be permanent); hepatitis, hyperbilirubinemia; erythema multiforme, including Stevens–Johnson syndrome (SJS), toxic epidermal necrolysis (TEN), drug reaction with eosinophilia and systemic symptoms (DRESS) syndrome, acute generalized exanthematous pustulosis (AGEP), skin reactions, sweat discoloration; muscle weakness, myopathy, bone pain; acute kidney injury, usually due to renal tubular necrosis or tubulointerstitial nephritis; post-partum hemorrhage; menstrual disorder; edema; blood bilirubin increased, aspartate aminotransferase increased, alanine aminotransferase increased; blood pressure decreased; blood creatinine increased	with halothane (should be avoided) or isoniazid, the potential for hepatotoxicity is increased; vitamin K-dependent coagulation and severe bleeding (supplement in vitamin K deficiency, hypoprothrombinemia); with other antibiotics causing vitamin K-dependent coagulopathy, such as cefazolin (or other cephalosporins with N-methylthiotetrazole side chain) should be avoided; **rifampicin capsules may accelerate the metabolism and reduce the activity of certain coadministered drugs, and have the potential to perpetuate clinically important drug–drug interactions in order to maintain optimum therapeutic blood levels, dosages of drugs may require adjustment when starting or stopping concomitantly administered rifampicin capsules**: antiarrhythmics (e.g., disopyramide, mexiletine, quinidine, propafenone, tocainide), antiepileptics (e.g., phenytoin), hormone antagonist (antiestrogens, e.g., tamoxifen, toremifene, gestinone), antipsychotics (e.g., haloperidol, aripiprazole), anticoagulants (e.g., coumarins, DOACs, antifungals (e.g., fluconazole, itraconazole, ketoconazole, voriconazole), antivirals (e.g., saquinavir, indinavir, efavirenz, amprenavir, nelfinavir, atazanavir, lopinavir, nevirapine), barbiturate, beta-blockers (e.g., bisoprolol, propranolol), anxiolytics and hypnotics (e.g., diazepam, benzodiazepines, zopiclone, zolpidem), calcium channel blockers (e.g., diltiazem, nifedipine, verapamil, nimodipine, isradipine, nicardipine, nisoldipine), antibacterials (e.g., chloramphenicol, clarithromycin, dapsone, doxycycline, fluoroquinolones, telithromycin), corticosteroids, cardiac glycosides (digitoxin, digoxin), clofibrate, systemic hormonal contraceptives, including estrogens and progestogens, antidiabetic (e.g., chlorpropamide, tolbutamide, sulfonylureas, rosiglitazone), immunosuppressive agents **CsA, TAC, SIR, EVR,** irinotecan, thyroid hormone, losartan, analgesics (e.g., methadone, narcotic analgesics), praziquantel, quinine, riluzole, selective 5-HT3 receptor antagonists (e.g., ondansetron), statins metabolized by CYP 3A4 (e.g., simvastatin), theophylline, tricyclic antidepressants (e.g., amitriptyline, nortriptyline), cytotoxics (e.g., imatinib), diuretics (e.g., eplerenone); enalapril: decrease enalapril active metabolite exposure; dosage adjustments should be made if indicated by the patient’s clinical condition, ncreastis-C antiviral drugs (e.g., daclatasvir, simeprevir, sofosbuvir, telaprevir), concurrent use of treatment of hepatitis-C antiviral drugs and rifampicin should be avoided; morphine: plasma concentration and the analgesic effect of morphine may be reduced by rifampicin; clopidogrel increases active metabolite exposure, rifampicin strongly induces CYP2C19, resulting in both an increased level of clopidogrel active metabolite and platelet inhibition, which in particular might potentiate the risk of bleeding; as a precaution, the concomitant use of clopidogrel and rifampicin should be discouraged; rifampicin treatment reduces the systemic exposure of oral contraceptives; also, diabetes may become more difficult to control; the concurrent use of ketoconazole and rifampicin has resulted in the decreased serum concentrations of both drugs; if p-aminosalicylic acid and rifampicin are both included in the treatment regimen, they should be given not less than eight hours apart to ensure satisfactory blood levels; the concomitant antacid administration may reduce the absorption of rifampicin, daily doses of rifampicin should be given at least 1 h before the antacids; taken concomitantly, decreased concentrations of atovaquone and increased concentrations of rifampicin were observed	not with the combination of saquinavir/ritonavir (hepatotoxicity)	caution in case of renal impairment if dose >600 mg/day; paradoxical drug reaction, after the initial improvement of tuberculosis under therapy with rifampicin capsules, the symptoms may worsen again; therapeutic levels of rifampicin have been shown to inhibit standard microbiological assays for serum folate and Vitamin B12; transient elevation of BSP and serum bilirubin has been reported, rifampicin may impair biliary the excretion of contrast media used for the visualization of the gallbladder, due to the competition for biliary excretion, therefore, these tests should be performed before the morning dose of rifampicin
**Rifaximin** [95]	*Clostridium difficile*-associated diarrhea (CDAD-reported with the use of nearly all antibacterial agents); reddish discoloration of the urine; anemia; anorexia, hyperkalemia; depression, confusional state, anxiety, hypersomnia, insomnia; dizziness, headache, balance disorders, amnesia, convulsion, attention disorders, hypoesthesia, memory impairment; dyspnea, pleural effusion; upper abdominal pain, abdominal distension, diarrhea, nausea, vomiting, ascites, esophageal varices hemorrhage, dry mouth; rashes, pruritus; muscle spasms, arthralgia, myalgia; dysuria, pollakiuria, proteinuria; oedema; pyrexia; fall	in healthy subjects, clinical drug interaction studies demonstrated that rifaximin did not significantly affect the pharmacokinetics of CYP3A4 substrates; however, in hepatic impaired patients, it cannot be excluded that rifaximin may decrease the exposure of the concomitant CYP3A4 substrates administered (e.g., warfarin—monitor INR, antiepileptics, antiarrhythmics, oral contraceptives), due to the higher systemic exposure with respect to healthy subjects; not to combine with other rifamycin; it is unknown whether concomitant drugs which inhibit P-gp and/or CYP3A4 can increase the systemic exposure of rifaximin, the coadministration of a single dose **CsA** (600 mg), a potent P-glycoprotein inhibitor, with a single dose of rifaximin (550 mg) **resulted in 83-fold and 124-fold increases in rifaximin mean Cmax and AUC∞,** clinical significance unknown	not in cases of intestinal obstruction	caution in patients with severe (Child–Pugh C) hepatic impairment and in patients with MELD (Model for End-Stage Liver Disease) score > 25
**Rosuvastatin** [96]	diabetes mellitus; thrombocytopenia; headache, dizziness; constipation, nausea, abdominal pain, pancreatits; myalgia, myopathy (including myositis), rhabdomyolysis, Lupus-like syndrome, muscle rupture; asthenia; polyneuropathy, memory loss; depression, peripheral neuropathy; sleep disturbances (including insomnia and nightmares), myasthenia gravis; as with other HMG-CoA reductase inhibitors, a dose-related increase in transaminases has been observed; proteinuria; interstitial lung disease	concomitant protease inhibitor use may strongly increase rosuvastatin exposure; gemfibrozil, fenofibrate, other fibrates and lipid-lowering doses (>or equal to 1 g/day) of niacin (nicotinic acid) increase the risk of myopathy; low increase by ezetimibe; decreased with erythromycin (via increase in gut motility); ticagrelor might affect the renal excretion of rosuvastatin, increasing the risk for rosuvastatin accumulation, concomitant use of ticagrelor and rosuvastatin led to renal function decrease, increased CPK level, and rhabdomyolysis; rosuvastatin is neither an inhibitor nor an inducer of cytochrome P450 isoenzymes; therefore, drug interactions resulting from cytochrome P450-mediated metabolism are not expected; rosuvastatin is a poor substrate for these isoenzymes, no clinically relevant interactions have been observed between rosuvastatin and either fluconazole (an inhibitor of CYP2C9 and CYP3A4) or ketoconazole (an inhibitor of CYP2A6 and CYP3A4), febuxo-stat, clopidogrel, gemfibrozil increase exposure (2-fold)	not in patients with active liver disease including unexplained, persistent elevations of serum transaminases and any serum transaminase elevation, exceeding three times the upper limit of normal; not with severe renal impairment (creatinine clearance < 30 mL/min), not with myopathy, the concomitant combination of sofosbuvir/velpatasvir/voxilaprevir, **not with concomitant CsA;** a 40 mg dose is contraindicated in patients fibrates (start with 5 mg dose) or with pre-disposing factors for myopathy/rhabdomyolysis, such as moderate renal impairment (creatinine clearance < 60 mL/min), hypothyroidism, personal or family history of hereditary muscular disorders, previous history of muscular toxicity with another HMG-CoA reductase inhibitor or fibrate, alcohol abuse, situations where an increase in plasma levels may occur, Asian patients, or the concomitant use of fibrates	
**Ruxolitinib** [97]	myelosuppression, treatment should be discontinued in patients with platelet count less than 50,000/mm^3^ or absolute neutrophil count less than 500/mm^3^; serious bacterial, mycobacterial, fungal, viral and other opportunistic infections; sepsis; hepatitis B viral load (HBV-DNA titer) increases; herpes zoster; progressive multifocal leukoencephalopathy (PML); non-melanoma skin cancer; lipid abnormalities/elevations; bleeding (gastrointestinal, intracranial, epistaxis, post-procedural hemorrhage and hematuria); bruising; hyperlipidemia; dizziness, headache; elevated lipase, constipation, flatulence; elevated transaminases; hypertension	**when administering ruxolitinib with strong CYP3A4 inhibitors (such as, but not limited to, boceprevir, clarithromycin, indinavir, itraconazole, ketoconazole, lopinavir/ritonavir, ritonavir, mibefradil, nefazodone, nelfinavir, posaconazole, saquinavir, telaprevir, telithromycin, voriconazole) in MF (myelofibrosis) and PV (polycythemia vera) patients or dual inhibitors of CYP3A4 and CYP2C9 enzymes (e.g., fluconazole) in MF, PV, and GvHD patients, the unit dose must be reduced by approximately 50%, administered twice daily; 50% dose reduction with dual inhibitors of CYP2C9 and CYP3A4 enzymes (e.g., fluconazole); avoid the concomitant use of ruxolitinib with fluconazole doses greater than 200 mg daily**; with CYP3A4 inducers (such as, but not limited to, avasimibe, carbamazepine, phenobarbital, phenytoin, rifabutin, rifampin (rifampicin), St. John’s wort), an increase of the ruxolitinib dose may be needed (effects not very strong); no dose adjustment is recommended when coadministered with mild or moderate CYP3A4 inhibitors (e.g., erythromycin); however, patients should be closely monitored for cytopenia when initiating therapy with a moderate CYP3A4 inhibitor; inhibits P-glycoprotein and breast cancer resistance protein (BCRP) in the intestine with **elevated exposure** of dabigatran etexilate, **CsA**, rosuvastatin, and potentially digoxin, TDM, or the clinical monitoring of the affected substance is advised, possibly the potential inhibition of P-gp and BCRP in the intestine can be minimized if the time between administrations is kept apart as long as possible		starting doses should be reduced in patients with severe renal impairment; for patients with end-stage renal disease on hemodialysis, the starting dose should be based on platelet counts for MF patients; the starting dose should be reduced by approximately 50% in MF and PV patients with hepatic impairment; in GvHD patients with hepatic impairment not related to GvHD, the starting dose should be reduced by approximately 50%
**Sertraline** [98,99]	hemorrhage—caution in concomitant use with drugs known to affect platelet function incl. atypical antipsychotics and phenothiazines, most tricyclic antidepressants, aspirin, NSAIDs, in thrombocytopenia, patients with a history of bleeding disorders; nausea, diarrhea, anorexia, dyspepsia, tremor, seizures, dizziness, insomnia, somnolence, hallucinations, aggressive reaction, agitation, anxiety, psychosis, depersonalisation, increased sweating, dry mouth, urinary retention, sexual dysfunction; hyponatremia, SIADH; postural hypotension; serotonin syndrome; pharyngitis; mydriasis; palpitation, tachycardia; arthralgia, myalgia; chest pain	increase in plasma levels of the tricyclic antidepressants (less than some other SSRIs); strong and moderately strong CYP3A4 inhibitors, e.g., protease inhibitors, ketoconazole, itraconazole, posaconazole, voriconazole, clarithromycin, telithromycin and nefazodone, aprepitant, erythromycin, fluconazole, verapamil, and diltiazem greater increase following sertraline exposure; increased exposure of CYP2D6 substrates, e.g., propafenone and flecainide, tricyclic antidepressive and typical psychotropic drugs, metoprolol, nebivolol; QTc prolongation may result in additive effects and an increased risk of ventricular arrhythmias, including torsade de pointes and sudden death with QTc prolongating drugs, e.g., voriconazole	not in severe hepatic dysfunction; not with MAOIs (and relapse phase of 14 days required); linezolid; pimozide; serotonergic potentiation with St. John’s wort, tramadol, sumatriptan, fenfluramine; unstable epilepsy; not with grapefruit juice; **not with strong CYP3A4 inhibitors**; alcohol	**caution in patients with renal and hepatic impairment**; 98% bound to plasma proteins; **risk of serotonin syndrome with CsA**
**Simvastatin** [100,101,102]	myopathy manifested as muscle pain, tenderness, or weakness with increased CK; rhabdomyolysis; in pre-disposing factors for rhabdomyolysis, CK level measurement before starting a treatment in elderly patient >70 years, renal impairment, uncontrolled hypothyroidism, personal or familial history of hereditary muscular disorders, previous history of muscular toxicity with a statin or fibrate, alcohol abuse; myasthenia gravis; myopathy sometimes takes the form of rhabdomyolysis, with or without acute renal failure, secondary to myoglobinuria; diabetes mellitus; interstitial lung disease	**risk of rhabdomyolysis is increased with CsA, TAC, SIR, EVR**, erythromycin, clarithromycin, fluconazole, itraconazole, ketoconazole, posaconazole, voriconazole, nefazodone, niacin, gemfibrozil, other fibric acid derivates or HIV-protease inhibitors, grapefruit juice, or amiodarone; oral contraceptives increase in plasma concentrations of norethindrone and ethinyl estradiol; with daptomycin risk of myopathy and/or rhabdomyolysis; daily ticagrelor doses greater than 40 mg simvastatin are not recommended; other fibrates (except fenofibrate) do not exceed 10 mg simvastatin daily nicotinic acid ≥ 1 g/day for Asian patients, not recommended with simvastatin; with amiodarone, amlodipine, verapamil, diltiazem, elbasvir, grazoprevir, do not exceed 20 mg simvastatin daily; rifampicin is a potent CYP3A4 inducer with a loss of efficacy of simvastatin	not in active liver disease or unexplained persistent elevations of serum transaminases; **potent CYP3A4 inhibitors, that increase the AUC by at least approx. fivefold**, e.g., itraconazole, ketoconazole, posaconazole, voriconazole, HIV protease inhibitors, ritonavir, erythromycin, clarithromycin, telithromycin, **CsA**, gemfibrozil, danazol, cobicistat, and nefazodone; fusidic acid; grapefruit juice; with ticagrelor or lomitapide max. 40 mg/d	**all patients starting therapy with simvastatin, or whose dose of simvastatin is being increased, should be advised of the risk of myopathy and told to report promptly any unexplained muscle pain, tenderness, or weakness**; simvastatin should be temporarily stopped a few days prior to elective major surgery, and when any major medical or surgical condition, supervene; in patients with severe renal impairment (creatinine clearance < 30 mL/min), dosages above 10 mg/day should be carefully considered; **pitavastatin and pravastatin may be safer alternatives, since they are not metabolized by CYP3A4 or fluvastatin (only partially via CYP3A)**
**Sirolimus (SIR)** [3,4,5,6,7]	**impaired or delayed wound healing** in patients receiving SIR, including lymphocele in renal transplant patients and wound dehiscence, patients with a BMI greater than 30 kg/m^2^ may be at increased risk of abnormal wound healing; **fluid accumulation, includeing peripheral oedema, lymphoedema, pleural effusion, and pericardial effusions**; **hepatotoxicity** increasing SIR exposure; **proteinuria**; arthralgia; microbial infections; thrombocytopenia; anemia; leucopenia; hypokalemia; hypophosphatemia; hyperlipidemia with increased serum cholesterol and triglycerides that may require treatment, risk/benefit to be considered in patients with established hyperlipidemia, and to be re-evaluated in patients with severe refractory hyperlipidemia.; hyperglycemia; diabetes mellitus; ovarian cysts; thrombosis and pulmonary embolism; skin cancer; lymphoma; monitor for elevated lipids; increased susceptibility to infection, and the possible development of lymphoma and other malignancies, particularly of the skin; increases susceptibility to infection, including opportunistic infections (bacterial, fungal, viral, and protozoal), fatal infections, and sepsis, in renal transplant patients BK virus-associated nephropathy and JC virus-associated progressive multifocal leukoencephalopathy (PML) to be considered in differential diagnosis in immunosuppressed patients with deteriorating renal function or neurological symptoms; antimicrobial prophylaxis for Pneumocystis carinii pneumonia should be administered for the first 12 months following transplantation, CMV prophylaxis is recommended for 3 months after renal transplantation, particularly for patients at increased risk of CMV disease	extensively metabolized by the CYP3A4 isozyme in the intestinal wall and liver, also a substrate for the multidrug efflux pump P-gp located in the small intestine; **coadministration with voriconazole increases SIR 7-11-fold, with ketoconazole not recommended; avoid grapefruit juice; rifampicin decreases SIR (5,5-fold increased clearance of SIR) and is not recommended; renal function to be monitored with CsA**; the concomitant use of SIR with a CNI may increase the risk of CNI-induced hemolytic uremic syndrome/thrombotic thrombocytopenic purpura/thrombotic microangiopathy (HUS/TTP/TMA); **monitor for rhabdomyolysis with statins**; with (ACE) inhibitors angioneurotic oedema-type reactions, elevated SIR levels, for example, due to interaction with strong CYP3A4 inhibitors, may also potentiate angioedema; increased rates of biopsy-confirmed acute rejection (BCAR) in renal transplant patients have been observed with concomitant use of SIR with ACE inhibitors	continued coadministration of CsA and SIR as maintenance therapy cannot be recommended considering increased nephrotoxicity and malignancies; SIR, MMF, and corticosteroids, in combination with IL-2 receptor antibody (IL2R Ab) induction, is not recommended in the de novo renal transplant setting; **not with strong inhibitors of CYP3A4 (such as ketoconazole, voriconazole, itraconazole, telithromycin, or clarithromycin) which severely increase SIR exposure; not with inducers of CYP3A4 (such as rifampin and rifabutin), rifampicin increased the clearance of SIR by approximately 5.5-fold, coadministration of SIR, and rifampicin is not recommended;** vaccination may be less effective, the use of live vaccines should be avoided during SIR; safety and efficacy of SIR as immunosuppressive therapy have not been established in liver or lung transplant patients, and therefore such use is not recommended	dose reduction in hepatic impairment; periodic quantitative monitoring of proteinuria; conversion from the TAC to SIR in maintenance renal transplant patients was associated with an unfavorable safety profile without efficacy benefits, and can therefore not be recommended; in hepatically impaired patients, SIR whole blood trough levels to be closely monitored, in patients with severe hepatic impairment, reduction in maintenance dose by one-half is recommended with close monitoring after a loading dose or a change of dose for a prolonged period of time, until stable concentrations are reached; in de novo liver transplant patients, the use of SIR plus CsA or TAC was associated with an increase in hepatic artery thrombosis, mostly leading to graft loss or death; a clinical study in liver transplant patients randomized to conversion from a CNI-based regimen to a SIR-based regimen versus continuation of a CNI-based regimen 6-144 months post-liver transplantation failed to demonstrate superiority in baseline-adjusted GFR at 12 months; cases of bronchial anastomotic dehiscence, most fatal, have been reported in de novo lung transplant patients when SIR has been used as part of an immunosuppressive regimen
**Sitagliptin** [103]	acute pancreatitis; hypoglycemia when used in combination with other anti-hyperglycemic drug (e.g., sulphonylurea, insulin); headache	CYP3A4 metabolism may play a role in the elimination of sitagliptin in the setting of severe renal impairment or end-stage renal disease (ESRD); for this reason, potent CYP3A4 inhibitors (i.e., ketoconazole, itraconazole, ritonavir, clarithromycin) might increase exposure	should not be used in patients with type 1 diabetes or for the treatment of diabetic ketoacidosis	**dose adjustment based upon renal function**, the assessment of renal function is recommended prior to initiation and periodically thereafter
**Sulfamethoxazole/Trimethoprim** [104]	hyperkalemia and hyponatremia; metabolic acidosis; headache; nausea, diarrhea; caution in patients with severe atopy or bronchial asthma; very rare, due to severe Stevens-Johnson syndrome, toxic epidermal necrolysis, fulminant hepatic necrosis, agranulocytosis, aplastic anemia, other blood dyscrasias, and hypersensitivity of the respiratory tract; very rarely hemophagocytic lymphohistiocytosis (HLH); very rarely respiratory toxicity; very rarely sulphonamide crystals are noted in cooled urine, in patients suffering from malnutrition, the risk may be increased; asymptomatic changes in hematological laboratory indices due to the lack of available folate, supplementation with folinic acid may be considered, but this should be initiated with caution due to possible interferences with antimicrobial efficacy; overgrowth fungal; jaundice, cholestatic, and hepatic necrosis; cough, dyspnea and lung infiltration; rhabdomyolysis reported in HIV patients with prophylaxis or the treatment of PJP	deterioration in renal function with CsA; zidovudine may increase the risk of hematological adverse reactions; possibility of competitive inhibition and increased exposure with drugs that form cations at physiological pH, and are also partly excreted by active renal secretion (e.g., procainamide, amantadine), there is the possibility of competitive inhibition; elderly patients concurrently receiving diuretics, mainly thiazides, appear to be at an increased risk of thrombocytopenia; megaloblastic anemia with pyrimethamine; potentiates the anticoagulant activity of warfarin; increased exposure of phenytoin; increased digoxin exposure; may increase the free plasma levels of methotrexate; with methotrexate, a folate supplement should be considered; increased lamivudine exposure; likely potentiation with sulphonylurea hypoglycemic agents; risk of hyperkalemia with ACE inhibitors, angiotensin receptor blockers, and potassium-sparing diuretics, such as spironolactone; increased exposure of repaglinide (hypoglycemia); folinic acid supplementation interferes with the antimicrobial efficacy; with azathioprine risk of serious hematological abnormalities; oral contraceptive failures have been reported with antibiotics	not in severe hepatic impairment; not in severe renal insufficiency; not with a history of drug-induced immune thrombocytopenia with the use of trimethoprim and/or sulphonamides; except under careful supervision, it should not be given to patients with serious hematological disorders	care is always advisable in elderly patients due to higher levels of susceptibility to ADRs, e.g., with impaired kidney and/or liver function and/or concomitant use of other drugs; effects associated with high dose pneumocystis jirovecii pneumonitis (PJP) management are severe hypersensitivity reactions, rash, pyrexia, neutropenia, thrombocytopenia, hepatic enzyme increased, hyperkalemia, hyponatremia, rhabdomyolysis
**Tacrolimus (TAC)** [2]	**acute renal impairment without active intervention may progress to chronic renal impairment**, patients with impaired renal function should be monitored closely as the dosage of TAC may need to be reduced, the risk for nephrotoxicity may increase when TAC is administered with drugs associated with nephrotoxicity; renal failure, renal failure acute, oliguria, renal tubular necrosis, nephropathy toxic, urinary abnormalities, bladder, and urethral symptoms; hypertension, ischemic coronary artery disorders, tachycardia; hemorrhage, thromboembolic, and ischemic events, peripheral vascular disorders, vascular hypotensive disorders; increased risk for infections (viral, bacterial, fungal, protozoal), the course of preexisting infections may be aggravated, both generalized and localized infections can occur, CMV infection, BK virus-associated nephropathy, JC virus-associated progressive multifocal leukoencephalopathy (PML); gastrointestinal disorders incl. diarrhea, nausea, gastrointestinal inflammatory conditions, gastrointestinal ulceration and perforation, gastrointestinal hemorrhages, stomatitis and ulceration, ascites, vomiting, gastrointestinal and abdominal pains, dyspeptic signs and symptoms, constipation, flatulence, bloating and distension, and loose stools; TAC blood levels may significantly decrease during diarrhea episodes; anemia, leukopenia, thrombocytopenia, leukocytosis, red blood cell analyses may be abnormal; dyspnea; benign as well as malignant neoplasms including EBV-associated lymphoproliferative disorders, skin malignancies; parenchymal lung disorders, pleural effusion, pharyngitis, cough, nasal congestion, and inflammations; pain in lower extremities reported as calcineurin-inhibitor induced pain syndrome (CIPS); primary graft dysfunction with medication errors; abnormal liver function tests, cholestasis and jaundice, hepatocellular damage and hepatitis, cholangitis; asthenic conditions, febrile disorders, oedema, pain and discomfort, body temperature perception disturbed; arthralgia, muscle spasms, pain in extremity, back pain; pruritus, rash, alopecia, acne, sweating increased; tinnitus; vision blurred, photophobia, eye disorders; tremor, headache, seizures, disturbances in consciousness, paraesthesia and dysesthesia, peripheral neuropathies, dizziness, writing impaired, nervous system disorders; insomnia, anxiety symptoms, confusion and disorientation, depression, depressed mood, mood disorders and disturbances, nightmares, hallucinations, mental disorders; hyperglycemic conditions, diabetes mellitus, hyperkalemia, hypomagnesemia, hypophosphatemia, hypokalemia, hypocalcemia, hyponatremia, fluid overload, yperuricemia, decreased appetite, metabolic acidosis, hyperlipidemia, hypercholesterolemia, hypertriglyceridemia, other electrolyte abnormalities	**CYP3A4 inhibitors** increase TAC blood levels, which could lead to serious adverse reactions, including **nephrotoxicity, neurotoxicity, and QTc prolongation;** increase in TAC blood levels when coadministered with inhibitors of CYP3A4 is mainly a result of an increase in the oral bioavailability of TAC, owing to the inhibition of gastrointestinal metabolism, the effect on hepatic clearance is less pronounced; **P-glycoprotein inhibitors increase TAC blood levels; CYP3A4 inducers reduce TAC exposure;** high-potassium intake or potassium-sparing diuretics should be avoided; TAC with drugs known to have neurotoxic effects may increase the risk of these effects; the risk for nephrotoxicity may increase when TAC is administered with drugs associated with nephrotoxicity, combination should be avoided; with MMF, caution when switching combination therapy from CsA, which interferes with enterohepatic recirculation reducing MPA, to TAC, which is devoid of this effect, resulting in elevated mycophenolic acid exposure; as TAC may be associated with hyperkalemia, or may increase pre-existing hyperkalemia, high-potassium intake, or potassium-sparing diuretics (e.g., amiloride, triamterene, or spironolactone) should be avoided, care when TAC is coadministered with other agents that increase serum potassium, such as trimethoprim and cotrimoxazole (trimethoprim/sulfamethoxazole), as trimethoprim acts as a potassium-sparing diuretic like amiloride, the close monitoring of serum potassium is recommended; concomitant administration TAC with SIR or EVR may increase the risk of thrombotic microangiopathy (incl. hemolytic uremic syndrome and thrombotic thrombocytopenic purpura); enhanced nephrotoxic or neurotoxic effect with aminoglycosides, gyrase inhibitors, vancomycin, sulfamethoxazole and trimethoprim, NSAIDs, ganciclovir, acyclovir, cidofovir, foscarnet, amphotericin B; avoid grapefruit or grapefruit juice associated with TAC increase; moderate or weak CYP3A4 inhibitors: antifungal agents (e.g., fluconazole, isavuconazole, clotrimazole, miconazole), the macrolide antibiotics (e.g., azithromycin), calcium channel blockers (e.g., nifedipine, nicardipine, diltiazem, verapamil), amiodarone, danazol, ethinylestradiol, lansoprazole, omeprazole, the HCV antivirals elbasvir/grazoprevir and glecaprevir/pibrentasvir, the CMV antiviral letermovir, and the tyrosine kinase inhibitors nilotinib, crizotinib, imatinib, and (Chinese) herbal remedies containing extracts of *Schisandra sphenanthera* may increase TAC whole blood trough concentrations and increase the risk of serious ADRs (e.g., neurotoxicity, QTc prolongation); in vitro, the following substances have been shown to be potential inhibitors of TAC metabolism: bromocriptine, cortisone (although in vivo, we also found a decrease in TAC and CsA with cortisone), dapsone, ergotamine, gestodene, lidocaine, mephenytoin, midazolam, nilvadipine, norethisterone, quinidine, tamoxifen; these may increase TAC whole blood trough concentrations and increase the risk of serious adverse reactions (e.g., neurotoxicity, QTc prolongation); graft function closely; **moderate CYP3A4 inducers: metamizole**, phenobarbital, isoniazid, rifabutin, efavirenz, etravirine, nevirapin, and weak CYP3A4 inducers: flucloxacillin, **decrease TAC whole blood trough concentrations and increase the risk of rejection, it is strongly recommended to closely monitor TAC trough levels during treatment and up to 2 weeks after discontinuation**; cannabinol increases TAC exposure; DDI with drugs with high affinity for plasma proteins, e.g.,: NSAIDs, oral anticoagulants, oral antidiabetics since TAC is extensively bound to plasma proteins; prokinetic agents: metoclopramide, cimetidine, and magnesium-aluminum-hydroxide may increase TAC whole blood trough concentrations; maintenance doses of corticosteroids decrease TAC whole blood trough concentrations and increase the risk of rejection; direct-acting antiviral (DAA) therapy may have an impact on the pharmacokinetics of TAC by impacting liver function during DAA therapy, related to the clearance of hepatitis virus, a decrease in TAC blood levels may occur; however, the CYP3A4-inhibiting potential of some DAAs may counteract that effect or lead to increased TAC blood levels; TAC is a CYP3A4 inhibitor, thus, the concomitant use of TAC with drugs known to be metabolized by CYP3A4 may affect the metabolism of such drugs, e.g., an increase in the exposure of CsA, phenytoin, steroid-based contraceptives, leading to increased hormone exposure; particular care should be exercised when deciding upon contraceptive measures; limited knowledge of **interactions between TAC and statins is available (see** Section 3.3.8 **for more information);** pentobarbital and phenazone half-life is increased	the half-life of CsA is prolonged when TAC is given concomitantly, in addition, synergistic/additive nephrotoxic effects, **coadministration of CsA and TAC is not recommended**; medication errors, including inadvertent, unintentional, or unsupervised substitution of the immediate- or prolonged-release TAC formulations, have been observed with serious adverse events, including graft rejection, or other side effects, which could be a consequence of either under- or overexposure to TAC, patients should be maintained on a single formulation of TAC with the corresponding daily dosing regimen, **alterations in formulation or regimen should only take place under the close supervision of a transplant specialist;** during the initial posttransplant period, monitor blood pressure, ECG, neurological and visual status, fasting blood glucose levels, electrolytes (particularly potassium), liver and renal function tests, hematology parameters, coagulation values, and plasma protein determinations for dose adjustments; strong CYP3A4 inhibitors: antifungal agents (e.g., ketoconazole, itraconazole, posaconazole, voriconazole), the macrolide antibiotics (e.g., telithromycin, troleandomycin, clarithromycin, josamycin, erythromycin), HIV protease inhibitors (e.g., ritonavir, nelfinavir, saquinavir), HCV protease inhibitors (e.g., telaprevir, boceprevir, and the combination of ombitasvir and paritaprevir with ritonavir, when used with and without dasabuvir), nefazodone, the pharmacokinetic enhancer cobicistat, and the kinase inhibitors idelalisib and ceritinib should be avoided, if unavoidable, monitor TAC blood levels, renal function, blood pressure, ECG, QTC intervals, and the clinical condition of the patient and adjust immediately, overall TAC exposure may increase >5-fold, with ritonavir >50-fold; the effect on TAC blood concentrations may remain for several days after coadministration is completed; similarly, the discontinuation of CYP3A4 inhibitors may lead to subtherapeutic blood levels of TAC, and therefore requires the close monitoring and supervision of a transplant specialist; CYP3A4 inducers decrease TAC blood levels, potentially increasing the risk of transplant rejection, the recommended that the concomitant use of strong CYP3A4 inducers (such as rifampicin, phenytoin, carbamazepine apalutamide, enzalutamide, mitotane, or St. John’s wort) with TAC should be avoided; if unavoidable, TAC blood levels and graft function should be monitored closely, maximal effect on TAC blood concentrations may be achieved after 1–2 weeks (we find about 5 days) after coadministration, and may remain for 1–2 weeks (we find about 5 days) after the completion of the treatment; similarly, the discontinuation of CYP3A4 inducers may lead to supratherapeutic blood levels TAC, and therefore requires the close monitoring and supervision of a transplant specialist; St. John’s wort or other herbal preparations should be avoided, due to decreases in TAC blood concentrations and reduced clinical effect, or an increase in TAC blood concentrations and TAC toxicity; not with CsA	27% bioavailability decrease with food consumption, administer at least 1 h before or 2 h after meals; grapefruit juice increases exposure, St. John’s wort decreases exposure, TAC target whole blood trough concentration recommendations; dosing should primarily be based on clinical assessments of rejection and tolerability in each individual patient; to optimize dosing, several immunoassays are available for determining TAC concentrations in the whole blood, comparisons of concentrations from the published literature to individual values in clinical practice should be assessed with the care and knowledge of the assay methods employed; when dosed orally, blood trough levels should be drawn approximately 12 h post-dosing, just prior to the next dose; the frequency of blood level monitoring should be based on clinical needs, as TAC is with low clearance; adjustments to the dosage regimen may take several days before changes in blood levels are apparent; vaccination during treatment with TAC may be less effective, the use of live attenuated vaccines should be avoided
**Tamsulosin** [105]	**hypotension, rarely syncope** can occur, at the first signs of orthostatic hypotension (dizziness, weakness), the patient should sit or lie down until the symptoms have disappeared; dizziness, headache; palpitations; ejaculation disorder; angioedema; ‘intraoperative floppy iris syndrome’ (IFIS, a variant of small pupil syndrome), benefits of treatment discontinuation have not yet been established	**strong inhibitors of CYP3A4 may lead to the increased exposure of tamsulosin hydrochloride**, e.g., with ketoconazole 2.8-fold AUC, caution in combination with strong and moderate inhibitors of CYP3A4; diclofenac and warfarin may increase the elimination rate; concurrent other α_1_-adrenoceptor antagonists could lead to hypotensive effects	not with a history of orthostatic hypotension, **not in severe hepatic insufficiency**, micturition syncope history; not to be given in combination with strong inhibitors of CYP3A4 in patients with poor metabolizer CYP2D6 phenotype	caution with severe renal impairment (creatinine clearance of <10 mL/min), not yet studied
**Ticagrelor** [106]	blood disorder bleedings; hyperuricemia, gout/gouty arthritis; dyspnea (with asthma/chronic obstructive pulmonary disease (COPD) increased absolute risk of experiencing dyspnea), respiratory system bleedings; dizziness, syncope, headache; vertigo; hypotension; gastrointestinal hemorrhage; diarrhea, nausea, dyspepsia, constipation; urinary tract bleeding; blood creatinine increased (attention to patients ≥ 75 years, patients with moderate/severe renal impairment, and those receiving concomitant treatment with an angiotensin receptor blocker); post-procedural hemorrhage, traumatic bleedings; **bradyarrhythmia events and AV block** (primarily in patients with ACS, where cardiac ischemia and concomitant drugs reducing the heart rate or affecting cardiac conduction are potential confounders), ventricular pauses with ticagrelor were higher in patients with chronic heart failure (CHF); central sleep apnea including Cheyne–Stokes respiration; thrombotic thrombocytopenic purpura (TTP)	caution with the concomitant administration of medicinal products that may increase the risk of bleeding (e.g NSAIDs, oral anticoagulants and/or fibrinolytics); antifibrinolytic therapy (aminocaproic acid or tranexamic acid) and/or recombinant factor VIIa therapy may increase haemostasias; caution with bradycardia-inducing drugs; moderate CYP3A4 inhibitors (e.g., amprenavir, aprepitant, diltiazem, erythromycin, and fluconazole) only slightly change AUC; twofold-increase with grapefruit juice; decreased exposure with CYP3A inducers (e.g., rifampicin, phenytoin, carbamazepine, and phenobarbital), do not combine; **caution increased exposure with potent P-gp inhibitors and moderate CYP3A4 inhibitors (e.g., CsA,** verapamil, quinidine); reduced ticagrelor efficacy in patients who have been coadministered ticagrelor and morphine (not with parenteral); haemostasias altering drugs with caution in combination (with heparin, enoxaparin, and ASA or desmopressin possible); not with doses of simvastatin or lovastatin > 40 mg; not with CYP3A4 substrates with narrow therapeutic indices (i.e., cisapride or ergot alkaloids); monitoring in narrow therapeutic index P-gp-dependent drugs like digoxin; might reduce the renal excretion of rosuvastatin; **cutaneous bleeding abnormalities with SSRIs** (e.g., paroxetine, sertraline, and citalopram)	not with active pathological bleeding; history of intracranial hemorrhage; not with severe hepatic impairment; **substantial increase in exposure with strong CYP3A4 inhibitors** (e.g., ketoconazole, clarithromycin, nefazodone, ritonavir, and atazanavir)	discontinue 5 days prior to surgery; platelet transfusion did not reverse the antiplatelet effect, unlikely to be of clinical benefit in patients with bleeding; desmopressin unlikely to be effective in managing clinical bleeding events; caution in moderate hepatic impairment, in sick sinus syndrome, 2nd or 3rd degree AV block, or bradycardic-related syncope; false negative results in a platelet function test (to include, but may not be limited to the HIPA test) for HIT have been reported in patients administered ticagrelor
**Tigecycline** [107]	**prolonged both prothrombin time (PT) and activated partial thromboplastin time (aPTT), hypofibrinogenemia;** nausea, vomiting, diarrhea; impaired healing; in complicated skin and soft tissue infections (cSSTI), complicated intra-abdominal infections (cIAI), diabetic foot infections, nosocomial pneumonia, and studies in resistant pathogens, a numerically higher mortality rate among tigecycline-treated patients has been observed as compared to the comparator treatment; liver injury with a predominantly cholestatic pattern up to hepatic failure; photosensitivity, pseudotumor cerebri, pancreatitis, and anti-anabolic action with increased BUN, azotemia, acidosis, and hyperphosphatemia; acute pancreatitis; AST and ALT abnormalities, hyperbilirubinemia	tigecycline is a P-gp substrate—the coadministration of P-gp inhibitors (e.g., ketoconazole or CSA) or P-gp inducers (e.g., rifampicin) could affect the pharmacokinetics of tigecycline; **increase in trough concentrations of CsA and TAC**; as it may prolong both the prothrombin time (PT) and activated partial thromboplastin time (aPTT), the relevant coagulation tests should be closely monitored when tigecycline is coadministered with anticoagulants	not in children < 8 years because of teeth discoloration	only where alternative antibiotics are not suitable; caution and monitor response with severe hepatic impairment (Child Pugh C), the dose of tigecycline should be reduced by 50%; the impaired healing of the surgical wound has been associated with superinfection, e.g., nosocomial pneumonia associated with poorer outcomes; there were a limited number of patients with severe underlying disease, such as immune-compromi-sed patients, patients with APACHE II scores > 15 (3.3%), or with surgically apparent multiple intra-abdominal abscesses (11.4%); limited experience is also available in treating patients with concurrent bacteremia (5.6%), therefore, caution is advised when treating such patients; consideration should be given to the use of combination antibacterial therapy whenever tigecycline is to be administered to severely ill patients with cIAI secondary to clinically apparent intestinal perforation or patients with incipient sepsis or septic shock; biliary excretion accounts for approximately 50% of the total tigecycline excretion, monitor in cholestasis
**Valganciclovir (see Ganciclovir)** [62,108]				to ensure maximal oral absorption, administer with or immediately after a meal
**Vancomycin i.v.** [109,110]	nephrotoxicity, ototoxicity from high serum concentrations, transitory or permanent, elderly more susceptible; monitor auditory function esp. in the elderly; rapid bolus administration may be associated with exaggerated hypotension, including shock and, rarely, cardiac arrest, histamine-like responses and maculopapular or erythematous rash (“red man’s syndrome”); **thrombocytopenia, neutropenia, agranulocytosis**, eosinophilia; phlebitis, redness of the upper body and the face, pain and spasms in the chest and back muscles	with an aminoglycoside (e.g., gentamycin), patients should be monitored carefully for signs of neurotoxicity and ototoxicity; dosage to be adjusted when renal disturbance occurs; the concurrent, sequential systemic, or topical use of other potentially neurotoxic or nephrotoxic drugs, such as gentamycin, amphotericin B, streptomycin, neomycin, kanamycin, amikacin, tobramycin, bacitracin, polymyxin B, colistin, viomycin, or cisplatin, may potentiate the nephrotoxicity and/or ototoxicity of vancomycin, and consequently requires careful monitoring; increased potential of neuromuscular blockade with concomitant neuromuscular blocking agents	should be avoided in patients with previous hearing loss; avoid the concomitant administration of vancomycin and anesthetic agents with risk of histamine-like flushing and anaphylactoid reactions	infusions should be given over at least 60 min; TDM—the measurement of levels to aid dose adjustments; dose adjustment and regular monitoring of renal function, neutropenia, agranulocytosis, eosinophilia, thrombocytopenia, pancytopenia
**Voriconazol** [111,112]	most common symptoms are as follows: visual impairment, pyrexia, rash, vomiting, nausea, diarrhea, headache, peripheral oedema; cheilitis, dyspepsia, constipation, gingivitis; liver function test abnormalities, respiratory distress, and abdominal pain; sinusitis; agranulocytosis, pancytopenia, thrombocytopenia, leukopenia, anemia; oedema peripheral; hypoglycemia, hypokalemia, hyponatremia; depression, hallucination, anxiety, insomnia, agitation, confusional state; convulsion, syncope, tremor, hypertonia, paresthesia, somnolence, dizziness; hypotension, phlebitis; arrhythmia supraventricular, tachycardia, bradycardia; QTc prolongation, risk of torsades de pointes increased with a history of cardiotoxic chemotherapy, cardiomyopathy, hypokalemia, hypomagnesemia, hypocalcemia, and concomitant QTc-prolonging drugs; caution with potentially proarrhythmic conditions, such as congenital or acquired QTc prolongation, cardiomyopathy, particularly when heart failure is present, sinus bradycardia, existing symptomatic arrhythmias; acute respiratory distress syndrome, pulmonary oedema; jaundice, jaundice cholestatic, hepatitis, hepatic toxicity, primarily in patients with serious underlying medical conditions (predominantly hematological malignancy); transient hepatic reactions, including hepatitis and jaundice, among patients with no other identifiable risk factors, must be carefully monitored for hepatic toxicity; phototoxicity, avoid exposure to direct sunlight and use protection; squamous cell carcinoma of the skin (including cutaneous SCC in situ, or Bowen’s disease); adrenal insufficiency (the direct inhibition of steroidogenesis); **non-infectious periostitis (skeletal pain) with elevated fluoride and alkaline phosphatase levels has been reported in transplant patients;** blurred vision, optic neuritis, and papilledema, retinal hemorrhage; pyrexia; chest pain, face oedema, asthenia, chills; acute renal failure, more likely with nephrotoxic drugs and concurrent conditions that may result in decreased renal function, monitor renal function, hematuria; with risk factors for acute pancreatitis (e.g., recent chemotherapy, hematopoietic stem cell transplantation [HSCT], serum amylase, or lipase should be monitored closely; hepatic function should be monitored	increased exposure of CsA and TAC with risk of renal impairment, **dose be halved in CsA and reduced to one third in TAC, closely monitor levels,** with the discontinuation of voriconazole, increase the CsA and TAC doses as necessary, according to TDM; **increased exposure of NSAIDs (up to 100% in ibuprofen AUC) requires dose reduction, avoid NSAIDs generally because of additive nephrotoxicity;** adrenal insufficiency in patients receiving azoles with corticosteroids (corticosteroid excess and adrenal suppression), Cushing’s syndrome with and without subsequent adrenal insufficiency has been reported with corticosteroids; on long-term treatment with corticosteroids (including inhaled corticosteroids, e.g., budesonide and intranasal corticosteroids) carefully monitor for adrenal cortex dysfunction, both during treatment and when voriconazole is discontinued; monitoring of phenytoin levels; increased glasdegib plasma concentrations with risks of QTc; increased tyrosine kinase inhibitor plasma concentrations and the risk of ADRs; monitoring of full blood counts ADRs to rifabutin (e.g., uveitis), combination should be avoided; low-dose ritonavir (100 mg twice daily) should be avoided; with methadone QTc prolongation, a dose reduction of methadone is recommended; reduction in the dose of alfentanil, fentanyl, and other short-acting opiates similar in structure to alfentanil and metabolized by CYP3A4 (e.g., sufentanil) should be considered; the half-life of alfentanil is prolonged fourfold; frequent monitoring for opiate-associated adverse reactions (including a longer respiratory monitoring period); where there are reductions in the dose of oxycodone and other long-acting opiates metabolized by CYP3A4 (e.g., hydrocodone), monitoring for opiate-associated adverse reactions may be necessary; oral fluconazole resulted in a significant increase in voriconazole exposure, monitoring for voriconazole-associated ADRs if voriconazole is used sequentially after fluconazole; **decreased exposure (about 50%) with Letermovir, monitor for loss of voriconazole effectiveness**; with flucloxacillin, decrease exposure; tyrosine kinase inhibitors (e.g., axitinib, bosutinib, cabozantinib, ceritinib, cobimetinib, dabrafenib, dasatinib, nilotinib, sunitinib, ibrutinib, ribociclib) require dose reduction because of increased exposure with voriconazole; warfarin and other oral coumarins monitor for increased prothrombin time to adjust; increased exposure of ivacaftor recommends dose reduction; **increased exposure of midazolam and other benzodiazepines** with a prolonged sedative effect require dose reduction; with increased PPI exposure, doses are probably to be halved; increased oral contraceptives exposure besides voriconazole exposure, monitor for ADRs; **CYP3A4 metabolized statins (simvastatin, lovastatin) require dose reduction because of the risk of rhabdomyolysis**; increased sulfonylureas (e.g., tolbutamide, glipizide, glyburide), dose reduction due to the risk of hypoglycemia; increased exposure of vinca alkaloids (e.g., vincristine and vinblastine) requires dose reduction, due to the risk of neurotoxicity; this may inhibit the metabolism of HIV protease inhibitors and the metabolism of voriconazole may also be inhibited by HIV protease inhibitors, dose adjustment will be required; with other non-nucleoside reverse transcriptase inhibitors (NNRTIs) (e.g., delavirdine, nevirapine) not studied, monitor for toxicity and inefficacy, as well to adjust; increase in tretinoin concentrations risk of pseudotumor cerebri, hypercalcemia; the effect on either erythromycin or azithromycin is unknown	not with CYP3A4 substrates, terfenadine, astemizole, cisapride, pimozide, or quinidine, due to QTc prolongation and the risk of torsades de pointes; not with rifampicin, carbamazepine, phenobarbital, and St. John’s Wort because of the decrease in plasma voriconazole exposure; not with efavirenz because of the decreased plasma voriconazole exposure and increased efavirenz; not with high-doses of ritonavir (400 mg and above twice daily) because of the decreased voriconazole exposure; not with ergot alkaloids (ergotamine, dihydroergotamine), which are CYP3A4 substrates, as this can lead to ergotism; **not with SIR or EVR because of significantly elevated SIR or EVR exposure**; not with naloxegol, because the increased naloxegol exposure precipitates opioid withdrawal symptoms; not with tolvaptan, lurasidone, because of the increased exposure; not with apixan, edoxaban, and rivaroxaban; caution with dabigatran due to the increased exposure and bleeding risk	is metabolized by, and inhibits the activity of, CYP2C19, CYP2C9, and, strongly, CYP3A4; must be carefully monitored for hepatic toxicity including laboratory evaluation of hepatic function (specifically AST and ALT) at the initiation of treatment and at least weekly for the first month of treatment; treatment duration should be as short as possible; long-term exposure (treatment or prophylaxis) greater than 6 months requires careful assessment of the benefit–risk balance, e.g., squamous cell carcinoma of the skin (SCC) (including cutaneous SCC in situ, or Bowen’s disease) has been reported in relation to long-term treatment; monitor renal function; to ensure maximal oral absorption, voriconazole tablets and oral suspension should be taken at least one hour before or after a meal
**Zolpidem** [113]	myorelaxant effect with a risk of falls and consequent injuries, particularly for elderly patients when they are active at night; anxiety or agitation have been described as signs of decompensated respiratory insufficiency; tolerance with some loss of efficacy to the hypnotic effects of short-acting benzodiazepines and benzodiazepine-like agents may develop after repeated use for a few weeks; abuse and physical and psychological dependence; withdrawal symptoms; rebound insomnia; decrease the dose gradually; somnambulism—sleep walking and other associated behaviors; next-day psychomotor impairment further increased with other CNS depressants or with other drugs that increase the blood levels of zolpidem, or with alcohol or illicit drugs; anterograde amnesia—ensure that they will be able to have an uninterrupted sleep of 8 h; psychiatric and “paradoxical” reactions more likely in the elderly; drowsiness and a decreased level of consciousness, which may lead to falls and consequently to severe injuries; consider congenital long QTc syndrome; may precipitate encephalopathy; increased incidence of suicidal ideation, suicide attempts, and suicide in patients with or without depression; pre-existing depression may be disclosed during any use of zolpidem, since insomnia may be a symptom of depression; the patient should be revaluated if insomnia persists; respiratory tract infection; hallucinations, agitation, nightmares, depression; somnolence, drowsiness during the following day, numbed emotions, reduced alertness, headache, dizziness, ataxia, exacerbated insomnia, cognitive disorder, amnesia; vertigo	concomitant opioids may result in sedation, respiratory depression, coma, and death; the enhancement of the central depressive effect may occur in cases of concomitant use with antipsychotics (neuroleptics), hypnotics, anxiolytics/sedatives, muscle relaxants, antidepressant agents, narcotic analgesics, antiepileptic drugs, anesthetics, and sedative antihistamines may increase drowsiness and next-day psychomotor impairment; **visual hallucinations in patients taking zolpidem with antidepressants including bupropion, desipramine, fluoxetine, sertraline, and venlafaxine**; in the case of narcotic analgesics, the enhancement of euphoria may also occur, leading to an increase in psychological dependence; rifampicin, carbamazepine, phenytoin, and St. John’s wort decrease blood levels of zolpidem; **CYP3A4 inhibitors (ketoconazole) increase plasma concentrations and require dose reduction**	**contraindicated in patients with severe hepatic impairment**; 5 mg in mild-to-moderate hepatic impairment; not with sleep apnea syndrome, previously known complex sleep behaviors after taking zolpidem, myasthenia gravis, acute or severe respiratory insufficiency; not with alcohol; not with fluvoxamine; **not with ciprofloxacin due to increased blood levels**	**only indicated when insomnia is severe, disabling, or subjecting the individual to extreme distress**; the duration of treatment varies from a few days to two weeks with a maximum, including the tapering-off process of four weeks; the tapering off process should be tailored to the individual; a single intake, should not be re-administered during the same night, lowest effective daily dose of zolpidem should be used and must not exceed 10 mg; in elderly or debilitated patients who may be especially sensitive to the effects of zolpidem, a dose of 5 mg is recommended; psychotic illness benzodiazepines and benzodiazepine-like agents not recommended for the primary treatment; extreme caution in patients with a history of alcohol or drug abuse; **in elderly patients, the bioavailability of zolpidem is increased with reduced clearance, the maximal plasma concentration is increased by approximately 80% in a patient group aged 81–95 years**; **in hepatic impairment, the bioavailability of zolpidem is increased by 80%, and the half-life is increased from 2.4 h in healthy individuals to 9.9 h;** in patients with liver cirrhosis, a fivefold increase in AUC and a threefold increase was observed in the half-life
**Others**		**Interactions**	**CI**	**Other Aspects**
**Red blood cell concentrate** [114]		**increased blood levels of CsA, TAC, SIR, EVR**		
**Grapefruit juice**		**increased exposure of CsA, TAC, SIR, EVR,** simvastatin, atorvastatin, lovastatin		
**St. John’s wort**		**decreased exposure of CsA, TAC, SIR, EVR**, simvastatin, atorvastatin, lovastatin		
**Tyramine-rich foods** [115]			red wine, many beers, aged cheeses, processed meats, and smoked fish; chocolate containing an amino acid derivative called tyramine—not with monoamine oxidase inhibitors, such as tranylcypromine, selegiline and phenelzine, or linezolid, due to the risk of hypertensive crisis	
**Vaccination** [116]			never apply live vaccines with immunosuppressants; live vaccines include the nasal flu vaccine, measles, mumps, rubella vaccines, small pox vaccine, and the chicken pox vaccine (but not the Shingrix/zoster vaccine); the oral polio vaccine is contraindicated in patients and household members; vaccinia is contraindicated in patients, household members, and healthcare workers in transplant centers	
**Ginkgo biloba** [117]		may influence the effect of anticoagulant drugs, such as warfarin and aspirin NSAIDs, and may potentiate the risk of bleeding; a lack of information on DDIs with DOACs, but since they may modulate P-gp activity, their ability to enhance the anticoagulant potential of DOACs needs to be analyzed; may reduce the effectiveness of antiepileptic drugs, SSRIs, and SNRIs		Ginkgolides, the major chemical components of Ginkgo biloba, have anti-inflammatory and anti-platelet properties
**Cannabis** [118]		CYP3A4 inhibitor ketoconazole nearly doubled the THC and cannabidiol concentrations; similar interactions could occur with other **CYP3A4 inhibitors**, including macrolides and verapamil, thus **augmenting the psychoactive effects of THC and the dose-related adverse effects of cannabidiol (e.g., somnolence, transaminase elevation),** CYP2C9 inhibitors, such as cotrimoxazole, fluoxetine, and amiodarone, would also be expected to increase the THC exposure and psychoactive effects; **cannabidiol inhibits CYP2C19**, increasing levels of the active metabolite of clobazam 3-fold; **threefold increase in TAC** with cannabidiol **shows that CYP3A4/5 inhibition** can also occur. Cave: this would identically affect CsA, SIR, EVR and numerous other drugs, e.g., most statins and calcium channel blockers.		

* intentionally incomplete; preferentially ADRs with reference to clinical transplant conditions. ** intentionally incomplete, e.g., excluding pregnancy, lactation, fertility, children’s aspects, hypersensitivity, intolerance related to congenital disorders, among others.

## Data Availability

There are no datasets generated and analyzed for the concept presented.
